# Nutrition Intervention Coverage and Inequities Along the Continuum of Care: Results From the Eighth Demographic and Health Survey in Six Sub‐Saharan African Countries

**DOI:** 10.1111/mcn.70085

**Published:** 2025-08-29

**Authors:** Erica Phillips, Stephanie Zobrist, Erin M. Milner, Jacqueline K. Kung'u, Rebecca A. Heidkamp, Rukundo K. Benedict

**Affiliations:** ^1^ University of Wisconsin–Madison Madison Wisconsin USA; ^2^ The Demographic and Health Surveys Program, PATH Seattle Washington USA; ^3^ Bureau for Global Health, Maternal and Child Health and Nutrition Office, USAID Washington, D.C. USA; ^4^ African Population and Health Research Center Nairobi Kenya; ^5^ International Health, Johns Hopkins Bloomberg School of Public Health Baltimore Maryland USA; ^6^ The Demographic and Health Surveys Program, ICF Rockville Maryland USA

**Keywords:** data systems, health inequalities, intervention coverage, maternal and child nutrition, nutrition interventions, sub‐Saharan Africa

## Abstract

Many countries rely on national household surveys to monitor coverage of nutrition interventions. Following a multi‐year consultative effort, 14 new and revised nutrition coverage indicators were included in the Round 8 Demographic and Health Survey (DHS‐8) core questionnaire. These indicators were better aligned with international recommendations and generate actionable data for policy and programmatic decision making at national, subnational, and global levels. This analysis highlights their potential applications. We included six sub‐Saharan African countries who collected and released DHS‐8 datasets between January 2021 and June 2024 (Burkina Faso, Côte d'Ivoire, Ghana, Kenya, Mozambique, and Tanzania). We present weighted averages for all nutrition coverage indicators from pregnancy through young childhood by country and estimate inequities in coverage. Coverage of nutrition interventions provided during pregnancy, birth, and postnatal care was higher than during infancy and young childhood, with wide variation between and within countries. For the new indicators on prenatal counseling about breastfeeding and maternal diet, Ghana had the highest coverage (88% and 92%, respectively) and Mozambique the lowest (48% and 51%). Postnatal counseling about infant and young child feeding practices was universally lower, ranging from 12% in Mozambique to 50% in Ghana. Subnational region, wealth quartile, and maternal education were consistent drivers of inequity. The greatest differences in coverage were by subnational region, as high as 71 percentage points for coverage of height and weight measurement of young children in Kenya. The expanded DHS‐8 nutrition indicators fill critical information gaps about coverage and inequalities in care.

## Summary

- The Demographic and Health Survey‐8 core questionnaire was expanded to include a broader range of nutrition intervention coverage indicators.
- Coverage indicators are actionable and pragmatic and facilitate policy and program decision‐makers' ability to respond to intervention challenges, gaps in coverage, and inequities.
- Nutrition intervention coverage along the continuum of care varied substantially within and between the six sub‐Saharan African countries; coverage was highest during pregnancy and lowest during infancy and young childhood.
- The most educated and wealthiest households, especially those in urban areas, had the highest coverage of nutrition services.

## 1 Introduction and Objectives

Equitable delivery of nutrition interventions targeted to women and children along the continuum of care is critical to reach Universal Health Coverage (UHC) and in turn, the World Health Assembly (WHA) Global Nutrition Targets and Sustainable Development Goals (SDGs) (Kerber et al. 2007; United Nations 2022; World Health Organization 2019b, 2024c). Target groups need to receive evidence‐based nutrition interventions at appropriate times to be effective and (Keats et al. 2021) intervention coverage indicators measure receipt of these services at the population level (Keats et al. 2021; Mant 2001; Tanahashi 1978). Data on intervention coverage is more responsive to policy and program actions than data on nutrition outcomes, making them more actionable and pragmatic for national and subnational decision makers (Bryce et al. 2013; Victora et al. 2019).

Demographic and Health Surveys (DHS) and Multiple‐Indicator Cluster Surveys (MICS) are population‐based household surveys that produce standardized data on key populations, including women and children, in low‐ and middle‐income countries (LMICs) (The Demographic and Health Surveys (DHS) Program 2024a; UNICEF 2024). DHS and MICS survey data are nationally representative and conducted at regular intervals of about 3–5 years. They complement health management information system(s) (HMIS) data that report monthly on health system contacts. Although HMIS data are reported more frequently, data quality varies and data are not nationally representative nor easily disaggregated (Mallick et al. 2020). DHS and MICS surveys provide population‐based estimates that can be disaggregated for equity analyses.

While the DHS and MICS have collected data on a wide range of valuable process and outcome measures, these surveys have not captured extensive data on nutrition intervention coverage (International Food Policy Research Institute (IFPRI) 2014). A 2019 analysis by Countdown to 2030 for Women's, Children's, and Adolescents' health initiative identified gaps in coverage data for high‐impact nutrition interventions in both DHS and MICS core questionnaires and recommended inclusion of a broader range of nutrition intervention coverage indicators (Gillespie et al. 2019). The DHS Round 8 core questionnaire (2019–2024) was modified to address these gaps, based on updated research findings and recommendations submitted to The DHS Program by members of the nutrition community (Buckland et al. 2020; DataDENT 2019b). Changes included a) the addition of several nutrition coverage indicators along the continuum of care, from antenatal care (ANC) during pregnancy through early childhood, to better align questions with World Health Organization (WHO) guidelines on Maternal, Infant, and Young Child Nutrition (MICYN) counseling across the first 1000 days, growth monitoring (GM), and screening for severe acute malnutrition (SAM), and b) the revision of multiple questions based on recent indicator validation studies (DataDENT 2019a, 2019b; Kerber et al. 2007; McCarthy et al. 2016; Munos et al. 2018; The DHS Program 2019, 2024b).

The expanded nutrition content in DHS‐8 enables us, for the first time, to examine the coverage of key nutrition interventions within and across countries and provides novel opportunities to identify inequalities in coverage of care. This includes highlighting “opportunity gaps” for improving nutrition intervention coverage by comparing the coverage of the intervention (e.g. iron‐containing supplements) with the coverage of its associated delivery platform (e.g. ANC) (Heidkamp et al. 2020). This analysis can help identify where inputs might have greatest impact to boost coverage of nutrition interventions, as these delivery platforms are in place and already utilized by a proportion of the target population (Nguyen et al. 2022).

Equity analyses, comparing who is and is not being reached by nutrition and health interventions, are other approaches to facilitate decisions around targeted implementation and to reduce persistent inequalities in access and availability of care (Victora et al. 2019; Victora et al. 2021). DHS and MICS country reports include residence (urban/rural) and household wealth quintiles as stratifiers, but do not report their intersectionality. With rapid urbanization in many LMICs, the use of urban poverty, which reflects the combination of residence and wealth variables, could provide context‐specific information about intervention implementation gaps (Assaf et al. 2022; Matthews et al. 2010). This is especially pertinent in the Africa region, which has experienced rapid urbanization and furthest off track to meet the global child nutrition targets, most notably for stunting and wasting (FAO et al. 2023; OECD/UN ECA/AfDB 2022; UNICEF et al. 2023).

The goal of this paper is to highlight the potential use of the expanded nutrition intervention coverage indicators collected in the DHS‐8 by:

1. describing coverage of nutrition interventions and their associated delivery platform along the continuum of care,
2. estimating inequalities in coverage using urban poverty compared to standard equity stratifiers, and
3. presenting a data‐use case study from Kenya for coverage of GM, as an example of how these data can inform policies and strategies to reduce inequalities.

## 2 Methods

Our analysis included the six sub‐Saharan African countries with published DHS‐8 datasets as of June 2024 (Supporting Information S1: Table 1). We included all nutrition intervention coverage indicators along the continuum of care collected in the DHS‐8 core questionnaire ‐ six indicators in pregnancy, two at birth, three in the postnatal period, and seven in infancy and young childhood (Table 1). Recommended nutrition interventions during these life stages include iron supplementation in pregnancy and childhood, counseling about diet and breastfeeding during pregnancy, skin‐to‐skin after birth, counseling and observation of breastfeeding postnatally, IYCF counseling, and GM. Each nutrition indicator is linked to a WHO recommendation and noted if it is also included in the WHO Essential Nutrition Actions (ENA) guidance or the WHA Global Nutrition Monitoring Framework, key global guidance documents for nutrition program implementation (Table 1) (World Health Organization 2019a, 2024b). Coverage of food supplementation or cash assistance during pregnancy was added to DHS‐8 and only included in countries with these programs. It was not included in these country surveys and is therefore not reported here.

**Table 1:** Indicators selected for analysis, aligned with WHO guidelines and global nutrition frameworks.

Nutrition intervention coverage indicator	DHS‐8 status	Population denominator	WHO guideline	Inclusion in Essential Nutrition Actions (ENA) (World Health Organization 2019a) or Global Nutrition Monitoring Framework (GNMF) (World Health Organization 2024b)
Pregnancy				
*Health delivery platform: Attended 4* + *ANC visits*	Existing	Women with a live birth in the 2 years preceding the survey (most recent live birth)	Based on the UNDO/UNFPA/WHO/World Bank (2002) model that has been updated (UNDO/UNFPA/WHO/World Bank 2002)	N/A
*Health delivery platform: Attended 8* + *ANC visits*	New	Women with a live birth in the 2 years preceding the survey (most recent live birth)	WHO recommendations on antenatal care for a positive pregnancy experience, 2016 (World Health Organization 2016)	N/A
Took iron‐containing supplements for any number of days during most recent pregnancy (a)	Revised	Women with a live birth in the 2 years preceding the survey (most recent live birth)	None	GNMF
Took iron‐containing supplements for 90+ days during most recent pregnancy	Revised	Women with a live birth in the 2 years preceding the survey (most recent live birth)	None	ENA ‐ intermittent supplementation recommended dependent on prevalence of anemia in pregnant women
Took iron‐containing supplements for 180+ days during most recent pregnancy	Revised	Women with a live birth in the 2 years preceding the survey (most recent live birth)	WHO, 2016 (World Health Organization 2016); (World Health Organization 2024a)	ENA ‐ all settings
Took intestinal parasite drugs during most recent pregnancy	Existing	Women with a live birth in the 2 years preceding the survey (most recent live birth)	WHO, 2017 (World Health Organization 2017a)	ENA ‐ dependent on the prevalence of soil‐transmitted helminth infection among pregnant women
Counseled about maternal diet during ANC visit (c)	New	Women with a live birth in the 2 years preceding the survey (most recent live birth)	WHO, 2016 (World Health Organization 2016)	ENA ‐ dependent on prevalence of underweight (low body mass index)
Counseled about breastfeeding during ANC visit	New	Women with a live birth in the 2 years preceding the survey (most recent live birth)	WHO, 2017 (World Health Organization 2017b)	ENA ‐ all settings
Birth				
*Health delivery platform: Live births delivered in a health facility*	Existing	Live births in the 2 years preceding the survey	N/A	N/A
Births with skin‐to‐skin contact immediately after birth	Revised	Most recent live births in the 2 years preceding the survey	WHO, 2017 (b) and World Health Organization and the United Nations Children's Fund UNICEF 2018 (World Health Organization 2017b; World Health Organization and the United Nations Children's Fund (UNICEF) 2018)	ENA ‐ all settings
Started breastfeeding within 1 h of birth	Existing	Children who were born in the 2 years preceding the survey	WHO, 2017 (World Health Organization 2017b; World Health Organization and the United Nations Children's Fund (UNICEF) 2018)	ENA ‐ all settings
Postnatal care (PNC) and newborn care				
*Health delivery platform: PNC check within two days for newborn*	Revised	Women with a live birth in the 2 years preceding the survey (most recent live birth)	WHO, 2022 (World Health Organization 2022)	N/A
Weighed during newborn PNC check	Existing	Most recent live births in the 2 years preceding the survey	WHO, 2022 (World Health Organization 2022)	No
Counseled about breastfeeding during newborn PNC check	Revised	Most recent live births in the 2 years preceding the survey	WHO, 2022 (World Health Organization 2022)	ENA ‐ all settings
Observed breastfeeding during newborn PNC check	Revised	Most recent live births in the 2 years preceding the survey	WHO, 2022 (as part of breastfeeding support) (World Health Organization 2022)	ENA ‐ all settings
Infancy and childhood				
Health Delivery platform: All basic vaccinations** according to either source*** (12–35 mos)	Existing	Living children age 12–35 months	WHO, 2024 (World Health Organization 2024d)	N/A
Mothers of children aged 6–23 mos who received IYCF counseling in last 6 mos	New	Women whose youngest child age 6–23 months is living with them	WHO, 2023 (World Health Organization 2023d)	ENA ‐ all settings
Child under 5 with weight measured in the last 3 mos	New	Children age 0–59 months	WHO, 2006 (G. WHO Multicentre Growth Reference Study World Health Organization 2006)	ENA ‐ all settings
Child under 5 with height measured in the last 3 mos	New	Children age 0–59 months	WHO, 2006 (G. WHO Multicentre Growth Reference Study World Health Organization 2006)	ENA ‐ all settings
Child under 5 with MUAC measured in the last 3 mos	New	Children age 0–59 months	WHO, 2023 (World Health Organization 2023e)	ENA ‐ all settings
Children age 6–59 mos given iron‐containing supplements	Revised	Living children age 6–59 months	WHO, 2023 (including malaria endemic regions) (World Health Organization 2023b)	ENA ‐ dependent on the prevalence of anemia
Children age 6–59 mos given Vit. A supplements	Existing	Living children age 6 to 59 months	WHO, 2023 (context specific) (World Health Organization 2023c)	ENA ‐ dependent on the prevalence of night blindness/vitamin A deficiency
Children age 12–59 mos given deworming medication	Existing	Living children age 12 to 59 months	WHO, 2023 (World Health Organization 2023a)	ENA ‐ dependent on the prevalence of soil‐transmitted helminth infection

*Notes:* a‐ Iron‐containing supplements include iron tablets, syrups, and multiple micronutrient powders

b‐A systematic review has been conducted but no current guideline by the Guidelines Review Committee

c‐DHS survey questions ask if a topic was “talked about” and are reported as “counseling” in DHS reports. We use “counseling” for consistency with the reports.

DHS‐8 status – new = newly added to the DHS‐8 core questionnaire, revised = was included in previous rounds of DHS surveys with different working or recall period or phrasing, existing = no change to the question from previous DHS questionnaire

Intervention coverage was calculated as the proportion of women, infants, or children who received an intervention among those who should according to global recommendations. Therefore, the population denominator for each indicator varies depending on how the intervention is targeted. For example, ANC coverage indicators were calculated for women aged 15–49 years with a live birth in the last 2 years whereas IYCF counseling was calculated for the youngest child in the household between 6 and 23 months currently living with the mother with a reference period of 6 months.

For select nutrition indicators, we calculated the opportunity gap based on coverage of a corresponding health system delivery platform through which the intervention is commonly delivered. Health systems indicators included 4 and 8 ANC visits, birth with a skilled provider, a postnatal check within 2 days of birth, and basic vaccine coverage between 12 and 35 months of age. Although 8 ANC visits are the minimum recommendation by WHO, we included 4 ANC visits for comparison, as it continues to be widely reported (World Health Organization 2016).

To examine within country inequalities of nutrition intervention coverage, we used two main approaches. Given our focus on promoting active data use by others, we present illustrative analyses for select indicators rather than all country data sets and indicators. First, we explored how an urban poverty measure compared to separate wealth and the urban/rural residence estimates reported by DHS. The urban poverty measure double stratifies urban households into poor and nonpoor. Poor households are defined as lacking two or more of the following: a) a household made of durable material for the floor, wall, and roof, b) not more than three persons per sleeping room, c) access to improved water, and d) access to improved sanitation (Assaf et al. 2022). The binary urban/rural residence measure is country‐specific and reflects factors including population concentration or density, the percentage of the population involved in agriculture, the availability of electricity or piped water, and the ease of access to health care, schools, or transportation (Croft et al. 2023). We produced equity estimates for the four new or revised MIYCN counseling indicators and present “equity gaps”, the percentage point differences in coverage between these indicators. Statistical significance of the associations across equity stratum was assessed using chi‐squared tests accounting for complex survey design and sampling weights.

Second, we selected one country, Kenya, to describe inequities in coverage of the newly added GM indicators (measurement of weight and height/length) and screening for SAM using mid‐upper arm circumference (MUAC) in children under 5 years of age. We used coverage of these interventions as an example of how these data can be examined to better understand equity gaps and devise specific solutions to target those left behind. We focused on Kenya because of the range of coverage across and between these measures. We included all of the Countdown to 2030 equity stratifiers (household wealth quintile, urban/rural residence, maternal education, maternal age, subnational region, child sex, and child age) and urban poverty (International Center for Equity in Health 2024). We calculated the absolute difference in prevalence for key background characteristics by subtracting the national‐level prevalence estimate from the prevalence observed for a given background characteristic, presented graphically.

All analyses considered each country's survey design and applied survey‐specific sampling weights (adjustment factors) from the datasets to adjust for differences in probability of selection in the survey's sampling frame (Croft et al. 2023). Analyses were performed with Stata 18. Figures were generated in R version 4.2.2.

## 3 Results

### 3.1 Coverage of Health Delivery Platforms and Nutrition Interventions Along the Continuum of Care

#### 3.1.1 Pregnancy

Between 49% (Mozambique) and 88% (Ghana) of women attended at least 4 ANC visits, the delivery platform for nutritional services during pregnancy (Table 2). In all countries except Ghana, however, < 5% of pregnant women attended the minimum recommended 8 ANC visits. At least 80% of women in five of the six countries took any iron‐containing supplements during their previous pregnancy; however, less than 15% of women reported taking iron‐containing supplements for the recommended 180 or more days in all countries except Ghana and Kenya (Lopez de Romaña et al. 2023; World Health Organization 2024a). Intervention coverage for counseling during ANC was between 48% in Mozambique and 92% in Ghana, with counseling about maternal diet reported slightly more frequent than counseling about breastfeeding.

**Table 2:** Coverage of nutrition indicators by health delivery platform.

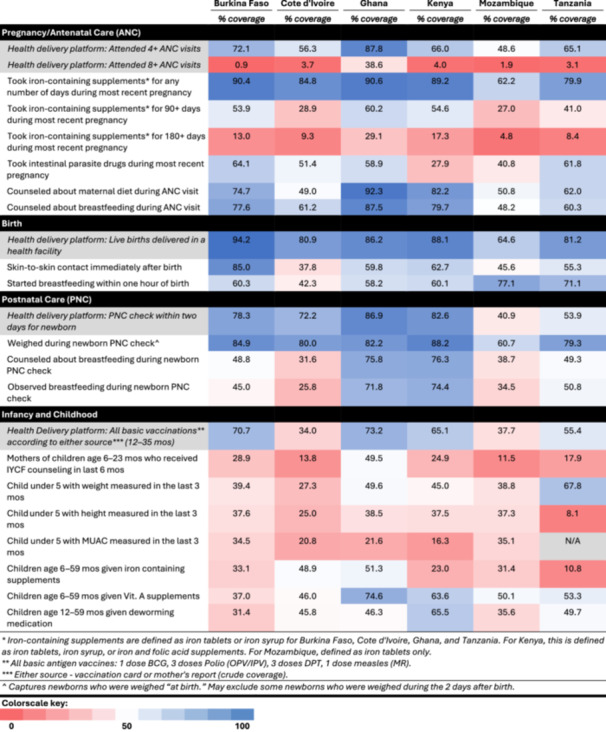

#### 3.1.2 Birth

Between 65% and 94% of women in the six countries delivered in a health facility, the health delivery platform for nutrition interventions at birth. Nutrition intervention coverage varied substantially between countries; only 38% of mothers reported skin‐to‐skin contact in Côte d'Ivoire compared to 85% in Burkina Faso. Early initiation of breastfeeding within 1 h of birth was reported for 42% of infants in Côte d'Ivoire compared to the highest reported coverage of 77% in Mozambique.

#### 3.1.3 PNC

A visit with a provider within 2 days of birth was the health platform for the postnatal period. Coverage of this PNC visit varied between 41% in Mozambique and 87% in Ghana. Weighing the infant was consistently reported by most women across all six countries; however counseling about and observing breastfeeding lagged behind, with roughly 75% coverage in Ghana and Kenya and 26% and 39% coverage in Cote d'Ivoire and Mozambique, respectively.

#### 3.1.4 Infant and Young Childhood

Receipt of basic childhood vaccinations between 12 and 35 months of age was considered the health delivery platform for preventive infant and childhood interventions. Vaccine coverage ranged between 34% in Côte d'Ivoire to 73% in Ghana. Mothers who reported receiving IYCF counseling in the 6 months before the survey varied between 12% in Mozambique and 50% in Ghana. Coverage of GM or screening for SAM differed by type of measurement; weight was more commonly measured than length/height or MUAC. In Tanzania, 68% of mothers reported that their child was weighed in the previous 3 months, compared to 27% in Cote d'Ivoire. Only 8% of children had their length/height measured in Tanzania, with 37%–39% coverage in Burkina Faso, Ghana, Kenya and Mozambique. Coverage of MUAC was assessed in all counties except Tanzania, with coverage below 10% in Burkina Faso, Cote d'Ivoire, and Mozambique.

### 3.2 Inequities in Nutrition Counseling Coverage

**Figure 1:**
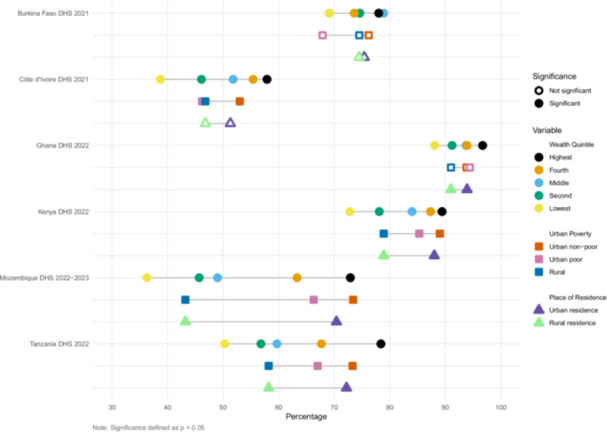
Intervention coverage of counseling about maternal diet during antenatal care visit.

**Figure 2:**
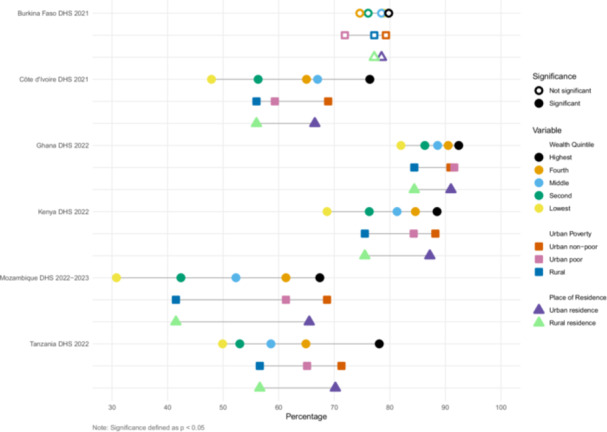
Intervention coverage of counseling about breastfeeding during antenatal care visit.

**Figure 3:**
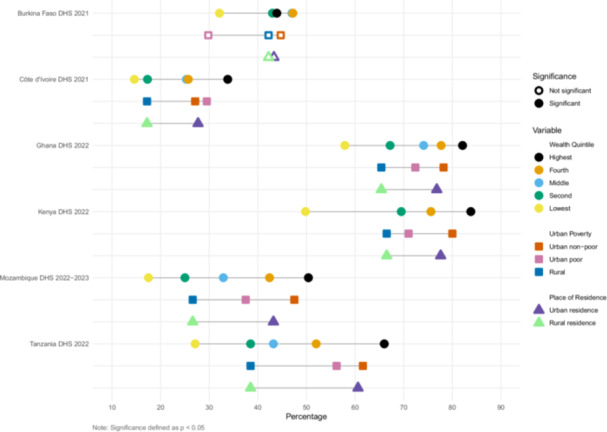
Intervention coverage of counseling about and observation of breastfeeding during postnatal care visit. Note: This is a composite indicator of these two interventions.

**Figure 4:**
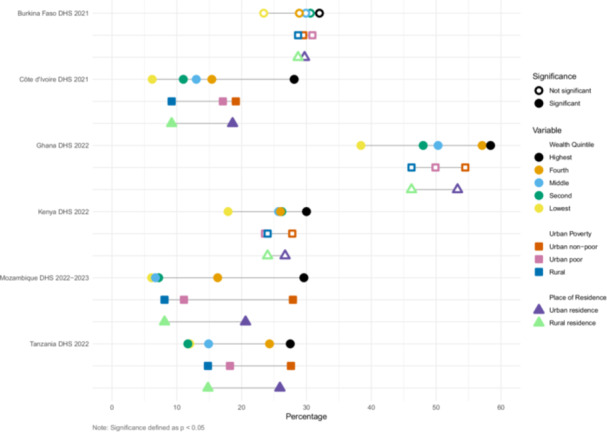
Intervention coverage of counseling about infant and young child feeding counseling for mothers of 6–23‐month‐old children in the last 6 months.

We estimated inequities in coverage between wealth quintile, urban poverty, and urban/rural residence (Figures 1, 2, 3, 4). The widest equity gap for both ANC counseling indicators was 36.6 percentage points between the highest and lowest wealth quintiles in Mozambique (*p* value < 0.01), compared to the smallest equity gap of 10.4 percentage points in Ghana (*p* value < 0.01) (Figures 1 and 2). There was a similar magnitude gap in counseling and observation of breastfeeding during PNC between the highest and lowest wealth quintile, 36 and 36.9 percentage points in Tanzania and Mozambique, respectively (p values < 0.01 and < 0.01, respectively) (Figure 3). Equity gaps were smaller for IYCF counseling (Figure 4).

Across the six countries and along the continuum of care, wealthier and urban mothers reported receiving nutrition counseling more often than poor urban and rural mothers (Supporting Information S1: Tables 2–19). Urban nonpoor mothers generally reported the highest coverage, followed by urban poor then rural mothers, but there were some notable exceptions. In Burkina Faso, 68% of urban poor mothers reported receiving information about maternal diet and 72% about breastfeeding during ANC, less than that of rural mothers, who reported 75% and 77% coverage, respectively, though these within country differences did not reach significance at *p* < 0.05. In Côte d'Ivoire, urban poor mothers reported the highest coverage of observation of breastfeeding (36%), relative to urban nonpoor (31%) and rural mothers (19%) (*p* value < 0.01).

### 3.3 Utilizing the New GM and Acute Malnutrition Screening Coverage Data to Better Understand Inequities in Kenya

#### 3.3.1 Delivery of GM and Screening for Acute Malnutrition in Kenya

GM is the regular measurement of infant and young children's weight and health/length compared to a growth standard. Screening for wasting or SAM typically measures MUAC or a weight‐for‐height z‐score compared to a threshold value for diagnosis and referral. These interventions serve as a regular contact point between caregivers and health‐care services and is intended to catch growth faltering early (GM) and high risk of mortality (SAM) (Ashworth et al. 2008; Mangasaryan et al. 2011; World Health Organization & United Nations Children's Fund (‎‎UNICEF)‎‎ 2009).

In Kenya, like many LMICs, GM is offered as regular screening through government health facilities and child welfare clinics, while Integrated Management of Childhood Illness (IMCI) is commonly community‐based, employing Community Health Volunteers (CHVs) who screen for SAM (Kenyan Ministry of Health 2020; KNBS and ICF 2023). Screening for SAM is more common in arid, semi‐arid areas and rural counties of Kenya, where its long‐term rates are persistently higher due to a combination environmental, economic, and health system factors (Kenya National Bureau of Statistics, Ministry of Health/Kenya, National AIDS Control Council/Kenya, Kenya Medical Research Institute, Population, N. C. f., & Development/Kenya 2015; KNBS and ICF 2023).

#### 3.3.2 Inequities in Coverage of GM and Screening for Acute Malnutrition

**Figure 5:**
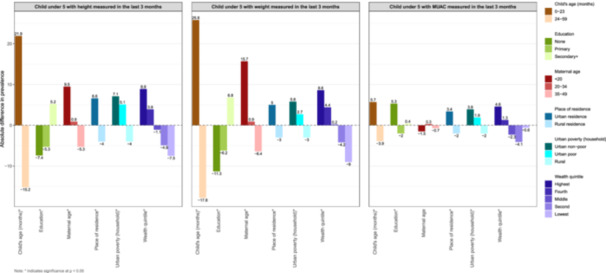
Absolute percentage point differences in growth monitoring and acute malnutrition coverage indicators by equity characteristics, relative to the national estimate. Note: Each bar represents the difference between the subgroup estimate (e.g., by wealth, education, or residence) and the national average for the indicator. Positive values indicate higher coverage than the national average; negative values indicate lower coverage.

Nationally, 45% of mothers reported that their child had their weight measured, compared to 38% for height and 16% for MUAC. The widest disparity in coverage for all three interventions was observed by county; measurement of both height and weight had a 71‐percentage point difference between the highest and lowest counties (Supporting Information S1: Table 13). For the other key stratifiers, there were similar trends and magnitudes in the coverage of weight and height. There was a 44‐percentage point difference for weight (*p* value < 0.01) and 37 percentage point difference for height/length (*p* value < 0.01) between those under 2 years of age and those 2 years and older (Figure 5). Maternal age, education, and wealth quintile also showed significant differences, with more than a 10‐percentage point gap in coverage for both height/length and weight measurement, favoring older, educated, and wealthy mothers. For MUAC, however, less educated mothers reported higher coverage than those with more education and mothers in the poorest wealth quintile had slightly higher coverage than those in the middle and fourth quartiles (Figure 5).

#### 3.3.3 Interpretation and Application

Subnational disparities in coverage in Kenya continue, with ongoing approaches to reduce these. In 2013, Kenya transitioned to a decentralized system of health service delivery, transferring management from the national government to the 47 county governments. A key objective of decentralization was to reduce regional disparities in access to care, particularly the use of preventive services (Ilinca et al. 2019; Masaba et al. 2020). Devolution was intended to allocate resources more equitably to counties to improve local service delivery. The wide disparities in anthropometry, however, reveal ongoing gaps and point to a continued need to implement contextually‐relevant models and interventions that address local population health needs.

More recently, health sector reform in Kenya has focused universal free access to public primary care services in an effort to achieve UHC (Ministry of Health 2020). Related reforms include the systematic integration of CHVs into primary health care, which has the potential to reduce inequities through focused targeting of subgroups with lower coverage of specific interventions. For example, the smaller inequities in MUAC by maternal education and wealth point to focused and appropriate targeting of children most at risk of SAM by CHVs and could be expanded within counties based on the largest disparities.

The second largest disparity in coverage of anthropometric assessment was by child age. Coverage dropped in those over 2 years of age, following well‐baby and Essential Program on Immunization (EPI) visits. This is not unique to Kenya, yet highlights, again, the importance of subgroup targeting combined with focused outreach and community‐based GM to improve coverage of nutrition interventions for children 2–5 years of age, especially to detect wasting and SAM (World Health Organization & United Nations Children's Fund (UNICEF) 2023). In four Kenyan counties, “family‐led MUAC” screening programs, where mothers, caregivers, and family members are trained to measure and classify MUAC at home, have been piloted by multiple nongovernmental organizations (Grant et al. 2018). In one randomized trial in Kenya, this program increased coverage of screening for SAM and reduced wasting by 37% (Tickell et al. 2023).

## 4 Discussion

The call for a “nutrition data revolution” in 2014 began a decade‐long process to align and advocate for collection and reporting of high‐impact nutrition interventions (International Food Policy Research Institute (IFPRI) 2014). The expanded nutrition content in DHS‐8 reflects the impact of these efforts and provides new information to shape nutrition policy and programs at subnational, national, and global levels.

Across all six countries, nutrition intervention coverage during pregnancy, birth, and PNC was higher than during infancy and childhood. This trend mirrors that of maternal, newborn, and child health (MNCH) intervention coverage along the continuum of care. Strong advocacy efforts for maternal and neonatal survival led many countries to successfully implement national policies that provide free health services to women and children in the first 1,000 days (Requejo et al. 2020; Smith and Shiffman 2016; Starrs 2006; World Health Organization 2014). The delivery of nutrition interventions during these life stages has benefited from increased access to their health delivery platforms. Yet, opportunity and equity gaps persist. For example, there were large opportunity gaps between birthing in a facility and early initiation of breastfeeding and skin‐to‐skin contact. There were also meaningful gaps between childhood vaccination coverage and IYCF counseling and anthropometry measurement. These gaps highlight missed opportunities for the delivery if nutrition interventions and can also be used to diagnose implementation bottlenecks and devise appropriate solutions. In Burkina Faso, identification of an opportunity gap during pregnancy led to a successful effort to intensify counseling of consumption of IFA and breastfeeding practices (Kim et al. 2023). Closing gaps for interventions that require significant provider or recipient behavior change will require rigorous and sustained efforts (Requejo et al. 2020).

Through our analysis of opportunity gaps, we note that some commonly used coverage indicators for health and nutrition service delivery have not kept pace with changes in intervention guidance. While consistency of an indicator definition across time is important for trend analysis, there can be a trade off in consistency of the indicator with current WHO recommendations. For example, ANC continues to be frequently reported as attendance at 4 or more visits, which was based on the previous Focused ANC model (UNDO/UNFPA/WHO/World Bank 2002). However, the 2016 Positive Pregnancy Experience now recommends at least 8 visits at specific times during pregnancy (World Health Organization 2016). Likewise, iron supplementation is now recommended daily during pregnancy beginning in the first trimester in most contexts, yet taking iron‐containing supplements for 90 days, rather than the estimated 180 days, continues to be more commonly reported (Lopez de Romaña et al. 2023; World Health Organization 2024a). The motivation for selection of these indicators might vary by context and objective and its justification should be transparent. Additionally, multiple micronutrient supplementation (MMS) has recently been recommended to replace IFA under certain circumstances, and is starting to be rolled out, with implementation research ongoing (World Health Organization 2020). MMS process and outcome indicators should be considered in future data collection efforts to reflect this (DataDENT 2025; Walton et al. 2023).

Literature on urban poverty, diets, and nutrition outcomes has highlighted the influence of the physical and social environment on healthy diets (Vilar‐Compte et al. 2021). Inequities in the coverage of nutrition interventions by maternal education, wealth, and residence were similar to MNCH interventions (Victora et al. 2019; Victora et al. 2003). Disparities within urban populations were observed for several coverage indicators across all countries, providing a more granular understanding of who is being reached, with actionable findings of where to intensify efforts based on relevant barriers.

There is an ongoing need to align and triangulate indicators across national household surveys, health facility assessments, and routine HMIS platforms to strengthen information systems for nutrition. Each data source provides unique types of information. While national surveys provided population‐based data on who is and is not being reached, useful to guide strategy, HMIS data are produced more frequently and at lower administrative areas within countries, which can facilitate ongoing program management and course correction (United States Agency for International Development (USAID) 2018). However, few countries have subnational HMIS nutrition data on IYCF, child growth, and supplementation (Mallick et al. 2020).

We presented new opportunities for analysis of the enhanced DHS‐8 intervention coverage data, yet there are limitations of: a) the indicators themselves, and b) our analysis. First, the inclusion of four new and revised indicators about MIYCN counseling highlight their importance as part of nutrition services. However, the DHS survey questions that are used as the basis for the counseling indicators are phrased to ask the respondent if a provider “talked to about” a topic. These indicators could therefore overestimate true nutritional counseling by including health education or promotion, while failing to capture its multidimensional nature.

Second, our analysis focused on intervention coverage and stopped short of “effective coverage,” a concept that has been proposed to include health care system readiness, quality of care, and intervention adherence to better estimate outcome‐adjusted coverage (Marsh et al. 2020; Shengelia et al. 2005). This and related comprehensive measures are valuable and require further research about how to integrate data sources, linkages between the steps in the cascade of care, and feasibility of measurement (Marsh et al. 2020).

Third, the identification of the appropriate health service delivery platforms for nutrition interventions provided through ANC, delivery, and PNC proved easier than for interventions among infants and young children. We selected basic vaccinations between 12 and 35 months as a proxy for utilization of preventive health services in this life stage, yet the time frame for this indicator does not fully overlap with the nutrition coverage indicators targeted to infants and young children, making the opportunity gap at this age harder to interpret.

Finally, several of the nutrition intervention coverage questions depend on respondent recall and have not been validated against a gold standard measure of intervention receipt; this is true of many of the questions included in the DHS. Recent validation studies have shown that maternal recall about the quantity of iron‐containing supplements consumed during pregnancy at 6 months postpartum is poor (Bryce et al. 2022); other studies have shown relatively low validity of questions about specific MIYCN counseling messages received in previous months, yet high validity for breastfeeding counseling (Ashok et al. 2022; McKay et al. 2024; Thorne‐Lyman et al. 2022). Ongoing investment is needed in formative research to improve comprehension of questions, methods for coverage measurement, the ideal timing of recall periods, and potential triangulation with HMIS data and other representative facility‐based surveys such as the DHS Service Provision Assessments (SPA) and the WHO Harmonized Health Facility Assessments (HHFA).

In March of 2025, the DHS program ended abruptly and unexpectedly. The use of DHS data had increased exponentially over the past two decades and its sudden stoppage harms data systems and blunts data‐driven decision‐making globally (Locks et al. 2025). The technical and scientific advancements achieved through DHS Program's data collection and the consultative processes to improve successive surveys was a significant accomplishment. The learnings and progress made by the DHS can continue to be valuable to practitioners, policy makers, and researchers for future survey design and data collection efforts.

## 5 Conclusion

The expansion of the DHS nutrition intervention coverage data was a positive step in the multi‐year effort to coordinate and align multiple nutrition indicators to be more useful for decision‐making and advocacy. Our analyses revealed gaps in coverage of nutrition services along the continuum of care, most notably in childhood. Ultimately the delivery and equitable coverage of nutrition interventions depend on the strength of a health system's building blocks and its ability to promote access, quality, and optimal use of resources. These data help identify where and for whom barriers to mainstreaming nutrition in reproductive, maternal, newborn, and child health services could be better understood to deliver and sustain nutrition interventions. Despite the end of the DHS Program, these strategies and lessons can be incorporated into future efforts.

## Author Contributions

R.K. conceived of the research idea, E.P. led the drafting the manuscript, and S.Z. performed statistical analyses and created the figures. All authors contributed to the data interpretation and critical review of the manuscript. All authors provided final approval for publication and agree to be accountable for aspects of the work.

## Ethics Statement

The authors have nothing to report.

## Conflicts of Interest

The authors declare no conflicts of interest.

## Supporting information


**Supporting Table S1. Related to Table 1:** Summary of Demographic and Health Surveys (Round 8) from 6 Countries. **Supporting Table S2. Related to Table 2:** Health Delivery Platform and Nutrition Intervention Coverage during Antenatal Care by Background Characteristic ‐ Burkina Faso. **Supporting Table S3. Related to Table 3:** Health Delivery Platform and Nutrition Intervention Coverage during Birth and Postnatal Care by Background Characteristic ‐ Burkina Faso. **Supporting Table S4. Related to Table 4:** Health Delivery Platform and Nutrition Intervention Coverage during Infant and Young Childhood by Background Characteristic ‐ Burkina Faso. **Supporting Table S5. Related to Table 5:** Health Delivery Platform and Nutrition Intervention Coverage during Antenatal Care by Background Characteristic ‐ Cote d'Ivoire. **Supporting Table S6. Related to Table 6:** Health Delivery Platform and Nutrition Intervention Coverage during Birth and Postnatal Care by Background Characteristic – Cote d'Ivoire. **Supporting Table S7. Related to Table 7:** Health Delivery Platform and Nutrition Intervention Coverage during Infant and Young Childhood by Background Characteristic – Cote d'Ivoire. **Supporting Table S8. Related to Table 8:** Health Delivery Platform and Nutrition Intervention Coverage during Antenatal Care by Background Characteristic ‐ Ghana. **Supporting Table S9. Related to Table 9:** Health Delivery Platform and Nutrition Intervention Coverage during Birth and Postnatal Care by Background Characteristic ‐ Ghana. **Supporting Table S10. Related to Table 10:** Health Delivery Platform and Nutrition Intervention Coverage during Infant and Young Childhood by Background Characteristic ‐ Ghana. **Supporting Table S11. Related to Table 11:** Health Delivery Platform and Nutrition Intervention Coverage during Antenatal Care by Background Characteristic ‐ Kenya. **Supporting Table S12. Related to Table 12:** Health Delivery Platform and Nutrition Intervention Coverage during Birth and Postnatal Care by Background Characteristic — Kenya. **Supporting Table S13. Related to Table 13:** Health Delivery Platform and Nutrition Intervention Coverage during Infant and Young Childhood by Background Characteristic ‐ Kenya. **Supporting Table S14. Related to Table 14:** Health Delivery Platform and Nutrition Intervention Coverage during Antenatal Care by Background Characteristic ‐ Mozambique. **Supporting Table S15. Related to Table 15:** Health Delivery Platform and Nutrition Intervention Coverage during Birth and Postnatal Care by Background Characteristic ‐ Mozambique. **Supporting Table S16. Related to Table 16:** Health Delivery Platform and Nutrition Intervention Coverage during Infant and Young Childhood by Background Characteristic —Mozambique. **Supporting Table S17. Related to Table 17:** Health Delivery Platform and Nutrition Intervention Coverage during Antenatal Care by Background Characteristic ‐ Tanzania. **Supporting Table S18. Related to Table 18:** Health Delivery Platform and Nutrition Intervention Coverage during Birth and Postnatal Care by Background Characteristic ‐ Tanzania. **Supporting Table S19. Related to Table 19:** Health Delivery Platform and Nutrition Intervention Coverage during Infant and Young Childhood by Background Characteristic ‐ Tanzania.

## Data Availability

The data that support the findings of this study are available in The DHS Program Data at https://dhsprogram.com/Data/. These data were derived from the following resources available in the public domain: ‐ The DHS Program, https://dhsprogram.com.
