# Supplementary material for: Nutrition Intervention Coverage and Inequities Along the Continuum of Care: Results From the Eighth Demographic and Health Survey in Six Sub‐Saharan African Countries
Source: Matern Child Nutr. 2025 Aug 29;22(1):e70085. doi: 10.1111/mcn.70085 (PMC12893515; doi:10.1111/mcn.70085)
Supplement: Supplementary file 1 — Supporting Table S1. Related to Table 1: Summary of Demographic and Health Surveys (Round 8) from 6 Countries. Supporting Table S2. Related to Table 2: Health Delivery Platform and Nutrition Intervention Coverage during Antenatal Care by Background Characteristic ‐ Burkina Faso. Supporting Table S3. Related to Table 3: Health Delivery Platform and Nutrition Intervention Coverage during Birth and Postnatal Care by Background Characteristic ‐ Burkina Faso. Supporting Table S4. Related to Table 4: Health Delivery Platform and Nutrition Intervention Coverage during Infant and Young Childhood by Background Characteristic ‐ Burkina Faso. Supporting Table S5. Related to Table 5: Health Delivery Platform and Nutrition Intervention Coverage during Antenatal Care by Background Characteristic ‐ Cote d'Ivoire. Supporting Table S6. Related to Table 6: Health Delivery Platform and Nutrition Intervention Coverage during Birth and Postnatal Care by Background Characteristic – Cote d'Ivoire. Supporting Table S7. Related to Table 7: Health Delivery Platform and Nutrition Intervention Coverage during Infant and Young Childhood by Background Characteristic – Cote d'Ivoire. Supporting Table S8. Related to Table 8: Health Delivery Platform and Nutrition Intervention Coverage during Antenatal Care by Background Characteristic ‐ Ghana. Supporting Table S9. Related to Table 9: Health Delivery Platform and Nutrition Intervention Coverage during Birth and Postnatal Care by Background Characteristic ‐ Ghana. Supporting Table S10. Related to Table 10: Health Delivery Platform and Nutrition Intervention Coverage during Infant and Young Childhood by Background Characteristic ‐ Ghana. Supporting Table S11. Related to Table 11: Health Delivery Platform and Nutrition Intervention Coverage during Antenatal Care by Background Characteristic ‐ Kenya. Supporting Table S12. Related to Table 12: Health Delivery Platform and Nutrition Intervention Coverage during Birth and Postnatal Care by Background Char [file MCN-22-e70085-s001.docx]

Supplementary Table 1: Summary of Demographic and Health Surveys (Round 8) from 6 Countries

| **Country** | **Survey year** | **Number of women aged 15–49 years with a live birth in the past 2 years** | **Number of children aged <5** |
| --- | --- | --- | --- |
| Burkina Faso | 2021 | 4,684 | 11,855 |
| Cote d'Ivoire | 2021 | 3,858 | 9,156 |
| Ghana | 2022 | 3,491 | 8,315 |
| Kenya | 2022 | 6,847 | 16,883 |
| Mozambique | 2022–2023 | 3,822 | 9,396 |
| Tanzania | 2022 | 4,335 | 10,497 |

Supplementary Table 2: Health Delivery Platform and Nutrition Intervention Coverage during Antenatal Care by Background Characteristic - Burkina Faso

| **Pregnancy/antenatal care (ANC)** | | | | | | | | | | | | | | | | | | | | |
| --- | --- | --- | --- | --- | --- | --- | --- | --- | --- | --- | --- | --- | --- | --- | --- | --- | --- | --- | --- | --- |
|  | **Health delivery platform: Attended 4+ ANC visits** | | **Health delivery platform: Attended 8+ ANC visits** | | **Took iron-containing supplements for any number of days during most recent pregnancy** | | **Took iron-containing supplements for 90+ days during most recent pregnancy** | | **Took iron-containing supplements for 180+ days during most recent pregnancy** | | **Took intestinal parasite drugs during most recent pregnancy** | | **Counseled about maternal diet during ANC visit** | | **Counseled about breastfeeding during ANC visit** | | **Source of iron-containing supplements: public sector** | | **Source of iron-containing supplements: private sector + other** | |
|  | **%** | **N** | **%** | **N** | **%** | **N** | **%** | **N** | **%** | **N** | **%** | **N** | **%** | **N** | **%** | **N** | **%** | **N** | **%** | **N** |
| **Total** | 72.1 | 4,684 | 0.9 | 4,684 | 90.4 | 4,684 | 53.9 | 4,684 | 13.0 | 4,684 | 64.1 | 4,684 | 74.7 | 4,684 | 84.9 | 4,684 | 96.8 | 4,496 | 3.8 | 4,496 |
| **Background characteristic** |  |  |  |  |  |  |  |  |  |  |  |  |  |  |  |  |  |  |  |  |
| **Sex of child** | p=0.292 | | p=0.583 | | p=0.940 | | p=0.982 | | p=0.261 | | p=0.895 | | p=0.060 | | p=0.275 | | p=0.792 | | p=0.435 | |
| Male | 71.3 | 2,384 | * | 2,384 | 90.4 | 2,384 | 53.9 | 2,384 | 12.4 | 2,384 | 64.0 | 2,384 | 73.5 | 2,384 | 76.8 | 2,384 | 96.7 | 2,293 | 4.1 | 2,293 |
| Female | 72.8 | 2,299 | * | 2,299 | 90.4 | 2,299 | 53.9 | 2,299 | 13.6 | 2,299 | 64.2 | 2,299 | 76.0 | 2,299 | 78.3 | 2,299 | 96.9 | 2,203 | 3.6 | 2,203 |
| **Maternal age** | p=0.007 | | p=0.205 | | p=0.132 | | p=0.042 | | p=0.064 | | p=0.032 | | p=0.193 | | p=0.678 | | p=0.035 | | p=0.010 | |
| <20 | 65.2 | 456 | * | 456 | 89.3 | 456 | 47.9 | 456 | 11.0 | 456 | 58.5 | 456 | 71.5 | 456 | 79.0 | 456 | 99.4 | 430 | * | 430 |
| 20–34 | 72.7 | 3,269 | 0.9 | 3,269 | 90.0 | 3,269 | 54.0 | 3,269 | 12.5 | 3,269 | 64.2 | 3,269 | 74.7 | 3,269 | 77.2 | 3,269 | 96.6 | 3,139 | 4.2 | 3,139 |
| 35–49 | 73.2 | 958 | * | 958 | 92.3 | 958 | 56.4 | 958 | 15.5 | 958 | 66.6 | 958 | 76.5 | 958 | 78.2 | 958 | 96.3 | 927 | 4.0 | 927 |
| **Mother's education** | p=0.043 | | p=0.002 | | p=0.629 | | p=0.000 | | p=0.000 | | p=0.432 | | p=0.512 | | p=0.215 | | p=0.000 | | p=0.000 | |
| None | 70.7 | 3,127 | * | 3,127 | 90.7 | 3,127 | 50.7 | 3,127 | 10.8 | 3,127 | 63.4 | 3,127 | 74.2 | 3,127 | 77.2 | 3,127 | 98.9 | 3,001 | 1.5 | 3,001 |
| Primary | 74.3 | 672 | * | 672 | 90.3 | 672 | 60.4 | 672 | 13.5 | 672 | 66.0 | 672 | 76.7 | 672 | 80.6 | 672 | 95.4 | 646 | 5.6 | 646 |
| Secondary+ | 75.0 | 884 | * | 884 | 89.4 | 884 | 60.2 | 884 | 20.4 | 884 | 65.2 | 884 | 75.2 | 884 | 76.5 | 884 | 90.3 | 850 | 10.7 | 850 |
| **Place of residence** | p=0.162 | | p=0.120 | | p=0.829 | | p=0.000 | | p=0.000 | | p=0.108 | | p=0.700 | | p=0.518 | | p=0.000 | | p=0.000 | |
| Urban | 74.1 | 1,184 | * | 1,184 | 90.2 | 1,184 | 62.7 | 1,184 | 20.8 | 1,184 | 67.1 | 1,184 | 75.4 | 1,184 | 78.5 | 1,184 | 89.4 | 1,135 | 11.8 | 1,135 |
| Rural | 71.4 | 3,499 | * | 3,499 | 90.5 | 3,499 | 50.9 | 3,499 | 10.3 | 3,499 | 63.1 | 3,499 | 74.5 | 3,499 | 77.2 | 3,499 | 99.3 | 3,361 | 1.2 | 3,361 |
| **Urban poverty household** | p=0.128 | | p=0.296 | | p=0.549 | | p=0.000 | | p=0.000 | | p=0.076 | | p=0.335 | | p=0.282 | | p=0.000 | | p=0.000 | |
| Urban non-poor | 74.0 | 1,070 | * | 1,070 | 90.6 | 1,070 | 63.9 | 1,070 | 21.6 | 1,070 | 68.0 | 1,070 | 76.2 | 1,070 | 79.3 | 1,070 | 88.8 | 1,026 | 12.4 | 1,026 |
| Urban poor | 79.9 | 114 | * | 114 | 86.6 | 114 | 51.1 | 114 | 13.2 | 114 | 59.2 | 114 | 67.9 | 114 | 71.9 | 114 | 94.5 | 108 | * | 108 |
| Rural | 71.4 | 3,499 | 0.7 | 3,499 | 90.5 | 3,499 | 50.9 | 3,499 | 10.3 | 3,499 | 63.1 | 3,499 | 74.5 | 3,499 | 77.2 | 3,499 | 99.3 | 3,361 | 1.2 | 3,361 |
| **Wealth quintile** | p=0.002 | | p=0.173 | | p=0.564 | | p=0.000 | |  |  | p=0.049 | | p=0.007 | | p=0.231 | | p=0.000 | | p=0.000 | |
| Lowest | 67.1 | 990 | * | 990 | 90.2 | 990 | 41.3 | 990 | 9.6 | 990 | 62.4 | 990 | 69.1 | 990 | 78.9 | 990 | 99.7 | 946 | * | 946 |
| Second | 71.8 | 991 | * | 991 | 88.9 | 991 | 49.9 | 991 | 9.6 | 991 | 62.3 | 991 | 74.6 | 991 | 76.1 | 991 | 99.5 | 947 | * | 947 |
| Middle | 71.2 | 958 | * | 958 | 91.1 | 958 | 55.8 | 958 | 12.5 | 958 | 62.9 | 958 | 78.9 | 958 | 78.5 | 958 | 99.5 | 926 | * | 926 |
| Fourth | 73.4 | 908 | * | 908 | 90.6 | 908 | 59.8 | 908 | 12.6 | 908 | 64.0 | 908 | 73.6 | 908 | 74.6 | 908 | 97.7 | 871 | 3.5 | 871 |
| Highest | 77.7 | 837 | * | 837 | 91.5 | 837 | 65.0 | 837 | 22.0 | 837 | 70.0 | 837 | 78.0 | 837 | 79.8 | 837 | 86.0 | 805 | 14.8 | 805 |
| **Region** | p=0.000 | | p=0.373 | | p=0.000 | | p=0.000 | | p=0.000 | | p=0.023 | | p=0.000 | | p=0.000 | | p=0.000 | | p=0.000 | |
| Boucle du Mouhoun | 64.0 | 451 | * | 451 | 80.3 | 451 | 58.3 | 451 | 21.0 | 451 | 63.4 | 451 | 80.2 | 451 | 78.8 | 451 | 99.3 | 422 | * | 422 |
| Cascades | 75.2 | 137 | * | 137 | 89.3 | 137 | 59.3 | 137 | 24.0 | 137 | 73.2 | 137 | 83.4 | 137 | 85.4 | 137 | 98.2 | 136 | * | 136 |
| Centre | 67.9 | 713 | * | 713 | 88.9 | 713 | 66.8 | 713 | 17.5 | 713 | 64.1 | 713 | 72.9 | 713 | 74.8 | 713 | 86.3 | 674 | 14.2 | 674 |
| Centre Est | 78.1 | 522 | * | 522 | 97.7 | 522 | 60.9 | 522 | 9.2 | 522 | 65.6 | 522 | 75.2 | 522 | 75.1 | 522 | 99.0 | 514 | * | 514 |
| Centre Nord | 77.2 | 462 | * | 462 | 85.1 | 462 | 33.5 | 462 | * | 462 | 57.3 | 462 | 85.5 | 462 | 82.8 | 462 | 99.9 | 444 | * | 444 |
| Centre Ouest | 70.0 | 464 | * | 464 | 87.7 | 464 | 58.4 | 464 | 7.9 | 464 | 65.2 | 464 | 75.1 | 464 | 73.2 | 464 | 99.1 | 441 | * | 441 |
| Centre Sud | 75.8 | 180 | * | 180 | 98.9 | 180 | 77.7 | 180 | 11.9 | 180 | 68.0 | 180 | 50.8 | 180 | 53.8 | 180 | 98.1 | 178 | * | 178 |
| Est | 74.3 | 387 | * | 387 | 94.5 | 387 | 25.1 | 387 | 8.6 | 387 | 51.9 | 387 | 52.1 | 387 | 77.2 | 387 | 99.5 | 369 | * | 369 |
| Hauts-Bassins | 76.7 | 450 | * | 450 | 89.5 | 450 | 49.2 | 450 | 17.9 | 450 | 66.0 | 450 | 65.7 | 450 | 69.6 | 450 | 95.1 | 425 | * | 425 |
| Nord | 81.3 | 281 | * | 281 | 89.6 | 281 | 58.3 | 281 | 24.4 | 281 | 69.7 | 281 | 82.4 | 281 | 83.9 | 281 | 97.8 | 275 | * | 275 |
| Plateau central | 76.0 | 313 | * | 313 | 97.9 | 313 | 61.3 | 313 | 6.6 | 313 | 65.0 | 313 | 86.1 | 313 | 90.0 | 313 | 99.3 | 311 | * | 311 |
| Sahel | 33.3 | 149 | * | 149 | 94.0 | 149 | 26.6 | 149 | * | 149 | 67.4 | 149 | 95.0 | 149 | 91.7 | 149 | 98.7 | 142 | * | 142 |
| Sud-Ouest | 71.4 | 175 | * | 175 | 92.2 | 175 | 61.3 | 175 | 12.2 | 175 | 74.8 | 175 | 78.6 | 175 | 85.2 | 175 | 99.3 | 164 | * | 164 |
| An asterisk indicates that the figure is based on 25 or fewer unweighted cases and has been suppressed.  Light gray shading indicates p<0.05.  Dark gray shading indicates p<0.001. | | | | | | | | | | | | | | | | | | | | |

*Continued*

Supplementary Table 3: Health Delivery Platform and Nutrition Intervention Coverage during Birth and Postnatal Care by Background Characteristic - Burkina Faso

|  | **Birth** | | | | | | **Postnatal care (PNC)** | | | | | | | | |
| --- | --- | --- | --- | --- | --- | --- | --- | --- | --- | --- | --- | --- | --- | --- | --- |
|  | **Health delivery platform: Live births delivered in a health facility** | | **Skin-to-skin contact immediately after birth** | | **Started breastfeeding within one hour of birth** | | | **Health delivery platform: PNC check within two days for newborn** | | **Weighed during newborn PNC check** | | **Counseled about breastfeeding during newborn PNC check** | | **Observed breastfeeding during newborn PNC check** | |
|  | **%** | **N** | **%** | **N** | **%** | **N** | | **%** | **N** | **%** | **N** | **%** | **N** | **%** | **N** |
| **Total** | 94.2 | 4,830 | 85.0 | 4,684 | 60.3 | 4,830 | | 78.3 | 4,684 | 84.9 | 4,684 | 48.8 | 4,684 | 45.0 | 4,684 |
| **Background characteristic** |  |  |  |  |  |  | |  |  |  |  |  |  |  |  |
| **Sex of child** | p=0.017 | | p=0.552 | | p=0.996 | | | p=0.146 | | p=0.235 | | p=0.458 | | p=0.344 | |
| Male | 93.5 | 2,468 | 84.7 | 2,384 | 60.3 | 2,468 | | 77.4 | 2,384 | 84.2 | 2,384 | 48.2 | 2,384 | 44.2 | 2,384 |
| Female | 94.9 | 2,362 | 85.4 | 2,299 | 60.3 | 2,362 | | 79.2 | 2,299 | 85.6 | 2,299 | 49.4 | 2,299 | 45.8 | 2,299 |
| **Maternal age** | p=0.367 | | p=0.141 | | p=0.856 | | | p=0.525 | | p=0.758 | | p=0.370 | | p=0.677 | |
| <20 | 95.5 | 462 | 85.5 | 456 | 61.6 | 462 | | 80.2 | 456 | 83.9 | 456 | 45.4 | 456 | 42.8 | 456 |
| 20–34 | 94.2 | 3,386 | 85.6 | 3,269 | 60.2 | 3,386 | | 77.9 | 3,269 | 85.1 | 3,269 | 49.1 | 3,269 | 45.3 | 3,269 |
| 35–49 | 93.5 | 982 | 82.9 | 958 | 60.0 | 982 | | 78.8 | 958 | 84.4 | 958 | 49.4 | 958 | 45.0 | 958 |
| **Mother's education** | p=0.000 | | p=0.186 | | p=0.180 | | | p=0.009 | | p=0.000 | | p=0.003 | | p=0.001 | |
| None | 92.2 | 3,225 | 84.3 | 3,127 | 61.4 | 3,225 | | 76.8 | 3,127 | 82.4 | 3,127 | 47.0 | 3,127 | 42.8 | 3,127 |
| Primary | 97.8 | 691 | 87.2 | 672 | 58.7 | 691 | | 81.6 | 672 | 87.1 | 672 | 50.2 | 672 | 48.0 | 672 |
| Secondary+ | 98.4 | 913 | 86.0 | 884 | 57.7 | 913 | | 81.1 | 884 | 91.8 | 884 | 54.1 | 884 | 50.4 | 884 |
| **Place of residence** | p=0.000 | | p=0.154 | | p=0.536 | | | p=0.882 | | p=0.000 | | p=0.072 | | p=0.354 | |
| Urban | 98.2 | 1,214 | 86.7 | 1,184 | 59.2 | 1,214 | | 78.5 | 1,184 | 90.3 | 1,184 | 52.0 | 1,184 | 46.8 | 1,184 |
| Rural | 92.9 | 3,616 | 84.5 | 3,499 | 60.7 | 3,616 | | 78.2 | 3,499 | 83.0 | 3,499 | 47.7 | 3,499 | 44.4 | 3,499 |
| **Urban poverty household** | p=0.000 | | p=0.246 | | p=0.690 | | | p=0.428 | | p=0.000 | | p=0.063 | | p=0.092 | |
| Urban non-poor | 98.4 | 1,096 | 86.6 | 1,070 | 59.0 | 1,096 | | 79.1 | 1,070 | 90.4 | 1,070 | 52.7 | 1,070 | 48.1 | 1,070 |
| Urban poor | 96.2 | 118 | 87.4 | 114 | 60.9 | 118 | | 72.7 | 114 | 88.8 | 114 | 45.4 | 114 | 35.0 | 114 |
| Rural | 92.9 | 3,616 | 84.5 | 3,499 | 60.7 | 3,616 | | 78.2 | 3,499 | 83.0 | 3,499 | 47.7 | 3,499 | 44.4 | 3,499 |
| **Wealth quintile** | p=0.000 | | p=0.000 | | p=0.521 | | | p=0.000 | | p=0.000 | | p=0.000 | | p=0.000 | |
| Lowest | 85.3 | 1,021 | 79.9 | 990 | 61.3 | 1,021 | | 72.1 | 990 | 75.2 | 990 | 37.3 | 990 | 33.8 | 990 |
| Second | 94.2 | 1,023 | 84.6 | 991 | 60.0 | 1,023 | | 79.6 | 991 | 84.2 | 991 | 49.7 | 991 | 44.9 | 991 |
| Middle | 95.9 | 987 | 87.3 | 958 | 62.9 | 987 | | 79.8 | 958 | 86.0 | 958 | 52.9 | 958 | 49.0 | 958 |
| Fourth | 98.5 | 940 | 89.5 | 908 | 58.7 | 940 | | 83.1 | 908 | 87.9 | 908 | 52.7 | 908 | 51.0 | 908 |
| Highest | 98.2 | 858 | 84.2 | 837 | 58.3 | 858 | | 77.1 | 837 | 92.5 | 837 | 52.5 | 837 | 47.3 | 837 |
| **Region** | p=0.000 | | p=0.000 | | p=0.000 | | | p=0.000 | | p=0.000 | | p=0.000 | | p=0.000 | |
| Boucle du Mouhoun | 96.6 | 466 | 94.6 | 451 | 70.1 | 466 | | 85.1 | 451 | 86.6 | 451 | 27.3 | 451 | 20.3 | 451 |
| Cascades | 98.1 | 144 | 82.5 | 137 | 81.5 | 144 | | 72.8 | 137 | 84.3 | 137 | 54.3 | 137 | 55.4 | 137 |
| Centre | 99.1 | 732 | 87.4 | 713 | 56.0 | 732 | | 82.4 | 713 | 92.1 | 713 | 53.6 | 713 | 49.9 | 713 |
| Centre Est | 98.6 | 538 | 93.7 | 522 | 55.9 | 538 | | 86.7 | 522 | 87.1 | 522 | 69.8 | 522 | 63.8 | 522 |
| Centre Nord | 93.5 | 474 | 80.2 | 462 | 44.9 | 474 | | 85.6 | 462 | 83.9 | 462 | 51.0 | 462 | 48.1 | 462 |
| Centre Ouest | 94.6 | 473 | 75.3 | 464 | 53.8 | 473 | | 70.2 | 464 | 78.9 | 464 | 55.0 | 464 | 48.5 | 464 |
| Centre Sud | 96.9 | 184 | 82.8 | 180 | 44.8 | 184 | | 78.4 | 180 | 88.4 | 180 | 23.7 | 180 | 20.5 | 180 |
| Est | 88.6 | 397 | 83.4 | 387 | 67.3 | 397 | | 71.2 | 387 | 80.4 | 387 | 20.9 | 387 | 18.8 | 387 |
| Hauts-Bassins | 95.6 | 471 | 85.0 | 450 | 68.8 | 471 | | 67.7 | 450 | 87.4 | 450 | 51.9 | 450 | 48.0 | 450 |
| Nord | 96.8 | 292 | 88.9 | 281 | 67.6 | 292 | | 79.7 | 281 | 90.6 | 281 | 52.2 | 281 | 51.8 | 281 |
| Plateau central | 97.2 | 323 | 89.9 | 313 | 73.6 | 323 | | 92.0 | 313 | 91.3 | 313 | 63.9 | 313 | 62.5 | 313 |
| Sahel | 44.2 | 154 | 52.9 | 149 | 34.6 | 154 | | 39.4 | 149 | 39.6 | 149 | 22.3 | 149 | 19.9 | 149 |
| Sud-Ouest | 91.7 | 180 | 84.0 | 175 | 70.6 | 180 | | 74.4 | 175 | 81.0 | 175 | 64.9 | 175 | 61.7 | 175 |

Light gray shading indicates p<0.05.

Dark gray shading indicates p<0.001.

*Continued…*

Supplementary Table 4: Health Delivery Platform and Nutrition Intervention Coverage during Infant and Young Childhood by Background Characteristic - Burkina Faso

| **Infancy and childhood** | | | | | | | | | | | | | | | | | |
| --- | --- | --- | --- | --- | --- | --- | --- | --- | --- | --- | --- | --- | --- | --- | --- | --- | --- |
|  | **Health Delivery platform: All basic vaccinations^ according to either source^&^  (12–35 mos)** | | **Mothers of children age 6–23 mos who received IYCF counseling in last 6 mos** | | **Child under 5 with weight measured in the last 3 mos** | | **Child under 5 with height measured in the last 3 mos** | | **Child under 5 with MUAC measured in the last 3 mos** | | **Children age 6–59 mos given iron containing supplements** | | **Children age 6–59 mos given Vit. A supplements** | | **Children age 12–59 mos given deworming medication** | | |
|  | **%** | **N** | **%** | **N** | **%** | **N** | **%** | **N** | **%** | **N** | **%** | **N** | **%** | **N** | **%** | **N** | |
| **Total** | 70.7 | 4,287 | 28.9 |  | 39.4 | 11,855 | 37.6 | 11,855 | 34.5 | 11,855 | 33.1 | 10,576 | 37.0 | 10,576 | 31.4 | 9,456 | |
| **Background characteristic** |  |  |  |  |  |  |  |  |  |  |  |  |  |  |  |  | |
| **Sex of child** | p=0.719 | | p=0.782 | | p=0.546 | | p=0.464 | | p=0.452 | | p=0.611 | | p=0.350 | | p=0.312 | | |
| Male | 71.0 | 2,191 | 29.2 | 1,694 | 39.7 | 5,999 | 38.0 | 5,999 | 34.8 | 5,999 | 32.8 | 5,370 | 37.5 | 5,370 | 31.9 | 4,804 | |
| Female | 70.4 | 2,097 | 28.7 | 1,610 | 39.1 | 5,856 | 37.3 | 5,856 | 34.1 | 5,856 | 33.4 | 5,206 | 36.5 | 5,206 | 30.9 | 4,652 | |
| **Child’s age in months** |  |  |  |  | p=0.000 | | p=0.000 | | p=0.000 | |  |  |  |  |  |  | |
| 0–23 |  |  |  |  | 62.8 | 4,698 | 62.6 | 4,698 | 58.1 | 4,698 |  |  |  |  |  |  | |
| 24–59 |  |  |  |  | 24.0 | 7,157 | 21.3 | 7,157 | 19.0 | 7,157 |  |  |  |  |  |  | |
| **Maternal age** | p=0.086 | | p=0.349 | | p=0.000 | | p=0.000 | | p=0.000 | | p=0.001 | | p=0.026 | | p=0.001 | | |
| <20 | 64.5 | 254 | 25.4 | 299 | 58.8 | 581 | 59.3 | 581 | 54.3 | 581 | 42.5 | 439 | 38.0 | 439 | 30.7 | 320 | |
| 20–34 | 70.6 | 3,054 | 29.7 | 2,343 | 39.5 | 8,213 | 37.6 | 8,213 | 34.4 | 8,213 | 32.2 | 7,338 | 36.0 | 7,338 | 30.1 | 6,536 | |
| 35–49 | 72.7 | 980 | 27.9 | 662 | 35.3 | 3,060 | 33.7 | 3,060 | 30.9 | 3,060 | 33.9 | 2,800 | 39.6 | 2,800 | 34.9 | 2,600 | |
| **Mother’s education** | p=0.813 | | p=0.014 | | p=0.000 | | p=0.001 | | p=0.001 | | p=0.017 | | p=0.103 | | p=0.083 | | |
| None | 70.4 | 2,921 | 27.5 | 2,217 | 37.6 | 8,271 | 36.4 | 8,271 | 33.2 | 8,271 | 32.6 | 7,435 | 36.4 | 7,435 | 30.6 | 6,723 | |
| Primary | 71.9 | 604 | 28.4 | 456 | 42.1 | 1,592 | 39.3 | 1,592 | 37.7 | 1,592 | 31.1 | 1,387 | 36.6 | 1,387 | 32.0 | 1,216 | |
| Secondary+ | 70.9 | 762 | 34.1 | 631 | 44.4 | 1,992 | 41.4 | 1,992 | 37.1 | 1,992 | 36.8 | 1,754 | 40.1 | 1,754 | 34.5 | 1,517 | |
| **Place of residence** | p=0.030 | | p=0.653 | | p=0.346 | | p=0.486 | | p=0.958 | | p=0.664 | | p=0.988 | | p=0.117 | | |
| Urban | 67.1 | 1,089 | 29.7 | 852 | 40.5 | 2,945 | 38.5 | 2,945 | 34.5 | 2,945 | 32.5 | 2,637 | 37.1 | 2,637 | 34.0 | 2,341 | |
| Rural | 71.9 | 3,199 | 28.7 | 2,452 | 39.0 | 8,910 | 37.4 | 8,910 | 34.4 | 8,910 | 33.3 | 7,938 | 37.0 | 7,938 | 30.6 | 7,115 | |
| **Urban poverty household** | p=0.048 | | p=0.832 | | p=0.184 | | p=0.191 | | p=0.244 | | p=0.416 | | p=0.203 | | p=0.094 | | |
| Urban non-poor | 67.3 | 969 | 29.5 | 757 | 41.1 | 2,655 | 39.2 | 2,655 | 35.2 | 2,655 | 32.4 | 2,363 | 38.0 | 2,363 | 34.7 | 2,101 | |
| Urban poor | 65.3 | 120 | 30.9 | 95 | 34.7 | 290 | 32.4 | 290 | 28.4 | 290 | 57.5 | 274 | 28.8 | 274 | 27.9 | 240 | |
| Rural | 71.9 | 3,199 | 28.7 | 2,452 | 39.0 | 8,910 | 37.4 | 8,910 | 34.4 | 8,910 | 33.3 | 7,938 | 37.0 | 7,938 | 30.6 | 7,115 | |
| **Wealth quintile** | p=0.367 | | p=0.067 | | p=0.001 | | p=0.029 | | p=0.077 | | p=0.008 | | p=0.142 | | p=0.047 | | |
| Lowest | 68.7 | 901 | 23.4 | 660 | 34.8 | 2,489 | 2,489 | 2,489 | 31.6 | 2,489 | 34.8 | 2,184 | 34.5 | 2,184 | 31.6 | 1,959 | |
| Second | 70.3 | 897 | 30.6 | 709 | 38.9 | 2,447 | 2,447 | 2,447 | 35.2 | 2,447 | 33.4 | 2,186 | 38.8 | 2,186 | 31.2 | 1,957 | |
| Middle | 71.4 | 871 | 29.9 | 693 | 40.1 | 2,463 | 2,463 | 2,463 | 34.4 | 2,463 | 34.3 | 2,222 | 37.4 | 2,222 | 29.0 | 1,973 | |
| Fourth | 73.9 | 861 | 28.9 | 641 | 41.0 | 2,383 | 2,383 | 2,383 | 34.8 | 2,383 | 28.1 | 2,127 | 35.3 | 2,127 | 30.3 | 1,916 | |
| Highest | 69.2 | 757 | 32.0 | 601 | 42.8 | 2,073 | 2,073 | 2,073 | 36.7 | 2,073 | 35.0 | 1,856 | 39.4 | 1,856 | 35.8 | 1,651 | |
| **Region** | p=0.000 | | p=0.000 | | p=0.000 | | p=0.000 | | p=0.000 | | p=0.000 | | p=0.000 | | p=0.000 | | |
| Boucle du Mouhoun | 71.3 | 406 | 25.7 | 322 | 31.4 | 1,169 | 31.0 | 1,169 | 31.2 | 1,169 | 38.3 | 1,044 | 23.8 | 1,044 | 23.9 | 930 | |
| Cascades | 81.2 | 118 | 39.8 | 92 | 50.0 | 346 | 48.9 | 346 | 42.5 | 346 | 34.0 | 308 | 52.6 | 308 | 47.0 | 271 | |
| Centre | 71.4 | 651 | 32.1 | 480 | 38.3 | 1,852 | 35.7 | 1,852 | 30.6 | 1,852 | 27.2 | 1,625 | 28.7 | 1,625 | 27.5 | 1,461 | |
| Centre Est | 76.1 | 445 | 30.7 | 379 | 48.3 | 1,288 | 42.3 | 1,288 | 38.3 | 1,288 | 29.1 | 1,154 | 31.4 | 1,154 | 31.6 | 1,019 | |
| Centre Nord | 65.5 | 436 | 26.0 | 354 | 31.4 | 1,171 | 30.3 | 1,171 | 28.1 | 1,171 | 35.8 | 1,078 | 37.2 | 1,078 | 20.1 | 967 | |
| Centre Ouest | 70.3 | 431 | 34.8 | 317 | 43.7 | 1,225 | 42.8 | 1,225 | 36.3 | 1,225 | 37.0 | 1,089 | 46.1 | 1,089 | 32.1 | 986 | |
| Centre Sud | 84.3 | 183 | * | 128 | 36.1 | 471 | 32.9 | 471 | 30.4 | 471 | 16.3 | 423 | 58.2 | 423 | 49.2 | 382 | |
| Est | 62.1 | 357 | 10.2 | 268 | 34.9 | 954 | 34.0 | 954 | 28.0 | 954 | 47.8 | 845 | 34.1 | 845 | 30.9 | 758 | |
| Hauts-Bassins | 69.2 | 445 | 23.7 | 324 | 35.5 | 1,141 | 34.3 | 1,141 | 33.1 | 1,141 | 30.3 | 1,024 | 37.5 | 1,024 | 32.2 | 919 | |
| Nord | 69.0 | 249 | 37.3 | 199 | 53.9 | 736 | 54.2 | 736 | 48.8 | 736 | 48.2 | 659 | 39.3 | 659 | 34.4 | 592 | |
| Plateau central | 82.3 | 290 | 43.2 | 229 | 39.7 | 728 | 39.0 | 728 | 37.9 | 728 | 14.3 | 648 | 40.2 | 648 | 28.4 | 574 | |
| Sahel | 44.7 | 125 | 37.4 | 93 | 35.1 | 349 | 35.3 | 349 | 31.9 | 349 | 22.8 | 299 | 18.4 | 299 | 29.9 | 265 | |
| Sud-Ouest | 69.0 | 153 | 29.5 | 119 | 41.7 | 426 | 39.6 | 426 | 48.4 | 426 | 44.2 | 379 | 74.7 | 379 | 67.2 | 332 | |
| An asterisk indicates that the figure is based on 25 or fewer unweighted cases and has been suppressed. | | | | | | | | | | | | | | | | | |
| ^All basic antigen vaccines: 1 dose BCG, 3 doses Polio (OPV/IPV), 3 doses DPT, 1 dose measles (MR) | | | | | | | | | | | | | | | | |  |
| & either source - vaccination card or mother's report (crude coverage)  Light gray shading indicates p<0.05.  Dark gray shading indicates p<0.001. | | | | | | | | | | | | | | | | |  |

Supplementary Table 5: Health Delivery Platform and Nutrition Intervention Coverage during Antenatal Care by Background Characteristic - Cote d’Ivoire

|  | **Pregnancy/antenatal care (ANC)** | | | | | | | | | | | | | | | | | | | |
| --- | --- | --- | --- | --- | --- | --- | --- | --- | --- | --- | --- | --- | --- | --- | --- | --- | --- | --- | --- | --- |
|  | **Health delivery platform: Attended 4+ ANC visits** | | **Health delivery platform: Attended 8+ ANC visits** | | **Took iron-containing supplements for any number of days during most recent pregnancy** | | **Took iron-containing supplements for 90+ days during most recent pregnancy** | | **Took iron-containing supplements for 180+ days during most recent pregnancy** | | **Took intestinal parasite drugs during most recent pregnancy** | | **Counseled about maternal diet during ANC visit** | | **Counseled about breastfeeding during ANC visit** | | **Source of iron-containing supplements: public sector** | | **Source of iron-containing supplements: private sector + other** | |
|  | **%** | **N** | **%** | **N** | **%** | **N** | **%** | **N** | **%** | **N** | **%** | **N** | **%** | **N** | **%** | **N** | **%** | **N** | **%** | **N** |
| **Total** | 56.3 | 3,858 | 3.7 | 3,858 | 84.8 | 3,858 | 28.9 | 3,858 | 9.3 | 3,858 | 51.4 | 3,858 | 49.0 | 3,858 | 61.2 | 3,858 | 58.9 | 3,351 | 44.3 | 3,351 |
| **Background characteristic** |  |  |  |  |  |  |  |  |  |  |  |  |  |  |  |  |  |  |  |  |
| **Sex of child** | p=0.096 | | p=0.369 | | p=0.484 | | p=0.088 | | p=0.360 | | p=0.666 | | p=0.093 | | p=0.991 | | p=0.254 | | p=0.332 | |
| Male | 58.0 | 1,931 | 4.2 | 1,931 | 84.3 | 1,931 | 30.5 | 1,931 | 9.9 | 1,931 | 50.9 | 1,931 | 47.4 | 1,931 | 61.2 | 1,931 | 57.7 | 1,660 | 45.3 | 1,660 |
| Female | 54.6 | 1,927 | 3.3 | 1,927 | 85.2 | 1,927 | 27.2 | 1,927 | 8.8 | 1,927 | 51.8 | 1,927 | 50.7 | 1,927 | 61.2 | 1,927 | 60.1 | 1,691 | 43.3 | 1,691 |
| **Maternal age** | p=0.064 | | p=0.608 | | p=0.640 | | p=0.684 | | p=0.121 | | p=0.303 | | p=0.001 | | p=0.336 | | p=0.165 | | p=0.155 | |
| <20 | 48.8 | 374 | * | 374 | 86.8 | 374 | 27.4 | 374 | 5.7 | 374 | 47.4 | 374 | 38.5 | 374 | 57.4 | 374 | 65.3 | 335 | 37.9 | 335 |
| 20–34 | 57.4 | 2,748 | 4.0 | 2,748 | 84.5 | 2,748 | 29.4 | 2,748 | 9.6 | 2,748 | 51.3 | 2,748 | 49.5 | 2,748 | 61.3 | 2,748 | 58.4 | 2,384 | 44.8 | 2,384 |
| 35–49 | 56.1 | 736 | * | 736 | 84.7 | 736 | 27.7 | 736 | 10.1 | 736 | 53.7 | 736 | 52.6 | 736 | 62.9 | 736 | 57.5 | 632 | 45.8 | 632 |
| **Mother's education** | p=0.000 | | p=0.000 | | p=0.000 | | p=0.000 | | p=0.002 | | p=0.000 | | p=0.000 | | p=0.020 | | p=0.000 | | p=0.000 | |
| None | 48.2 | 2,298 | 2.2 | 2,298 | 80.6 | 2,298 | 24.9 | 2,298 | 7.8 | 2,298 | 45.7 | 2,298 | 45.4 | 2,298 | 58.2 | 2,298 | 65.4 | 1,901 | 37.4 | 1,901 |
| Primary | 62.7 | 722 | 4.5 | 722 | 89.4 | 722 | 31.3 | 722 | 9.2 | 722 | 54.5 | 722 | 52.4 | 722 | 65.6 | 722 | 58.7 | 657 | 45.9 | 657 |
| Secondary+ | 73.0 | 839 | 7.1 | 839 | 92.0 | 839 | 37.6 | 839 | 13.6 | 839 | 64.2 | 839 | 56.0 | 839 | 65.7 | 839 | 43.6 | 793 | 59.5 | 793 |
| **Place of residence** | p=0.000 | | p=0.000 | | p=0.035 | | p=0.018 | | p=0.000 | | p=0.000 | | p=0.079 | | p=0.000 | | p=0.000 | | p=0.000 | |
| Urban | 68.1 | 1,917 | 5.7 | 1,917 | 86.5 | 1,917 | 31.7 | 1,917 | 12.1 | 1,917 | 58.1 | 1,917 | 51.3 | 1,917 | 66.5 | 1,917 | 44.2 | 1,723 | 59.1 | 1,723 |
| Rural | 44.6 | 1,941 | 1.8 | 1,941 | 83.0 | 1,941 | 26.1 | 1,941 | 6.7 | 1,941 | 44.7 | 1,941 | 46.8 | 1,941 | 56.0 | 1,941 | 74.5 | 1,628 | 28.6 | 1,628 |
| **Urban poverty household** | p=0.000 | | p=0.000 | | p=0.025 | | p=0.043 | | p=0.000 | | p=0.000 | | p=0.068 | | p=0.000 | | p=0.000 | | p=0.000 | |
| Urban non-poor | 71.8 | 1,429 | 6.8 | 1,429 | 88.1 | 1,429 | 30.9 | 1,429 | 11.7 | 1,429 | 60.6 | 1,429 | 53.0 | 1,429 | 68.9 | 1,429 | 44.2 | 1,306 | 58.6 | 1,306 |
| Urban poor | 57.3 | 488 | * | 488 | 81.8 | 488 | 33.9 | 488 | 13.2 | 488 | 50.8 | 488 | 46.2 | 488 | 59.3 | 488 | 44.1 | 417 | 60.5 | 417 |
| Rural | 44.6 | 1,941 | 1.8 | 1,941 | 83.0 | 1,941 | 26.1 | 1,941 | 6.7 | 1,941 | 44.7 | 1,941 | 46.8 | 1,941 | 56.0 | 1,941 | 74.5 | 1,628 | 28.6 | 1,628 |
| **Wealth quintile** | p=0.000 | | p=0.000 | | p=0.000 | | p=0.000 | | p=0.000 | | p=0.000 | | p=0.000 | | p=0.000 | | p=0.000 | | p=0.000 | |
| Lowest | 36.3 | 926 | * | 926 | 78.6 | 926 | 19.1 | 926 | 5.3 | 926 | 38.4 | 926 | 38.7 | 926 | 47.9 | 926 | 74.1 | 734 | 28.3 | 734 |
| Second | 47.5 | 840 | * | 840 | 82.7 | 840 | 30.8 | 840 | 9.4 | 840 | 45.9 | 840 | 46.1 | 840 | 56.3 | 840 | 70.9 | 703 | 32.0 | 703 |
| Middle | 54.0 | 773 | * | 773 | 86.0 | 773 | 30.7 | 773 | 7.6 | 773 | 54.0 | 773 | 51.8 | 773 | 67.0 | 773 | 60.9 | 681 | 43.4 | 681 |
| Fourth | 69.2 | 702 | 5.7 | 702 | 88.1 | 702 | 33.1 | 702 | 9.4 | 702 | 59.9 | 702 | 55.4 | 702 | 65.0 | 702 | 47.1 | 649 | 57.0 | 649 |
| Highest | 86.5 | 617 | 10.8 | 617 | 91.5 | 617 | 33.6 | 617 | 17.4 | 617 | 65.3 | 617 | 57.9 | 617 | 76.4 | 617 | 36.1 | 584 | 65.9 | 584 |
| **Region** | p=0.000 | | p=0.000 | | p=0.000 | | p=0.000 | | p=0.000 | | p=0.000 | | p=0.000 | | p=0.000 | | p=0.000 | | p=0.000 | |
| Abidjan | 78.6 | 797 | * | 797 | 86.6 | 797 | 25.7 | 797 | 12.2 | 797 | 59.5 | 797 | 50.4 | 797 | 66.8 | 797 | 34.7 | 740 | 66.8 | 740 |
| Yamoussoukro | 61.1 | 49 | * | 49 | 95.1 | 49 | 57.5 | 49 | 19.9 | 49 | 72.5 | 49 | 52.7 | 49 | 69.0 | 49 | 52.6 | 47 | 51.0 | 47 |
| Bas Sassandra | 46.3 | 373 | * | 373 | 91.7 | 373 | 26.4 | 373 | 8.3 | 373 | 56.2 | 373 | 54.3 | 373 | 64.1 | 373 | 63.7 | 344 | 40.1 | 344 |
| Comoe | 68.1 | 166 | * | 166 | 93.5 | 166 | 36.3 | 166 | * | 166 | 61.2 | 166 | 71.1 | 166 | 74.7 | 166 | 64.4 | 155 | 39.4 | 155 |
| Denguele | 34.7 | 67 | * | 67 | 71.5 | 67 | 17.0 | 67 | * | 67 | 36.7 | 67 | 34.5 | 67 | 44.4 | 67 | 67.4 | 49 | 36.6 | 49 |
| Goh-Djiboua | 53.3 | 261 | * | 261 | 91.6 | 261 | 37.6 | 261 | * | 261 | 50.6 | 261 | 67.7 | 261 | 72.8 | 261 | 71.9 | 241 | 32.7 | 241 |
| Lacs | 53.3 | 178 | * | 178 | 93.7 | 178 | 40.1 | 178 | 12.3 | 178 | 63.6 | 178 | 49.9 | 178 | 61.4 | 178 | 67.0 | 169 | 37.5 | 169 |
| Lagunes | 59.4 | 227 | * | 227 | 86.2 | 227 | 33.3 | 227 | * | 227 | 56 | 227 | 51.4 | 227 | 59.6 | 227 | 63.1 | 198 | 41.0 | 198 |
| Montagnes | 44.2 | 397 | * | 397 | 89.0 | 397 | 31.0 | 397 | 7.9 | 397 | 46.2 | 397 | 41.5 | 397 | 55.1 | 397 | 67.9 | 354 | 38.9 | 354 |
| Sassandra-Marahoue | 52.3 | 425 | * | 425 | 75.6 | 425 | 15.8 | 425 | * | 425 | 35.6 | 425 | 37.4 | 425 | 51.2 | 425 | 73.7 | 322 | 28.3 | 322 |
| Savanes | 47.8 | 303 | * | 303 | 75.3 | 303 | 31.1 | 303 | 16.9 | 303 | 50.6 | 303 | 45.8 | 303 | 60.7 | 303 | 56.6 | 238 | 45.2 | 238 |
| Vallee du Bandama | 57.3 | 234 | * | 234 | 81.2 | 234 | 36.9 | 234 | 11.9 | 234 | 52.4 | 234 | 43.6 | 234 | 62.9 | 234 | 49.0 | 197 | 53.7 | 197 |
| Woroba | 36.0 | 191 | * | 191 | 76.3 | 191 | 15.0 | 191 | * | 191 | 29.4 | 191 | 30.4 | 191 | 41.4 | 191 | 74.5 | 147 | 27.9 | 147 |
| Zanzan | 48.8 | 192 | * | 192 | 78.1 | 192 | 34.0 | 192 | * | 192 | 50.7 | 192 | 60.9 | 192 | 63.9 | 192 | 73.6 | 151 | 28.3 | 151 |
| An asterisk indicates that the figure is based on 25 or fewer unweighted cases and has been suppressed.  Light gray shading indicates p<0.05.  Dark gray shading indicates p<0.001. | | | | | | | | | | | | | | | | | | | | |

*Continued…*

Supplementary Table 6: Health Delivery Platform and Nutrition Intervention Coverage during Birth and Postnatal Care by Background Characteristic – Cote d’Ivoire

|  | **Birth** | | | | | | **Postnatal care (PNC)** | | | | | | | |
| --- | --- | --- | --- | --- | --- | --- | --- | --- | --- | --- | --- | --- | --- | --- |
|  | **Health delivery platform: Live births delivered in a health facility** | | **Skin-to-skin contact immediately after birth** | | **Started breastfeeding within one hour of birth** | | **Health delivery platform: PNC check within two days for newborn** | | **Weighed during newborn PNC check** | | **Counseled about breastfeeding during newborn PNC check** | | **Observed breastfeeding during newborn PNC check** | |
|  | **%** | **N** | **%** | **N** | **%** | **N** | **%** | **N** | **%** | **N** | **%** | **N** | **%** | **N** |
| **Total** | 80.9 | 3,967 | 37.8 | 3,858 | 42.3 | 3,967 | 72.2 | 3,858 | 80.0 | 3,858 | 31.6 | 3,858 | 25.8 | 3,858 |
| **Background characteristic** |  |  |  |  |  |  |  |  |  |  |  |  |  |  |
| **Sex of child** | p=0.058 | | p=0.442 | | p=0.732 | | p=0.783 | | p=0.046 | | p=0.999 | | p=0.538 | |
| Male | 82.3 | 1,988 | 38.6 | 1,931 | 42.0 | 1,988 | 72.4 | 1,931 | 81.5 | 1,931 | 31.6 | 1,931 | 25.2 | 1,931 |
| Female | 79.5 | 1,979 | 36.9 | 1,927 | 42.6 | 1,979 | 71.9 | 1,927 | 78.6 | 1,927 | 31.6 | 1,927 | 26.4 | 1,927 |
| **Maternal age** | p=0.120 | | p=0.912 | | p=0.043 | | p=0.847 | | p=0.299 | | p=0.133 | | p=0.950 | |
| <20 | 78.2 | 377 | 38.6 | 374 | 36.4 | 377 | 71.1 | 374 | 77.4 | 374 | 27.8 | 374 | 26.3 | 374 |
| 20–34 | 81.8 | 2,825 | 37.5 | 2,748 | 42.1 | 2,825 | 72.5 | 2,748 | 79.9 | 2,748 | 31.3 | 2,748 | 25.8 | 2,748 |
| 35–49 | 78.7 | 766 | 38.3 | 736 | 45.9 | 766 | 71.6 | 736 | 81.8 | 736 | 34.5 | 736 | 25.3 | 736 |
| **Mother's education** | p=0.000 | | p=0.000 | | p=0.001 | | p=0.863 | | p=0.000 | | p=0.000 | | p=0.001 | |
| None | 76.4 | 2,369 | 34.1 | 2,298 | 45.3 | 2,369 | 71.9 | 2,298 | 75.5 | 2,298 | 27.4 | 2,298 | 22.7 | 2,298 |
| Primary | 82.7 | 743 | 39.0 | 722 | 39.6 | 743 | 73.2 | 722 | 83.3 | 722 | 32.8 | 722 | 28.6 | 722 |
| Secondary+ | 91.7 | 856 | 46.7 | 839 | 36.4 | 856 | 71.9 | 839 | 89.8 | 839 | 42.2 | 839 | 31.8 | 839 |
| **Place of residence** | p=0.000 | | p=0.000 | | p=0.659 | | p=0.218 | | p=0.000 | | p=0.000 | | p=0.000 | |
| Urban | 90.6 | 1,965 | 42.2 | 1,917 | 41.8 | 1,965 | 73.5 | 1,917 | 89.9 | 1,917 | 39.7 | 1,917 | 32.3 | 1,917 |
| Rural | 71.3 | 2,002 | 33.4 | 1,941 | 42.8 | 2,002 | 70.8 | 1,941 | 70.3 | 1,941 | 23.6 | 1,941 | 19.3 | 1,941 |
| **Urban poverty household** | p=0.000 | | p=0.006 | | p=0.799 | | p=0.505 | | p=0.000 | | p=0.000 | | p=0.000 | |
| Urban non-poor | 92.7 | 1,472 | 43.1 | 1,429 | 42.3 | 1,472 | 73.5 | 1,429 | 91.6 | 1,429 | 40.8 | 1,429 | 31.0 | 1,429 |
| Urban poor | 84.3 | 493 | 39.7 | 488 | 40.4 | 493 | 73.3 | 488 | 85.0 | 488 | 36.7 | 488 | 35.9 | 488 |
| Rural | 71.3 | 2,002 | 33.4 | 1,941 | 42.8 | 2,002 | 70.8 | 1,941 | 70.3 | 1,941 | 23.6 | 1,941 | 19.3 | 1,941 |
| **Wealth quintile** | p=0.000 | | p=0.000 | | p=0.538 | | p=0.033 | | p=0.000 | | p=0.000 | | p=0.000 | |
| Lowest | 62.0 | 958 | 30.2 | 926 | 43.5 | 958 | 67.5 | 926 | 61.2 | 926 | 20.8 | 926 | 16.9 | 926 |
| Second | 74.0 | 862 | 36.9 | 840 | 41.5 | 862 | 71.5 | 840 | 75.0 | 840 | 24.0 | 840 | 20.7 | 840 |
| Middle | 86.6 | 798 | 34.1 | 773 | 45.1 | 798 | 74.9 | 773 | 85.7 | 773 | 32.9 | 773 | 27.7 | 773 |
| Fourth | 94.9 | 719 | 42.7 | 702 | 41.1 | 719 | 77.0 | 702 | 91.2 | 702 | 37.5 | 702 | 30.7 | 702 |
| Highest | 95.8 | 631 | 49.3 | 617 | 39.6 | 631 | 71.1 | 617 | 95.5 | 617 | 49.8 | 617 | 38.0 | 617 |
| **Region** | p=0.000 | | p=0.000 | | p=0.000 | | p=0.000 | | p=0.000 | | p=0.000 | | p=0.000 | |
| Abidjan | 94.5 | 807 | 48.5 | 797 | 44.2 | 807 | 69.2 | 797 | 93.1 | 797 | 47.3 | 797 | 37.6 | 797 |
| Yamoussoukro | 85.6 | 51 | 40.2 | 49 | 40.9 | 51 | 68.1 | 49 | 91.1 | 49 | 34.1 | 49 | 22.1 | 49 |
| Bas Sassandra | 79.1 | 383 | 41.5 | 373 | 46.2 | 383 | 77.9 | 373 | 74.4 | 373 | 26.1 | 373 | 20.1 | 373 |
| Comoe | 93.9 | 172 | 42.8 | 166 | 35.9 | 172 | 78.6 | 166 | 94.4 | 166 | 45.6 | 166 | 41.7 | 166 |
| Denguele | 63.4 | 70 | 37.8 | 67 | 45.1 | 70 | 71.1 | 67 | 65.3 | 67 | 24.6 | 67 | 20.3 | 67 |
| Goh-Djiboua | 81.8 | 271 | 48.1 | 261 | 45.4 | 271 | 81.7 | 261 | 82.6 | 261 | 28.4 | 261 | 27.9 | 261 |
| Lacs | 76.1 | 185 | 38.4 | 178 | 52.3 | 185 | 60.4 | 178 | 82.9 | 178 | 36.2 | 178 | 29.1 | 178 |
| Lagunes | 81.6 | 232 | 26.2 | 227 | 44.5 | 232 | 78.0 | 227 | 86.7 | 227 | 32.2 | 227 | 26.4 | 227 |
| Montagnes | 69.2 | 409 | 34.4 | 397 | 28.9 | 409 | 79.9 | 397 | 66.9 | 397 | 21.6 | 397 | 17.5 | 397 |
| Sassandra-Marahoue | 72.6 | 438 | 27.8 | 425 | 43.0 | 438 | 64.9 | 425 | 73.3 | 425 | 17.6 | 425 | 12.0 | 425 |
| Savanes | 82.9 | 314 | 29.8 | 303 | 54.7 | 314 | 74.3 | 303 | 78.8 | 303 | 29.6 | 303 | 22.4 | 303 |
| Vallee du Bandama | 86.1 | 239 | 36.0 | 234 | 40.7 | 239 | 65.8 | 234 | 82.1 | 234 | 34.8 | 234 | 30.0 | 234 |
| Woroba | 60.3 | 196 | 20.0 | 191 | 34.1 | 196 | 64.3 | 191 | 60.2 | 191 | 10.8 | 191 | 8.9 | 191 |
| Zanzan | 78.5 | 198 | 41.6 | 192 | 32.9 | 198 | 72.3 | 192 | 74.2 | 192 | 37.5 | 192 | 34.7 | 192 |

Light gray shading indicates p<0.05.

Dark gray shading indicates p<0.001.

*Continued…*

Supplementary Table 7: Health Delivery Platform and Nutrition Intervention Coverage during Infant and Young Childhood by Background Characteristic – Cote d’Ivoire

|  | **Infancy and childhood** | | | | | | | | | | | | | | | |  |
| --- | --- | --- | --- | --- | --- | --- | --- | --- | --- | --- | --- | --- | --- | --- | --- | --- | --- |
|  | **Health Delivery platform: All basic vaccinations^ according to either source^&^ (12–35 mos)** | | **Mothers of children age 6–23 mos who received IYCF counseling in last 6 mos** | | **Child under 5 with weight measured in the last 3 mos** | | **Child under 5 with height measured in the last 3 mos** | | **Child under 5 with MUAC measured in the last 3 mons** | | **Children age 6–59 mos given iron containing supplements** | | **Children age 6–59 mos given Vit. A supplements** | | **Children age 12–59 mos given deworming medication** | |  |
|  | **%** | **N** | **%** | **N** | **%** | **N** | **%** | **N** | **%** | **N** | **%** | **N** | **%** | **N** | **%** | **N** |  |
| **Total** | 34.0 | 3,546 | 13.8 | 2,644 | 27.3 | 9,156 | 25.0 | 9,156 | 20.8 | 9,156 | 48.9 | 8,157 | 46.0 | 8,157 | 45.8 | 7,206 |  |
| **Background characteristic** |  |  |  |  |  |  |  |  |  |  |  |  |  |  |  |  |  |
| **Sex of child** | p=0.860 | | p=0.315 | | p=0.934 | | p=0.861 | | p=0.604 | | p=0.486 | | p=0.398 | | p=0.736 | |  |
| Male | 34.2 | 1,810 | 13.0 | 1,337 | 27.4 | 4,620 | 24.9 | 4,620 | 21.1 | 4,620 | 49.3 | 4,138 | 46.7 | 4,138 | 45.5 | 3,687 |  |
| Female | 33.8 | 1,736 | 14.7 | 1,307 | 27.3 | 4,536 | 25.1 | 4,536 | 20.5 | 4,536 | 48.4 | 4,019 | 45.4 | 4,019 | 46.0 | 3,519 |  |
| **Child's age in months** |  |  |  |  | p=0.000 | | p=0.000 | | p=0.000 | |  |  |  |  |  |  |  |
| 0–23 |  |  |  |  | 39.1 | 3,769 | 36.6 | 3,769 | 31.5 | 3,769 |  |  |  |  |  |  |  |
| 24–59 |  |  |  |  | 19.1 | 5,387 | 16.9 | 5,387 | 13.4 | 5,387 |  |  |  |  |  |  |  |
| **Maternal age** | p=0.001 | | p=0.374 | | p=0.132 | | p=0.273 | | p=0.087 | | p=0.317 | | p=0.130 | | p=0.009 | |  |
| <20 | 24.8 | 237 | * | 205 | 30.3 | 555 | 27.6 | 555 | 23.9 | 555 | 46.2 | 424 | 40.6 | 424 | 33.6 | 331 |  |
| 20–34 | 33.2 | 2,510 | 13.9 | 1,900 | 27.8 | 6,409 | 25.2 | 6,409 | 21.2 | 6,409 | 49.6 | 5,705 | 45.9 | 5,705 | 46.3 | 5,015 |  |
| 35–49 | 39.3 | 799 | 14.9 | 538 | 25.3 | 2,192 | 23.7 | 2,192 | 19.0 | 2,192 | 47.4 | 2,028 | 47.5 | 2,028 | 46.5 | 1,860 |  |
| **Mother's education** | p=0.000 | | p=0.001 | | p=0.000 | | p=0.000 | | p=0.000 | | p=0.000 | | p=0.000 | | p=0.000 | |  |
| None | 30.1 | 2,131 | 11.0 | 1,620 | 22.0 | 5,710 | 20.5 | 5,710 | 17.1 | 5,710 | 42.8 | 5,156 | 42.1 | 5,156 | 37.5 | 4,559 |  |
| Primary | 39.7 | 670 | 16.5 | 480 | 31.2 | 1,702 | 29.1 | 1,702 | 24.6 | 1,702 | 54.7 | 1,492 | 49.1 | 1,492 | 55.6 | 1,323 |  |
| Secondary+ | 40.2 | 745 | 20.0 | 544 | 41.0 | 1,743 | 35.9 | 1,743 | 29.4 | 1,743 | 63.8 | 1,509 | 56.4 | 1,509 | 64.5 | 1,324 |  |
| **Place of residence** | p=0.007 | | p=0.000 | | p=0.000 | | p=0.000 | | p=0.000 | | p=0.000 | | p=0.003 | | p=0.000 | |  |
| Urban | 31.0 | 1,845 | 18.6 | 1,300 | 33.0 | 4,532 | 29.9 | 4,532 | 24.4 | 4,532 | 54.7 | 4,038 | 49.9 | 4,038 | 55.2 | 3,581 |  |
| Rural | 37.4 | 1,701 | 9.2 | 1,344 | 21.7 | 4,624 | 20.2 | 4,624 | 17.3 | 4,624 | 43.1 | 4,120 | 42.3 | 4,120 | 36.5 | 3,625 |  |
| **Urban poverty household** | p=0.005 | | p=0.000 | | p=0.000 | | p=0.000 | | p=0.000 | | p=0.000 | | p=0.000 | | p=0.000 | |  |
| Urban non-poor | 32.6 | 1,386 | 19.1 | 971 | 35.0 | 3,377 | 31.7 | 3,377 | 26.1 | 3,377 | 58.6 | 3,008 | 52.2 | 3,008 | 58.1 | 2,675 |  |
| Urban poor | 25.9 | 460 | 17.1 | 329 | 27.2 | 1,155 | 24.5 | 1,155 | 19.2 | 1,155 | 43.4 | 1,029 | 43.0 | 1,029 | 46.5 | 906 |  |
| Rural | 37.4 | 1,701 | 9.2 | 1,344 | 21.7 | 4,624 | 20.2 | 4,624 | 17.3 | 4,624 | 43.1 | 4,120 | 42.3 | 4,120 | 36.5 | 3,625 |  |
| **Wealth quintile** | p=0.051 | | p=0.000 | | p=0.000 | | p=0.000 | | p=0.000 | | p=0.000 | | p=0.000 | | p=0.000 | |  |
| Lowest | 31.6 | 807 | 6.2 | 637 | 16.2 | 2,228 | 15.1 | 2,228 | 13.4 | 2,228 | 35.8 | 1,986 | 36.9 | 1,986 | 29.9 | 1,747 |  |
| Second | 35.1 | 736 | 11.0 | 575 | 22.4 | 2,028 | 20.8 | 2,028 | 17.8 | 2,028 | 42.8 | 1,803 | 42.8 | 1,803 | 37.4 | 1,575 |  |
| Middle | 36.1 | 680 | 13.0 | 527 | 27.3 | 1,815 | 26.0 | 1,815 | 20.3 | 1,815 | 50.1 | 1,603 | 45.9 | 1,603 | 48.4 | 1,396 |  |
| Fourth | 29.4 | 726 | 15.4 | 473 | 33.8 | 1,676 | 30.8 | 1,676 | 25.7 | 1,676 | 59.1 | 1,508 | 51.7 | 1,508 | 56.3 | 1,357 |  |
| Highest | 39.3 | 597 | 28.1 | 432 | 44.2 | 1,408 | 38.6 | 1,408 | 31.7 | 1,408 | 64.4 | 1,258 | 58.6 | 1,258 | 66.1 | 1,130 |  |
| **Region** | p=0.000 | | p=0.000 | | p=0.000 | | p=0.000 | | p=0.000 | | p=0.000 | | p=0.000 | | p=0.000 | |  |
| Abidjan | 23.2 | 845 | 25.6 | 528 | 42.8 | 1,823 | 37.9 | 1,823 | 29.3 | 1,823 | 62.2 | 1,624 | 55.3 | 1,624 | 64.2 | 1,480 |  |
| Yamoussoukro | 45.7 | 42 | * | 31 | 39.1 | 115 | 36.8 | 115 | 30.8 | 115 | 68.2 | 100 | 49.2 | 100 | 58.7 | 88 |  |
| Bas Sassandra | 38.2 | 317 | 12.5 | 259 | 24.4 | 883 | 23.3 | 883 | 21.2 | 883 | 57.2 | 788 | 64.0 | 788 | 56.6 | 684 |  |
| Comoe | 41.2 | 137 | 22.1 | 110 | 41.7 | 389 | 40.7 | 389 | 33.5 | 389 | 61.3 | 344 | 48.8 | 344 | 60.7 | 300 |  |
| Denguele | 27.2 | 61 | * | 46 | 11.3 | 161 | 9.8 | 161 | 6.5 | 161 | 35.3 | 144 | 33.9 | 144 | 34.1 | 128 |  |
| Goh-Djiboua | 40.1 | 244 | 14.2 | 196 | 24.7 | 631 | 22.0 | 631 | 20.0 | 631 | 58.1 | 571 | 63.3 | 571 | 48.0 | 501 |  |
| Lacs | 46.2 | 168 | 18.4 | 126 | 34.5 | 413 | 33.2 | 413 | 26.7 | 413 | 58.8 | 367 | 45.3 | 367 | 47.7 | 324 |  |
| Lagunes | 33.9 | 196 | * | 163 | 27.8 | 589 | 26.3 | 589 | 21.3 | 589 | 53.4 | 529 | 38.5 | 529 | 54.4 | 466 |  |
| Montagnes | 37.1 | 364 | * | 267 | 20.0 | 946 | 17.2 | 946 | 11.9 | 946 | 41.3 | 833 | 48.4 | 833 | 38.8 | 737 |  |
| Sassandra-Marahoue | 26.2 | 368 | * | 290 | 18.8 | 996 | 17.1 | 996 | 16.0 | 996 | 31.5 | 890 | 24.1 | 890 | 23.3 | 782 |  |
| Savanes | 38.3 | 264 | * | 213 | 20.4 | 727 | 18.9 | 727 | 17.4 | 727 | 32.0 | 647 | 34.8 | 647 | 34.1 | 558 |  |
| Vallee du Bandama | 50.5 | 203 | * | 158 | 15.2 | 566 | 13.3 | 566 | 11.3 | 566 | 42.5 | 504 | 45.8 | 504 | 35.9 | 439 |  |
| Woroba | 22.5 | 165 | * | 122 | 13.1 | 452 | 12.4 | 452 | 11.2 | 452 | 32.7 | 402 | 26.1 | 402 | 22.8 | 357 |  |
| Zanzan | 48.3 | 172 | 17.2 | 135 | 31.8 | 464 | 31.0 | 464 | 28.9 | 464 | 45.7 | 413 | 43.1 | 413 | 38.1 | 363 |  |
| An asterisk indicates that the figure is based on 25 or fewer unweighted cases and has been suppressed. | | | | | | | | | | | | | | | | | |
| ^All basic antigen vaccines: 1 dose BCG, 3 doses Polio (OPV/IPV), 3 doses DPT, 1 dose measles (MR) | | | | | | | | | | | | | | | | | |
| & either source - vaccination card or mother's report (crude coverage)  Light gray shading indicates p<0.05.  Dark gray shading indicates p<0.001. | | | | | | | | | | | | | | | | | |

Supplementary Table 8: Health Delivery Platform and Nutrition Intervention Coverage during Antenatal Care by Background Characteristic - Ghana

|  | **Pregnancy/antenatal care (ANC)** | | | | | | | | | | | | | | | | | | | |
| --- | --- | --- | --- | --- | --- | --- | --- | --- | --- | --- | --- | --- | --- | --- | --- | --- | --- | --- | --- | --- |
|  | **Health delivery platform: Attended 4+ ANC visits** | | **Health delivery platform: Attended 8+ ANC visits** | | **Took iron-containing supplements for any number of days during most recent pregnancy** | | **Took iron-containing supplements for 90+ days during most recent pregnancy** | | **Took iron-containing supplements for 180+ days during most recent pregnancy** | | **Took intestinal parasite drugs during most recent pregnancy** | | **Counseled about maternal diet during ANC visit** | | **Counseled about breastfeeding during ANC visit** | | **Source of iron-containing supplements: public sector** | | **Source of iron-containing supplements: private sector + other** | |
|  | **%** | **N** | **%** | **N** | **%** | **N** | **%** | **N** | **%** | **N** | **%** | **N** | **%** | **N** | **%** | **N** | **%** | **N** | **%** | **N** |
| **Total** | 87.8 | 3,491 | 38.6 | 3,491 | 90.6 | 3,491 | 60.2 | 3,491 | 29.1 | 3,491 | 58.9 | 3,491 | 92.3 | 3,491 | 87.5 | 3,491 | 85.3 | 3,230 | 14.9 | 3,230 |
| **Background characteristic** |  |  |  |  |  |  |  |  |  |  |  |  |  |  |  |  |  |  |  |  |
| **Sex of child** | p=0.685 | | p=0.886 | | p=0.664 | | p=0.293 | | p=0.145 | | p=0.675 | | p=0.248 | | p=0.158 | | p=0.542 | | p=0.919 | |
| Male | 88.0 | 1,803 | 38.5 | 1,803 | 90.8 | 1,803 | 61.3 | 1,803 | 30.4 | 1,803 | 58.4 | 1,803 | 92.9 | 1,803 | 88.4 | 1,803 | 84.8 | 1,676 | 15.0 | 1,676 |
| Female | 87.5 | 1,688 | 38.8 | 1,688 | 90.3 | 1,688 | 59.1 | 1,688 | 27.7 | 1,688 | 59.4 | 1,688 | 91.7 | 1,688 | 86.5 | 1,688 | 85.9 | 1,554 | 14.8 | 1,554 |
| **Maternal age** | p=0.005 | | p=0.008 | | p=0.060 | | p=0.622 | | p=0.295 | | p=0.446 | | p=0.331 | | p=0.085 | | p=0.022 | | p=0.011 | |
| <20 | 80.3 | 239 | 26.5 | 239 | 87.4 | 239 | 63.0 | 239 | 24.0 | 239 | 55.2 | 239 | 90.0 | 239 | 82.8 | 239 | 94.5 | 218 | * | 218 |
| 20–34 | 88.2 | 2,442 | 39.2 | 2,442 | 91.5 | 2,442 | 60.3 | 2,442 | 29.3 | 2,442 | 59.7 | 2,442 | 92.2 | 2,442 | 87.3 | 2,442 | 84.7 | 2,273 | 15.6 | 2,273 |
| 35–49 | 88.8 | 810 | 40.3 | 810 | 88.8 | 810 | 59.1 | 810 | 30.0 | 810 | 57.6 | 810 | 93.4 | 810 | 89.5 | 810 | 84.6 | 740 | 15.7 | 740 |
| **Mother's education** | p=0.000 | | p=0.000 | | p=0.000 | | p=0.000 | | p=0.000 | | p=0.000 | | p=0.016 | | p=0.013 | | p=0.000 | | p=0.000 | |
| None | 80.8 | 728 | 27.0 | 728 | 83.6 | 728 | 48.7 | 728 | 18.8 | 728 | 52.2 | 728 | 88.9 | 728 | 83.1 | 728 | 95.4 | 626 | 4.8 | 626 |
| Primary | 84.8 | 542 | 31.5 | 542 | 88.5 | 542 | 56.1 | 542 | 25.0 | 542 | 53.8 | 542 | 92.5 | 542 | 87.0 | 542 | 85.0 | 491 | 16.9 | 491 |
| Secondary+ | 90.8 | 2,221 | 44.1 | 2,221 | 93.4 | 2,221 | 65.0 | 2,221 | 33.5 | 2,221 | 62.3 | 2,221 | 93.4 | 2,221 | 89.1 | 2,221 | 82.5 | 2,113 | 17.4 | 2,113 |
| **Place of residence** | p=0.000 | | p=0.000 | | p=0.033 | | p=0.021 | | p=0.000 | | p=0.155 | | p=0.028 | | p=0.000 | | p=0.000 | | p=0.000 | |
| Urban | 91.2 | 1,623 | 46.2 | 1,623 | 92.2 | 1,623 | 63.9 | 1,623 | 34.2 | 1,623 | 60.8 | 1,623 | 93.9 | 1,623 | 91.0 | 1,623 | 78.9 | 1,536 | 20.7 | 1,536 |
| Rural | 84.8 | 1,868 | 32.0 | 1,868 | 89.2 | 1,868 | 57.0 | 1,868 | 24.7 | 1,868 | 57.2 | 1,868 | 91.0 | 1,868 | 84.4 | 1,868 | 91.2 | 1,694 | 9.7 | 1,694 |
| **Urban poverty household** | p=0.000 | | p=0.000 | | p=0.051 | | p=0.004 | | p=0.000 | | p=0.332 | | p=0.062 | | p=0.000 | | p=0.000 | | p=0.000 | |
| Urban non-poor | 92.9 | 1,235 | 49.6 | 1,235 | 92.8 | 1,235 | 66.2 | 1,235 | 36.8 | 1,235 | 61.0 | 1,235 | 93.8 | 1,235 | 90.9 | 1,235 | 77.0 | 1,177 | 22.3 | 1,177 |
| Urban poor | 86.1 | 388 | 35.5 | 388 | 90.2 | 388 | 56.6 | 388 | 25.9 | 388 | 60.1 | 388 | 94.4 | 388 | 91.6 | 388 | 85.1 | 360 | 15.5 | 360 |
| Rural | 84.8 | 1,868 | 32.0 | 1,868 | 89.2 | 1,868 | 57.0 | 1,868 | 24.7 | 1,868 | 57.2 | 1,868 | 91.0 | 1,868 | 84.4 | 1,868 | 91.2 | 1,694 | 9.7 | 1,694 |
| **Wealth quintile** | p=0.000 | | p=0.000 | | p=0.000 | | p=0.000 | | p=0.000 | | p=0.002 | | p=0.002 | | p=0.001 | | p=0.000 | | p=0.000 | |
| Lowest | 77.8 | 853 | 23.5 | 853 | 86.1 | 853 | 48.4 | 853 | 17.8 | 853 | 52.3 | 853 | 88.1 | 853 | 82.0 | 853 | 93.6 | 744 | 6.8 | 744 |
| Second | 87.6 | 723 | 33.7 | 723 | 89.0 | 723 | 58.0 | 723 | 26.6 | 723 | 54.6 | 723 | 91.2 | 723 | 86.3 | 723 | 91.2 | 664 | 9.6 | 664 |
| Middle | 86.4 | 705 | 35.7 | 705 | 92.9 | 705 | 61.4 | 705 | 27.3 | 705 | 63.0 | 705 | 93.7 | 705 | 88.6 | 705 | 86.7 | 669 | 13.1 | 669 |
| Fourth | 94.5 | 631 | 46.9 | 631 | 92.2 | 631 | 66.0 | 631 | 34.6 | 631 | 65.0 | 631 | 93.9 | 631 | 90.5 | 631 | 83.0 | 600 | 17.4 | 600 |
| Highest | 97.0 | 579 | 61.6 | 579 | 94.6 | 579 | 72.7 | 579 | 45.2 | 579 | 62.3 | 579 | 96.7 | 579 | 92.4 | 579 | 68.0 | 553 | 31.7 | 553 |
| **Region** | p=0.003 | | p=0.000 | | p=0.000 | | p=0.000 | | p=0.000 | | p=0.000 | | p=0.001 | | p=0.000 | | p=0.000 | | p=0.003 | |
| Western | 89.4 | 208 | 46.5 | 208 | 90.5 | 208 | 57.9 | 208 | 30.9 | 208 | 58.9 | 208 | 91.6 | 208 | 90.7 | 208 | 84.7 | 190 | 16.8 | 190 |
| Central | 88.0 | 357 | 44.7 | 357 | 94.3 | 357 | 72.9 | 357 | 35.6 | 357 | 74.1 | 357 | 91.5 | 357 | 83.1 | 357 | 79.7 | 343 | 18.7 | 343 |
| Greater Accra | 90.1 | 410 | 49.3 | 410 | 93.0 | 410 | 70.1 | 410 | 40.8 | 410 | 56.7 | 410 | 94.6 | 410 | 90.8 | 410 | 72.4 | 395 | 23.4 | 395 |
| Volta | 94.0 | 130 | 45.3 | 130 | 90.7 | 130 | 78.6 | 130 | 44.5 | 130 | 73.5 | 130 | 94.9 | 130 | 96.5 | 130 | 91.3 | 127 | * | 127 |
| Eastern | 88.7 | 246 | 44.8 | 246 | 93.3 | 246 | 56.2 | 246 | 24.8 | 246 | 81.4 | 246 | 98.3 | 246 | 96.1 | 246 | 90.4 | 236 | * | 236 |
| Ashanti | 89.7 | 631 | 38.8 | 631 | 93.7 | 631 | 60.4 | 631 | 26.2 | 631 | 55.1 | 631 | 91.5 | 631 | 80.9 | 631 | 82.4 | 600 | 18.5 | 600 |
| Western North | 86.8 | 96 | 30.1 | 96 | 88.3 | 96 | 37.0 | 96 | * | 96 | 69.0 | 96 | 94.5 | 96 | 90.8 | 96 | 87.8 | 86 | * | 86 |
| Ahafo | 88.6 | 77 | 42.4 | 77 | 95.4 | 77 | 57.8 | 77 | 29.5 | 77 | 62.3 | 77 | 91.7 | 77 | 93.6 | 77 | 91.1 | 74 | 12.5 | 74 |
| Bono | 91.2 | 113 | 36.8 | 113 | 96.9 | 113 | 68.1 | 113 | 34.7 | 113 | 53.9 | 113 | 95.7 | 113 | 94.2 | 113 | 85.0 | 111 | * | 111 |
| Bono East | 85.6 | 191 | 33.7 | 191 | 86.6 | 191 | 63.6 | 191 | 31.1 | 191 | 52.0 | 191 | 91.5 | 191 | 90.5 | 191 | 92.4 | 165 | * | 165 |
| Oti | 75.7 | 123 | 24.9 | 123 | 90.8 | 123 | 68.7 | 123 | 32.9 | 123 | 61.9 | 123 | 94.1 | 123 | 91.4 | 123 | 91.2 | 117 | 10.9 | 117 |
| Northern | 80.8 | 395 | 27.7 | 395 | 82.2 | 395 | 47.2 | 395 | 18.3 | 395 | 56.2 | 395 | 87.3 | 395 | 83.6 | 395 | 91.9 | 329 | * | 329 |
| Savannah | 79.5 | 105 | 19.4 | 105 | 78.2 | 105 | 31.3 | 105 | 16.1 | 105 | 43.2 | 105 | 81.5 | 105 | 79.2 | 105 | 93.3 | 85 | * | 85 |
| North East | 83.5 | 112 | 28.7 | 112 | 87.5 | 112 | 58.5 | 112 | 12.6 | 112 | 30.4 | 112 | 87.0 | 112 | 68.9 | 112 | 94.8 | 100 | * | 100 |
| Upper East | 94.8 | 191 | 42.0 | 191 | 87.5 | 191 | 40.8 | 191 | 28.9 | 191 | 41.4 | 191 | 96.7 | 191 | 96.2 | 191 | 83.9 | 169 | 14.7 | 169 |
| Upper West | 94.8 | 105 | 32.9 | 105 | 95.7 | 105 | 81.5 | 105 | 38.9 | 105 | 58.5 | 105 | 97.5 | 105 | 93.4 | 105 | 95.2 | 103 | * | 103 |
| An asterisk indicates that the figure is based on 25 or fewer unweighted cases and has been suppressed.  Light gray shading indicates p<0.05.  Dark gray shading indicates p<0.001. | | | | | | | | | | | | | | | | | | | | |

*Continued…*

Supplementary Table 9: Health Delivery Platform and Nutrition Intervention Coverage during Birth and Postnatal Care by Background Characteristic - Ghana

|  | **Birth** | | | | | | **Postnatal care (PNC)** | | | | | | | |
| --- | --- | --- | --- | --- | --- | --- | --- | --- | --- | --- | --- | --- | --- | --- |
|  | **Health delivery platform: Live births delivered in a health facility** | | **Skin-to-skin contact immediately after birth** | | **Started breastfeeding within one hour of birth** | | **Health delivery platform: PNC check within two days for newborn** | | **Weighed during newborn PNC check** | | **Counseled about breastfeeding during newborn PNC check** | | **Observed breastfeeding during newborn PNC check** | |
|  | **%** | **N** | **%** | **N** | **%** | **N** | **%** | **N** | **%** | **N** | **%** | **N** | **%** | **N** |
| **Total** | 86.2 | 3,638 | 59.8 | 3,491 | 58.2 | 3,638 | 86.9 | 3,491 | 82.2 | 3,491 | 75.8 | 3,491 | 71.8 | 3,491 |
| **Background characteristic** |  |  |  |  |  |  |  |  |  |  |  |  |  |  |
| **Sex of child** | p=0.003 | | p=0.739 | | p=0.406 | | p=0.004 | | p=0.007 | | p=0.696 | | p=0.444 | |
| Male | 88.1 | 1,889 | 60.1 | 1,803 | 57.3 | 1,889 | 88.6 | 1,803 | 84.1 | 1,803 | 76.2 | 1,803 | 72.4 | 1,803 |
| Female | 84.2 | 1,749 | 59.4 | 1,688 | 59.1 | 1,749 | 85.0 | 1,688 | 80.1 | 1,688 | 75.5 | 1,688 | 71.1 | 1,688 |
| **Maternal age** | p=0.094 | | p=0.027 | | p=0.933 | | p=0.961 | | p=0.544 | | p=0.246 | | p=0.039 | |
| <20 | 88.8 | 245 | 63.9 | 239 | 59.0 | 245 | 86.8 | 239 | 82.9 | 239 | 73.8 | 239 | 65.7 | 239 |
| 20–34 | 86.8 | 2,550 | 61.0 | 2,442 | 58.3 | 2,550 | 86.8 | 2,442 | 82.6 | 2,442 | 76.8 | 2,442 | 73.4 | 2,442 |
| 35–49 | 83.8 | 842 | 54.9 | 810 | 57.5 | 842 | 87.2 | 810 | 80.7 | 810 | 73.5 | 810 | 68.7 | 810 |
| **Mother's education** | p=0.000 | | p=0.028 | | p=0.260 | | p=0.000 | | p=0.000 | | p=0.000 | | p=0.000 | |
| None | 72.8 | 761 | 54.9 | 728 | 61.6 | 761 | 79.0 | 728 | 66.6 | 728 | 61.3 | 728 | 55.3 | 728 |
| Primary | 82.7 | 562 | 57.4 | 542 | 58.2 | 562 | 86.3 | 542 | 75.3 | 542 | 74.8 | 542 | 68.9 | 542 |
| Secondary+ | 91.5 | 2,315 | 62.0 | 2,221 | 57.0 | 2,315 | 89.6 | 2,221 | 89.0 | 2,221 | 80.9 | 2,221 | 77.9 | 2,221 |
| **Place of residence** | p=0.000 | | p=0.475 | | p=0.155 | | p=0.000 | | p=0.000 | |  | | p=0.000 | |
| Urban | 94.3 | 1,700 | 60.6 | 1,623 | 56.4 | 1,700 | 90.5 | 1,623 | 90.4 | 1,623 | 81.0 | 1,623 | 77.5 | 1,623 |
| Rural | 79.1 | 1,938 | 59.0 | 1,868 | 59.7 | 1,938 | 83.7 | 1,868 | 75.1 | 1,868 | 71.4 | 1,868 | 66.8 | 1,868 |
| **Urban poverty household** | p=0.000 | | p=0.725 | | p=0.368 | | p=0.000 | | p=0.000 | | p=0.000 | | p=0.000 | |
| Urban non-poor | 95.1 | 1,284 | 60.7 | 1,235 | 56.2 | 1,284 | 91.9 | 1,235 | 92.6 | 1,235 | 82.6 | 1,235 | 78.8 | 1,235 |
| Urban poor | 92.0 | 416 | 60.5 | 388 | 57.1 | 416 | 86.2 | 388 | 83.5 | 388 | 75.9 | 388 | 73.1 | 388 |
| Rural | 79.1 | 1,938 | 59.0 | 1,868 | 59.7 | 1,938 | 83.7 | 1,868 | 75.1 | 1,868 | 71.4 | 1,868 | 66.8 | 1,868 |
| **Wealth quintile** | p=0.000 | | p=0.013 | | p=0.023 | | p=0.000 | | p=0.000 | | p=0.000 | | p=0.000 | |
| Lowest | 71.4 | 896 | 53.1 | 853 | 60.6 | 896 | 78.8 | 853 | 64.8 | 853 | 63.4 | 853 | 59.9 | 853 |
| Second | 86.3 | 749 | 61.6 | 723 | 62.4 | 749 | 86.7 | 723 | 80.6 | 723 | 73.4 | 723 | 68.1 | 723 |
| Middle | 87.0 | 730 | 64.4 | 705 | 59.1 | 730 | 89.2 | 705 | 83.1 | 705 | 78.9 | 705 | 75.8 | 705 |
| Fourth | 95.5 | 668 | 59.9 | 631 | 56.4 | 668 | 90.0 | 631 | 92.6 | 631 | 83.2 | 631 | 78.0 | 631 |
| Highest | 97.2 | 595 | 61.5 | 579 | 50.1 | 595 | 92.8 | 579 | 97.4 | 579 | 85.4 | 579 | 82.1 | 579 |
| **Region** | p=0.000 | | p=0.000 | | p=0.000 | | p=0.000 | | p=0.000 | | p=0.000 | | p=0.000 | |
| Western | 85.0 | 212 | 63.3 | 208 | 65.0 | 212 | 89.1 | 208 | 81.3 | 208 | 82.5 | 208 | 79.2 | 208 |
| Central | 82.9 | 380 | 55.2 | 357 | 51.5 | 380 | 89.2 | 357 | 79.0 | 357 | 75.4 | 357 | 74.1 | 357 |
| Greater Accra | 92.3 | 427 | 60.5 | 410 | 43.8 | 427 | 93.2 | 410 | 90.5 | 410 | 82.8 | 410 | 78.4 | 410 |
| Volta | 90.9 | 135 | 60.6 | 130 | 68.4 | 135 | 88.5 | 130 | 86.7 | 130 | 89.9 | 130 | 84.7 | 130 |
| Eastern | 89.5 | 252 | 67.0 | 246 | 48.3 | 252 | 94.3 | 246 | 89.3 | 246 | 85.8 | 246 | 83.2 | 246 |
| Ashanti | 92.4 | 666 | 54.9 | 631 | 61.2 | 666 | 85.8 | 631 | 89.3 | 631 | 76.9 | 631 | 68.8 | 631 |
| Western North | 88.9 | 101 | 73.3 | 96 | 64.0 | 101 | 84.9 | 96 | 83.0 | 96 | 70.5 | 96 | 68.3 | 96 |
| Ahafo | 92.2 | 81 | 57.2 | 77 | 43.9 | 81 | 83.8 | 77 | 90.3 | 77 | 86.3 | 77 | 86.8 | 77 |
| Bono | 90.7 | 117 | 67.0 | 113 | 63.5 | 117 | 95.7 | 113 | 89.5 | 113 | 79.2 | 113 | 77.8 | 113 |
| Bono East | 86.2 | 202 | 63.2 | 191 | 70.6 | 202 | 86.9 | 191 | 82.8 | 191 | 76.9 | 191 | 70.4 | 191 |
| Oti | 67.1 | 128 | 53.4 | 123 | 65.1 | 128 | 75.6 | 123 | 63.3 | 123 | 76.8 | 123 | 74.2 | 123 |
| Northern | 70.3 | 406 | 45.7 | 395 | 56.5 | 406 | 73.0 | 395 | 64.7 | 395 | 56.4 | 395 | 52.8 | 395 |
| Savannah | 70.6 | 111 | 68.8 | 105 | 63.3 | 111 | 81.5 | 105 | 57.7 | 105 | 72.8 | 105 | 69.4 | 105 |
| North East | 84.4 | 116 | 78.7 | 112 | 66.8 | 116 | 84.1 | 112 | 77.9 | 112 | 65.5 | 112 | 61.9 | 112 |
| Upper East | 97.4 | 196 | 63.7 | 191 | 66.4 | 196 | 95.2 | 191 | 88.9 | 191 | 79.1 | 191 | 76.9 | 191 |
| Upper West | 94.0 | 109 | 76.7 | 105 | 61.2 | 109 | 89.6 | 105 | 86.4 | 105 | 61.9 | 105 | 58.5 | 105 |

Light gray shading indicates p<0.05.

Dark gray shading indicates p<0.001.

*Continued…*

Supplementary Table 10: Health Delivery Platform and Nutrition Intervention Coverage during Infant and Young Childhood by Background Characteristic - Ghana

|  | **Infancy and childhood** | | | | | | | | | | | | | | | |  |
| --- | --- | --- | --- | --- | --- | --- | --- | --- | --- | --- | --- | --- | --- | --- | --- | --- | --- |
|  | **Health Delivery platform: All basic vaccinations^ according to either source^&^ (12–35 mos)** | | **Mothers of children age 6–23 mos who received IYCF counseling in last 6 mos** | | **Child under 5 with weight measured in the last 3 mos** | | **Child under 5 with height measured in the last 3 mos** | | **Child under 5 with MUAC measured in the last 3 mos** | | **Children age 6–59 mos given iron containing supplements** | | **Children age 6–59 mos given Vit. A supplements** | | **Children age 12–59 mos given deworming medication** | |  |
|  | **%** | **N** | **%** | **N** | **%** | **N** | **%** | **N** | **%** | **N** | **%** | **N** | **%** | **N** | **%** | **N** |  |
| **Total** | 73.2 | 3,369 | 49.5 | 2,562 | 49.6 | 8,315 | 38.5 | 8,315 | 21.6 | 8,315 | 51.3 | 7,465 | 74.6 | 7,465 | 46.3 | 6,597 |  |
| **Background characteristic** |  |  |  |  |  |  |  |  |  |  |  |  |  |  |  |  |  |
| **Sex of child** | p=0.385 | | p=0.638 | | p=0.952 | | p=0.683 | | p=0.242 | | p=0.601 | | p=0.345 | | p=0.813 | |  |
| Male | 74.0 | 1,692 | 50.1 | 1,290 | 49.6 | 4,240 | 38.7 | 4,240 | 22.2 | 4,240 | 50.9 | 3,764 | 74.1 | 3,764 | 46.1 | 3,326 |  |
| Female | 72.4 | 1,677 | 49.0 | 1,273 | 49.6 | 4,075 | 38.2 | 4,075 | 20.9 | 4,075 | 51.7 | 3,700 | 75.2 | 3,700 | 46.4 | 3,271 |  |
| **Child's age in months** |  |  |  |  | p=0.000 | | p=0.000 | | p=0.000 | |  | |  |  |  |  |  |
| 0–23 |  |  |  |  | 75.7 | 3,541 | 57.5 | 3,541 | 30.9 | 3,541 |  |  |  |  |  |  |  |
| 24–59 |  |  |  |  | 30.3 | 4,774 | 24.3 | 4,774 | 14.7 | 4,774 |  |  |  |  |  |  |  |
| **Maternal age** | p=0.155 | | p=0.221 | | p=0.000 | | p=0.000 | | p=0.052 | | p=0.261 | | p=0.065 | | p=0.012 | |  |
| <20 | 65.1 | 132 | 44.0 | 150 | 65.7 | 299 | 53.2 | 299 | 27.0 | 299 | 48.5 | 230 | 83.6 | 230 | 42.1 | 160 |  |
| 20–34 | 72.9 | 2,294 | 48.8 | 1,788 | 50.8 | 5,495 | 39.1 | 5,495 | 22.0 | 5,495 | 50.6 | 4,883 | 74.4 | 4,883 | 44.7 | 4,286 |  |
| 35–49 | 74.9 | 943 | 52.9 | 624 | 45.1 | 2,520 | 35.3 | 2,520 | 20.1 | 2,520 | 53.0 | 2,351 | 74.3 | 2,351 | 49.7 | 2,151 |  |
| **Mother's education** | p=0.000 | | p=0.000 | | p=0.012 | | p=0.233 | | p=0.008 | | p=0.000 | | p=0.000 | | p=0.000 | |  |
| None | 64.3 | 766 | 36.7 | 540 | 46.5 | 1,922 | 38.2 | 1,922 | 24.7 | 1,922 | 40.2 | 1,746 | 67.1 | 1,746 | 34.9 | 1,570 |  |
| Primary | 72.5 | 522 | 45.8 | 397 | 47.4 | 1,250 | 35.8 | 1,250 | 20.6 | 1,250 | 49.4 | 1,119 | 73.5 | 1,119 | 38.4 | 998 |  |
| Secondary+ | 76.6 | 2,081 | 54.7 | 1,625 | 51.3 | 5,142 | 39.2 | 5,142 | 20.7 | 5,142 | 55.9 | 4,600 | 77.7 | 4,600 | 52.6 | 4,028 |  |
| **Place of residence** | p=0.317 | | p=0.016 | | p=0.000 | | p=0.006 | | p=0.126 | | p=0.000 | | p=0.517 | |  | |  |
| Urban | 74.2 | 1,644 | 53.3 | 1,206 | 45.6 | 4,048 | 35.7 | 4,048 | 20.3 | 4,048 | 55.5 | 3,668 | 75.2 | 3,668 | 51.7 | 3,255 |  |
| Rural | 72.1 | 1,725 | 46.2 | 1,356 | 53.4 | 4,267 | 41.1 | 4,267 | 22.8 | 4,267 | 47.1 | 3,796 | 74.0 | 3,796 | 41.0 | 3,342 |  |
| **Urban poverty household** | p=0.234 | | p=0.018 | | p=0.000 | | p=0.007 | | p=0.216 | | p=0.000 | | p=0.765 | | p=0.000 | |  |
| Urban non-poor | 75.4 | 1,221 | 54.5 | 902 | 46.5 | 3,087 | 36.4 | 3,087 | 20.6 | 3,087 | 56.6 | 2,780 | 75.2 | 2,780 | 53.8 | 2,454 |  |
| Urban poor | 70.8 | 423 | 49.9 | 304 | 42.4 | 961 | 33.4 | 961 | 19.6 | 961 | 52.3 | 888 | 75.3 | 888 | 45.0 | 801 |  |
| Rural | 72.1 | 1,725 | 46.2 | 1,356 | 53.4 | 4,267 | 41.1 | 4,267 | 22.8 | 4,267 | 47.1 | 3,796 | 74.0 | 3,796 | 41.0 | 3,342 |  |
| **Wealth quintile** | p=0.000 | | p=0.000 | | p=0.018 | | p=0.001 | | p=0.000 | | p=0.000 | | p=0.005 | | p=0.000 | |  |
| Lowest | 66.8 | 773 | 38.4 | 613 | 52.0 | 1,966 | 42.7 | 1,966 | 26.2 | 1,966 | 40.6 | 1,746 | 69.7 | 1,746 | 34.0 | 1,533 |  |
| Second | 71.0 | 720 | 48.0 | 520 | 53.4 | 1,690 | 41.4 | 1,690 | 24.3 | 1,690 | 47.6 | 1,512 | 77.0 | 1,512 | 40.6 | 1,356 |  |
| Middle | 74.1 | 677 | 50.3 | 544 | 48.8 | 1,614 | 35.3 | 1,614 | 20.6 | 1,614 | 51.9 | 1,467 | 73.5 | 1,467 | 46.3 | 1,279 |  |
| Fourth | 75.5 | 618 | 57.1 | 454 | 46.2 | 1,584 | 33.6 | 1,584 | 16.7 | 1,584 | 60.8 | 1,411 | 75.9 | 1,411 | 53.6 | 1,241 |  |
| Highest | 80.9 | 580 | 58.4 | 431 | 46.5 | 1,460 | 38.2 | 1,460 | 18.7 | 1,460 | 58.6 | 1,329 | 78.2 | 1,329 | 60.9 | 1,188 |  |
| **Region** | p=0.000 | | p=0.000 | | p=0.000 | | p=0.000 | | p=0.000 | | p=0.000 | | p=0.000 | | p=0.000 | |  |
| Western | 65.3 | 215 | 49.0 | 157 | 43.1 | 515 | 35.3 | 515 | 23.2 | 515 | 59.7 | 473 | 71.5 | 473 | 48.2 | 420 |  |
| Central | 74.9 | 345 | 53.5 | 268 | 50.3 | 841 | 33.0 | 841 | 6.8 | 841 | 59.2 | 760 | 78.9 | 760 | 52.0 | 666 |  |
| Greater Accra | 80.7 | 417 | 65.7 | 289 | 36.3 | 1,057 | 26.9 | 1,057 | 13.6 | 1,057 | 51.5 | 943 | 72.4 | 943 | 51.1 | 848 |  |
| Volta | 84.0 | 135 | 63.9 | 107 | 61.5 | 313 | 53.3 | 313 | 25.0 | 313 | 41.7 | 290 | 74.9 | 290 | 35.3 | 255 |  |
| Eastern | 71.1 | 229 | 52.3 | 175 | 48.3 | 611 | 35.1 | 611 | 21.4 | 611 | 60.0 | 546 | 84.7 | 546 | 58.2 | 481 |  |
| Ashanti | 77.0 | 614 | 47.5 | 476 | 44.0 | 1,495 | 30.9 | 1,495 | 11.8 | 1,495 | 59.0 | 1,360 | 75.7 | 1,360 | 55.7 | 1,218 |  |
| Western North | 83.6 | 93 | 55.8 | 72 | 58.4 | 222 | 55.3 | 222 | 33.6 | 222 | 52.2 | 198 | 72.4 | 198 | 52.3 | 173 |  |
| Ahafo | 71.4 | 73 | 44.6 | 54 | 52.4 | 186 | 42.1 | 186 | 13.5 | 186 | 53.6 | 164 | 81.5 | 164 | 53.5 | 144 |  |
| Bono | 76.6 | 119 | 61.0 | 85 | 64.3 | 277 | 44.5 | 277 | 33.3 | 277 | 47.8 | 251 | 83.3 | 251 | 41.1 | 225 |  |
| Bono East | 77.3 | 183 | 25.7 | 137 | 56.4 | 437 | 50.5 | 437 | 27.5 | 437 | 47.6 | 390 | 67.6 | 390 | 42.6 | 347 |  |
| Oti | 78.6 | 104 | 44.7 | 82 | 63.6 | 276 | 54.9 | 276 | 34.8 | 276 | 37.0 | 238 | 78.5 | 238 | 36.7 | 208 |  |
| Northern | 53.0 | 368 | 38.0 | 302 | 41.3 | 923 | 34.1 | 923 | 24.5 | 923 | 32.0 | 837 | 60.9 | 837 | 29.3 | 722 |  |
| Savannah | 71.0 | 102 | 31.4 | 72 | 51.7 | 247 | 46.5 | 247 | 32.1 | 247 | 51.7 | 216 | 67.6 | 216 | 30.1 | 192 |  |
| North East | 80.5 | 110 | 40.8 | 76 | 61.8 | 267 | 44.0 | 267 | 34.0 | 267 | 50.6 | 231 | 78.8 | 231 | 30.5 | 206 |  |
| Upper East | 64.5 | 161 | 65.1 | 133 | 75.2 | 406 | 60.6 | 406 | 54.4 | 406 | 53.4 | 350 | 87.3 | 350 | 48.2 | 300 |  |
| Upper West | 75.8 | 102 | 39.6 | 79 | 59.5 | 242 | 50.2 | 242 | 26.3 | 242 | 40.8 | 218 | 74.2 | 218 | 31.3 | 195 |  |
| ^All basic antigen vaccines: 1 dose BCG, 3 doses Polio (OPV/IPV), 3 doses DPT, 1 dose measles (MR) | | | | | | | | | | | | | | | | | |
| & either source - vaccination card or mother's report (crude coverage)  Light gray shading indicates p<0.05.  Dark gray shading indicates p<0.001. | | | | | | | | | | | | | | | | | |

Supplementary Table 11: Health Delivery Platform and Nutrition Intervention Coverage during Antenatal Care by Background Characteristic - Kenya

|  | **Pregnancy/antenatal care (ANC)** | | | | | | | | | | | | | | | | | | | |
| --- | --- | --- | --- | --- | --- | --- | --- | --- | --- | --- | --- | --- | --- | --- | --- | --- | --- | --- | --- | --- |
|  | **Health delivery platform: Attended 4+ ANC visits** | | **Health delivery platform: Attended 8+ ANC visits** | | **Took iron-containing supplements for any number of days during most recent pregnancy** | | **Took iron-containing supplements for 90+ days during most recent pregnancy** | | **Took iron-containing supplements for 180+ days during most recent pregnancy** | | **Took intestinal parasite drugs during most recent pregnancy** | | **Counseled about maternal diet during ANC visit** | | **Counseled about breastfeeding during ANC visit** | | **Source of iron-containing supplements: public sector** | | **Source of iron-containing supplements: private sector + other** | |
|  | **%** | **N** | **%** | **N** | **%** | **N** | **%** | **N** | **%** | **N** | **%** | **N** | **%** | **N** | **%** | **N** | **%** | **N** | **%** | **N** |
| **Total** | 66.0 | 6,847 | 4.0 | 6,847 | 89.2 | 6,847 | 54.6 | 6,847 | 17.3 | 6,847 | 27.9 | 3,523 | 82.2 | 6,847 | 79.7 | 6,847 | 81.5 | 6,309 | 20.4 | 6,309 |
| **Background characteristic** |  |  |  |  |  |  |  |  |  |  |  |  |  |  |  |  |  |  |  |  |
| **Sex of child** | p=0.124 | | p=0.078 | | p=0.982 | | p=0.558 | | p=0.018 | | p=0.486 | | p=0.217 | | p=0.684 | | p=0.692 | | p=0.818 | |
| Male | 64.9 | 3,479 | 3.4 | 3,479 | 89.2 | 3,479 | 54.1 | 3,479 | 15.9 | 3,479 | 28.6 | 1,749 | 82.9 | 3,479 | 80.0 | 3,479 | 81.8 | 3,222 | 20.3 | 3,222 |
| Female | 67.2 | 3,369 | 4.6 | 3,369 | 89.2 | 3,369 | 55.0 | 3,369 | 18.7 | 3,369 | 27.2 | 1,775 | 81.5 | 3,369 | 79.5 | 3,369 | 81.2 | 3,087 | 20.6 | 3,087 |
| **Maternal age** | p=0.000 | | p=0.113 | | p=0.000 | | p=0.000 | | p=0.010 | | p=0.045 | | p=0.000 | | p=0.000 | | p=0.002 | | p=0.001 | |
| <20 | 54.0 | 606 | * | 606 | 84.6 | 606 | 45.5 | 606 | 12.6 | 606 | 20.4 | 299 | 75.6 | 606 | 71.5 | 606 | 89.2 | 529 | 12.5 | 529 |
| 20–34 | 68.6 | 5,140 | 4.3 | 5,140 | 90.4 | 5,140 | 56.8 | 5,140 | 18.1 | 5,140 | 28.5 | 2,671 | 83.3 | 5,140 | 81.2 | 5,140 | 80.6 | 4,802 | 21.5 | 4,802 |
| 35–49 | 60.8 | 1,101 | 3.6 | 1,101 | 86.4 | 1,101 | 49.0 | 1,101 | 16.2 | 1,101 | 29.1 | 554 | 80.7 | 1,101 | 77.3 | 1,101 | 81.8 | 978 | 19.3 | 978 |
| **Mother's education** | p=0.000 | | p=0.004 | | p=0.000 | | p=0.000 | | p=0.001 | | p=0.055 | | p=0.000 | | p=0.000 | | p=0.000 | | p=0.000 | |
| None | 49.1 | 639 | * | 639 | 78.7 | 639 | 40.2 | 639 | 16.7 | 639 | 21.7 | 340 | 68.4 | 639 | 63.0 | 639 | 93.2 | 515 | 7.0 | 515 |
| Primary | 59.6 | 2,417 | 3.5 | 2,417 | 88.0 | 2,417 | 49.2 | 2,417 | 14.5 | 2,417 | 29.8 | 1,232 | 79.6 | 2,417 | 77.7 | 2,417 | 86.6 | 2,192 | 14.1 | 2,192 |
| Secondary+ | 73.0 | 3,792 | 4.8 | 3,792 | 91.8 | 3,792 | 60.4 | 3,792 | 19.1 | 3,792 | 27.8 | 1,951 | 86.2 | 3,792 | 83.9 | 3,792 | 76.7 | 3,601 | 26.2 | 3,601 |
| **Place of residence** | p=0.000 | | p=0.001 | | p=0.471 | | p=0.011 | | p=0.102 | | p=0.934 | | p=0.000 | | p=0.000 | | p=0.000 | | p=0.000 | |
| Urban | 74.1 | 2,470 | 5.5 | 2,470 | 89.7 | 2,470 | 57.8 | 2,470 | 18.9 | 2,470 | 28.0 | 1,273 | 88.0 | 2,470 | 87.2 | 2,470 | 70.7 | 2,307 | 31.9 | 2,307 |
| Rural | 61.5 | 4,377 | 3.2 | 4,377 | 88.9 | 4,377 | 52.7 | 4,377 | 16.4 | 4,377 | 27.9 | 2,251 | 78.9 | 4,377 | 75.5 | 4,377 | 87.7 | 4,002 | 13.8 | 4,002 |
| **Urban poverty household** | p=0.000 | | p=0.002 | | p=0.293 | | p=0.000 | | p=0.000 | | p=0.242 | | p=0.000 | | p=0.000 | | p=0.000 | | p=0.000 | |
| Urban non-poor | 77.1 | 1,822 | 6.2 | 1,822 | 90.5 | 1,822 | 61.1 | 1,822 | 21.8 | 1,822 | 30.0 | 937 | 89.0 | 1,822 | 88.2 | 1,822 | 66.5 | 1,731 | 36.3 | 1,731 |
| Urban poor | 65.4 | 648 | * | 648 | 87.6 | 648 | 48.2 | 648 | 10.5 | 648 | 22.6 | 336 | 85.3 | 648 | 84.3 | 648 | 83.5 | 576 | 18.7 | 576 |
| Rural | 61.5 | 4,377 | 3.2 | 4,377 | 88.9 | 4,377 | 52.7 | 4,377 | 16.4 | 4,377 | 27.9 | 2,251 | 78.9 | 4,377 | 75.5 | 4,377 | 87.7 | 4,002 | 13.8 | 4,002 |
| **Wealth quintile** | p=0.000 | | p=0.000 | | p=0.000 | | p=0.000 | | p=0.000 | | p=0.718 | | p=0.000 | | p=0.000 | | p=0.000 | | p=0.000 | |
| Lowest | 53.9 | 1,538 | 2.3 | 1,538 | 85.7 | 1,538 | 48.7 | 1,538 | 15.8 | 1,538 | 26.3 | 765 | 72.8 | 1,538 | 68.7 | 1,538 | 91.8 | 1,343 | 8.5 | 1,343 |
| Second | 59.5 | 1,244 | 3.3 | 1,244 | 88.4 | 1,244 | 51.1 | 1,244 | 15.4 | 1,244 | 28.3 | 648 | 78.1 | 1,244 | 76.3 | 1,244 | 89.5 | 1,127 | 11.7 | 1,127 |
| Middle | 65.3 | 1,234 | 3.2 | 1,234 | 89.2 | 1,234 | 55.1 | 1,234 | 15.6 | 1,234 | 26.7 | 630 | 84.0 | 1,234 | 81.3 | 1,234 | 88.5 | 1,150 | 13.3 | 1,150 |
| Fourth | 69.6 | 1,414 | 3.4 | 1,414 | 90.6 | 1,414 | 54.6 | 1,414 | 14.7 | 1,414 | 28.1 | 772 | 87.3 | 1,414 | 84.6 | 1,414 | 81.5 | 1,328 | 21.1 | 1,328 |
| Highest | 82.0 | 1,417 | 8.0 | 1,417 | 92.4 | 1,417 | 63.4 | 1,417 | 24.6 | 1,417 | 30.2 | 709 | 89.4 | 1,417 | 88.5 | 1,417 | 58.8 | 1,362 | 44.8 | 1,362 |
| **Region** | p=0.000 | | p=0.001 | | p=0.000 | | p=0.000 | | p=0.000 | | p=0.000 | | p=0.000 | | p=0.000 | | p=0.000 | | p=0.000 | |
| Mombasa | 65.3 | 170 | * | 170 | 92.4 | 170 | 45.9 | 170 | * | 170 | 46.0 | 94 | 94.0 | 170 | 93.4 | 170 | 68.0 | 160 | 34.4 | 160 |
| Kwale | 71.9 | 113 | * | 113 | 96.2 | 113 | 72.2 | 113 | 18.1 | 113 | 69.9 | 60 | 94.6 | 113 | 93.4 | 113 | 97.5 | 109 | * | 109 |
| Kilifi | 77.3 | 188 | * | 188 | 95.1 | 188 | 66.8 | 188 | 19.3 | 188 | 68.0 | 93 | 88.0 | 188 | 83.9 | 188 | 94.0 | 181 | * | 181 |
| Tana River | 61.2 | 55 | * | 55 | 86.1 | 55 | 49.1 | 55 | 22.2 | 55 | 51.3 | 29 | 86.0 | 55 | 81.8 | 55 | 98.8 | 48 | * | 48 |
| Lamu | 70.5 | 27 | * | 27 | 86.9 | 27 | 54.7 | 27 | * | 27 | 77.8 | 16 | 79.7 | 27 | 61.1 | 27 | 95.5 | 25 | * | 25 |
| Taita Taveta | 64.9 | 52 | * | 52 | 92.8 | 52 | 66.2 | 52 | * | 52 | 68.0 | 24 | 76.9 | 52 | 78.3 | 52 | 89.3 | 48 | * | 48 |
| Garissa | 31.2 | 85 | * | 85 | 48.0 | 85 | 9.7 | 85 | * | 85 | * | 47 | 64.3 | 85 | 59.7 | 85 | 95.7 | 44 | * | 44 |
| Wajir | 44.9 | 52 | * | 52 | 71.5 | 52 | 12.1 | 52 | * | 52 | * | 27 | 39.2 | 52 | 38.5 | 52 | 90.4 | 42 | * | 42 |
| Mandera | 40.4 | 89 | * | 89 | 60.7 | 89 | 14.2 | 89 | 10.0 | 89 | * | 47 | 54.1 | 89 | 47.2 | 89 | 96.8 | 55 | * | 55 |
| Marsabit | 67.1 | 53 | * | 53 | 87.0 | 53 | 42.9 | 53 | * | 53 | * | 28 | 84.4 | 53 | 83.3 | 53 | 98.3 | 46 | * | 46 |
| Isiolo | 52.9 | 36 | * | 36 | 76.5 | 36 | 53.7 | 36 | 25.0 | 36 | 32.8 | 21 | 85.4 | 36 | 90.2 | 36 | 92.3 | 31 | * | 31 |
| Meru | 45.0 | 206 | * | 206 | 83.4 | 206 | 28.0 | 206 | * | 206 | * | 98 | 70.2 | 206 | 57.4 | 206 | 70.4 | 180 | 31.4 | 180 |
| Tharaka-Nithi | 63.2 | 49 | * | 49 | 93.3 | 49 | 59.3 | 49 | * | 49 | * | 26 | 83.4 | 49 | 79.1 | 49 | 86.0 | 46 | * | 46 |
| Embu | 62.0 | 64 | * | 64 | 87.8 | 64 | 67.2 | 64 | * | 64 | 70.0 | 31 | 91.2 | 64 | 76.0 | 64 | 85.1 | 57 | * | 57 |
| Kitui | 68.2 | 142 | * | 142 | 88.6 | 142 | 66.2 | 142 | 25.2 | 142 | * | 72 | 81.9 | 142 | 84.5 | 142 | 90.2 | 127 | * | 127 |
| Machakos | 76.9 | 154 | * | 154 | 91.6 | 154 | 61.6 | 154 | * | 154 | * | 76 | 88.5 | 154 | 87.8 | 154 | 75.4 | 144 | 31.6 | 144 |
| Makueni | 75.7 | 121 | * | 121 | 93.1 | 121 | 72.5 | 121 | * | 121 | * | 60 | 88.9 | 121 | 86.7 | 121 | 89.9 | 113 | * | 113 |
| Nyandarua | 60.6 | 82 | * | 82 | 92.2 | 82 | 54.2 | 82 | * | 82 | * | 47 | 85.8 | 82 | 83.4 | 82 | 88.9 | 81 | * | 81 |
| Nyeri | 82.2 | 85 | * | 85 | 90.6 | 85 | 56.1 | 85 | * | 85 | * | 49 | 80.4 | 85 | 78.8 | 85 | 88.5 | 78 | * | 78 |
| Kirinyaga | 67.6 | 81 | * | 81 | 82.5 | 81 | 57.8 | 81 | * | 81 | 54.6 | 45 | 91.0 | 81 | 92.8 | 81 | 89.2 | 79 | * | 79 |
| Murang'a | 58.3 | 134 | * | 134 | 90.9 | 134 | 60.3 | 134 | * | 134 | * | 69 | 88.8 | 134 | 90.2 | 134 | 85.8 | 125 | 28.1 | 125 |
| Kiambu | 66.8 | 412 | * | 412 | 90.3 | 412 | 48.6 | 412 | * | 412 | * | 199 | 89.5 | 412 | 92.1 | 412 | 72.1 | 396 | 27.9 | 396 |
| Turkana | 57.7 | 126 | * | 126 | 96.9 | 126 | 76.1 | 126 | 47.5 | 126 | 24.1 | 64 | 83.4 | 126 | 77.4 | 126 | 87.4 | 124 | 13.4 | 124 |
| West Pokot | 35.0 | 174 | * | 174 | 87.2 | 174 | 47.0 | 174 | 10.6 | 174 | * | 87 | 77.3 | 174 | 65.2 | 174 | 87.8 | 153 | 12.6 | 153 |
| Samburu | 56.3 | 59 | * | 59 | 84.0 | 59 | 52.6 | 59 | 23.1 | 59 | * | 29 | 64.0 | 59 | 55.6 | 59 | 86.2 | 52 | 15.3 | 52 |
| Trans Nzoia | 68.1 | 135 | * | 135 | 92.9 | 135 | 52.7 | 135 | * | 135 | * | 77 | 84.5 | 135 | 82.3 | 135 | 88.2 | 131 | * | 131 |
| Uasin Gishu | 71.9 | 210 | * | 210 | 93.5 | 210 | 54.6 | 210 | * | 210 | * | 106 | 85.8 | 210 | 82.3 | 210 | 89.0 | 200 | * | 200 |
| Elgeyo-Marakwet | 51.5 | 67 | * | 67 | 85.4 | 67 | 39.1 | 67 | * | 67 | * | 33 | 73.0 | 67 | 64.5 | 67 | 82.8 | 58 | 18.6 | 58 |
| Nandi | 62.1 | 116 | * | 116 | 80.5 | 116 | 44.6 | 116 | * | 116 | * | 58 | 83.5 | 116 | 74.1 | 116 | 86.0 | 104 | * | 104 |
| Baringo | 49.4 | 99 | * | 99 | 84.2 | 99 | 45.4 | 99 | * | 99 | 30.3 | 52 | 74.8 | 99 | 77.5 | 99 | 74.9 | 86 | 25.6 | 86 |
| Laikipia | 65.5 | 64 | * | 64 | 91.7 | 64 | 60.5 | 64 | * | 64 | * | 32 | 83.2 | 64 | 84.6 | 64 | 86.6 | 61 | * | 61 |
| Nakuru | 73.4 | 334 | * | 334 | 93.5 | 334 | 56.5 | 334 | * | 334 | * | 166 | 81.0 | 334 | 82.4 | 334 | 78.8 | 318 | 21.6 | 318 |
| Narok | 55.3 | 235 | * | 235 | 83.8 | 235 | 33.0 | 235 | * | 235 | * | 119 | 63.5 | 235 | 50.4 | 235 | 84.1 | 200 | 17.7 | 200 |
| Kajiado | 81.2 | 204 | * | 204 | 94.8 | 204 | 59.1 | 204 | 20.8 | 204 | * | 102 | 84.8 | 204 | 83.9 | 204 | 75.5 | 197 | 27.7 | 197 |
| Kericho | 58.6 | 141 | * | 141 | 90.4 | 141 | 49.0 | 141 | * | 141 | * | 79 | 87.5 | 141 | 93.7 | 141 | 89.2 | 131 | * | 131 |
| Bomet | 53.3 | 128 | * | 128 | 90.8 | 128 | 43.6 | 128 | * | 128 | * | 63 | 79.5 | 128 | 69.5 | 128 | 90.8 | 118 | * | 118 |
| Kakamega | 73.3 | 287 | * | 287 | 91.5 | 287 | 69.3 | 287 | 30.0 | 287 | 32.1 | 152 | 85.4 | 287 | 89.9 | 287 | 90.4 | 274 | * | 274 |
| Vihiga | 79.4 | 63 | * | 63 | 98.2 | 63 | 64.0 | 63 | 23.6 | 63 | * | 36 | 75.1 | 63 | 69.1 | 63 | 89.9 | 62 | * | 62 |
| Bungoma | 72.5 | 228 | * | 228 | 87.1 | 228 | 67.5 | 228 | 13.4 | 228 | 44.5 | 106 | 84.7 | 228 | 81.9 | 228 | 83.7 | 206 | 17.2 | 206 |
| Busia | 70.7 | 139 | * | 139 | 94.5 | 139 | 42.7 | 139 | * | 139 | 34.1 | 74 | 84.2 | 139 | 74.3 | 139 | 95.7 | 133 | * | 133 |
| Siaya | 65.1 | 119 | * | 119 | 88.9 | 119 | 62.7 | 119 | 18.8 | 119 | 55.1 | 53 | 79.0 | 119 | 82.9 | 119 | 83.3 | 109 | 17.7 | 109 |
| Kisumu | 63.3 | 172 | * | 172 | 94.8 | 172 | 65.6 | 172 | 18.6 | 172 | 37.0 | 79 | 91.9 | 172 | 92.0 | 172 | 89.1 | 166 | * | 166 |
| Homa Bay | 68.8 | 152 | * | 152 | 85.2 | 152 | 51.0 | 152 | 26.1 | 152 | 32.3 | 72 | 70.9 | 152 | 70.8 | 152 | 81.8 | 141 | 25.7 | 141 |
| Migori | 58.5 | 182 | * | 182 | 92.2 | 182 | 68.3 | 182 | 36.1 | 182 | 44.4 | 97 | 69.2 | 182 | 68.3 | 182 | 88.9 | 169 | * | 169 |
| Kisii | 62.7 | 164 | * | 164 | 94.7 | 164 | 45.1 | 164 | * | 164 | * | 94 | 80.2 | 164 | 81.7 | 164 | 80.8 | 156 | 19.2 | 156 |
| Nyamira | 65.8 | 55 | * | 55 | 78.4 | 55 | 43.2 | 55 | * | 55 | * | 32 | 61.4 | 55 | 65.8 | 55 | 88.4 | 52 | * | 52 |
| Nairobi | 80.5 | 746 | * | 746 | 90.0 | 746 | 63.2 | 746 | 21.5 | 746 | * | 403 | 89.8 | 746 | 86.6 | 746 | 55.5 | 693 | 47.7 | 693 |
| An asterisk indicates that the figure is based on 25 or fewer unweighted cases and has been suppressed.  Light gray shading indicates p<0.05.  Dark gray shading indicates p<0.001. | | | | | | | | | | | | | | | | | | | | |

*Continued…*

Supplementary Table 12: Health Delivery Platform and Nutrition Intervention Coverage during Birth and Postnatal Care by Background Characteristic — Kenya

|  | **Birth** | | | | | | **Postnatal care (PNC)** | | | | | | | |
| --- | --- | --- | --- | --- | --- | --- | --- | --- | --- | --- | --- | --- | --- | --- |
|  | **Health delivery platform: Live births delivered in a health facility** | | **Skin-to-skin contact immediately after birth** | | **Started breastfeeding within one hour of birth** | | **Health delivery platform: PNC check within two days for newborn** | | **Weighed during newborn PNC check** | | **Counseled about breastfeeding during newborn PNC check** | | **Observed breastfeeding during newborn PNC check** | |
|  | **%** | **N** | **%** | **N** | **%** | **N** | **%** | **N** | **%** | **N** | **%** | **N** | **%** | **N** |
| **Total** | 88.1 | 7,101 | 62.7 | 3,523 | 60.1 | 3,658 | 82.6 | 6,847 | 88.2 | 3,523 | 76.3 | 3,523 | 74.4 | 3,523 |
| **Background characteristic** |  |  |  |  |  |  |  |  |  |  |  |  |  |  |
| **Sex of child** | p=0.602 | | p=0.333 | | p=0.994 | | p=0.867 | | p=0.546 | | p=0.892 | | p=0.359 | |
| Male | 87.9 | 3,599 | 61.7 | 1,749 | 60.1 | 1,816 | 82.5 | 3,479 | 87.9 | 1,749 | 76.2 | 1,749 | 75.2 | 1,749 |
| Female | 88.3 | 3,502 | 63.7 | 1,775 | 60.1 | 1,842 | 82.7 | 3,369 | 88.5 | 1,775 | 76.4 | 1,775 | 73.6 | 1,775 |
| **Maternal age** | p=0.000 | | p=0.003 | | p=0.292 | | p=0.036 | | p=0.008 | | p=0.405 | | p=0.815 | |
| <20 | 88.9 | 618 | 68.4 | 299 | 55.5 | 302 | 82.2 | 606 | 87.3 | 299 | 73.3 | 299 | 74.4 | 299 |
| 20–34 | 89.1 | 5,352 | 63.5 | 2,671 | 60.2 | 2,792 | 83.3 | 5,140 | 89.1 | 2,671 | 76.9 | 2,671 | 74.7 | 2,671 |
| 35–49 | 83.1 | 1,131 | 55.7 | 554 | 62.2 | 564 | 79.5 | 1,101 | 84.4 | 554 | 75.3 | 554 | 73.2 | 554 |
| **Mother's education** | p=0.000 | | p=0.000 | | p=0.202 | | p=0.000 | | p=0.000 | | p=0.000 | | p=0.000 | |
| None | 49.6 | 666 | 48.5 | 340 | 65.6 | 354 | 54.1 | 639 | 46.9 | 340 | 42.5 | 340 | 40.5 | 340 |
| Primary | 85.5 | 2,501 | 63.5 | 1,232 | 60.4 | 1,268 | 79.6 | 2,417 | 85.7 | 1,232 | 73.6 | 1,232 | 71.8 | 1,232 |
| Secondary+ | 96.3 | 3,934 | 64.7 | 1,951 | 59.0 | 2,037 | 89.2 | 3,792 | 97.0 | 1,951 | 83.9 | 1,951 | 82.0 | 1,951 |
| **Place of residence** | p=0.000 | | p=0.093 | | p=0.002 | | p=0.000 | | p=0.000 | | p=0.000 | | p=0.000 | |
| Urban | 97.0 | 2,562 | 65.4 | 1,273 | 54.8 | 1,315 | 88.1 | 2,470 | 97.3 | 1,273 | 84.3 | 1,273 | 80.4 | 1,273 |
| Rural | 83.1 | 4,539 | 61.2 | 2,251 | 63.1 | 2,343 | 79.4 | 4,377 | 83.1 | 2,251 | 71.8 | 2,251 | 71.0 | 2,251 |
| **Urban poverty household** | p=0.000 | | p=0.152 | | p=0.000 | | p=0.000 | | p=0.000 | | p=0.000 | | p=0.000 | |
| Urban non-poor | 98.0 | 1,882 | 66.3 | 937 | 50.9 | 964 | 88.6 | 1,822 | 98.3 | 937 | 86.2 | 937 | 81.8 | 937 |
| Urban poor | 94.5 | 681 | 63.0 | 336 | 65.6 | 351 | 86.6 | 648 | 94.5 | 336 | 79.0 | 336 | 76.7 | 336 |
| Rural | 83.1 | 4,539 | 61.2 | 2,251 | 63.1 | 2,343 | 79.4 | 4,377 | 83.1 | 2,251 | 71.8 | 2,251 | 71.0 | 2,251 |
| **Wealth quintile** | p=0.000 | | p=0.000 | | p=0.000 | | p=0.000 | | p=0.000 | | p=0.000 | | p=0.000 | |
| Lowest | 65.8 | 1,593 | 53.4 | 765 | 64.5 | 793 | 67.5 | 1,538 | 62.9 | 765 | 55.7 | 765 | 53.2 | 765 |
| Second | 87.9 | 1,296 | 66.2 | 648 | 62.5 | 679 | 82.0 | 1,244 | 88.6 | 648 | 74.5 | 648 | 75.6 | 648 |
| Middle | 93.5 | 1,284 | 66.8 | 630 | 66.7 | 655 | 84.0 | 1,234 | 94.1 | 630 | 79.3 | 630 | 79.8 | 630 |
| Fourth | 97.4 | 1,466 | 63.9 | 772 | 60.5 | 801 | 88.6 | 1,414 | 97.9 | 772 | 83.7 | 772 | 79.5 | 772 |
| Highest | 98.5 | 1,462 | 64.6 | 709 | 46.9 | 730 | 92.1 | 1,417 | 99.4 | 709 | 89.6 | 709 | 86.1 | 709 |
| **Region** | p=0.000 | | p=0.000 | | p=0.000 | | p=0.000 | | p=0.000 | | p=0.000 | | p=0.000 | |
| Mombasa | 95.5 | 187 | 53.3 | 94 | 52.8 | 105 | 80.6 | 170 | 97.1 | 94 | 71.1 | 94 | 64.8 | 94 |
| Kwale | 84.1 | 119 | 71.3 | 60 | 75.7 | 64 | 85.6 | 113 | 89.0 | 60 | 93.7 | 60 | 95.2 | 60 |
| Kilifi | 84.5 | 191 | 67.1 | 93 | 60.2 | 95 | 89.9 | 188 | 93.3 | 93 | 70.4 | 93 | 63.6 | 93 |
| Tana River | 51.6 | 56 | 33.4 | 29 | 81.9 | 29 | 59.4 | 55 | 47.9 | 29 | 63.2 | 29 | 57.9 | 29 |
| Lamu | 90.3 | 29 | 49.2 | 16 | 34.8 | 17 | 89.6 | 27 | 89.5 | 16 | 70.1 | 16 | 67.0 | 16 |
| Taita Taveta | 93.8 | 54 | * | 24 | * | 25 | 91.8 | 52 | 97.6 | 24 | 95.4 | 24 | 95.6 | 24 |
| Garissa | 61.4 | 94 | 50.5 | 47 | 61.5 | 51 | 38.4 | 85 | 58.1 | 47 | 48.2 | 47 | 42.1 | 47 |
| Wajir | 53.6 | 56 | 49.9 | 27 | 67.2 | 29 | 37.8 | 52 | 30.9 | 27 | 21.6 | 27 | 20.3 | 27 |
| Mandera | 50.4 | 95 | 55.6 | 47 | 66.3 | 50 | 44.5 | 89 | 35.5 | 47 | 28.9 | 47 | 26.5 | 47 |
| Marsabit | 59.3 | 54 | 53.6 | 28 | 74.2 | 28 | 49.8 | 53 | 45.7 | 28 | 28.7 | 28 | 27.8 | 28 |
| Isiolo | 83.7 | 37 | 60.8 | 21 | 67.7 | 21 | 76.9 | 36 | 80.0 | 21 | 48.9 | 21 | 53.7 | 21 |
| Meru | 92.1 | 207 | * | 98 | 66.8 | 99 | 75.1 | 206 | 93.7 | 98 | 57.3 | 98 | 63.7 | 98 |
| Tharaka-Nithi | 94.5 | 50 | * | 26 | 58.0 | 26 | 85.4 | 49 | 96.0 | 26 | 69.9 | 26 | 62.5 | 26 |
| Embu | 95.3 | 69 | 66.6 | 31 | 68.5 | 33 | 96.6 | 64 | 99.1 | 31 | 89.6 | 31 | 89.1 | 31 |
| Kitui | 79.5 | 145 | * | 72 | 53.6 | 72 | 84.4 | 142 | 75.1 | 72 | 80.4 | 72 | 81.8 | 72 |
| Machakos | 95.3 | 166 | 62.7 | 76 | 56.0 | 85 | 93.5 | 154 | 93.7 | 76 | 90.5 | 76 | 92.7 | 76 |
| Makueni | 90.0 | 126 | 76.8 | 60 | 68.0 | 65 | 88.4 | 121 | 92.1 | 60 | 79.3 | 60 | 78.1 | 60 |
| Nyandarua | 97.1 | 84 | 48.5 | 47 | 63.8 | 48 | 89.4 | 82 | 100.0 | 47 | 77.5 | 47 | 73.4 | 47 |
| Nyeri | 99.1 | 88 | 69.8 | 49 | 51.3 | 52 | 87.5 | 85 | 100.0 | 49 | 85.0 | 49 | 82.4 | 49 |
| Kirinyaga | 97.3 | 84 | 77.9 | 45 | * | 47 | 96.5 | 81 | 100.0 | 45 | 95.9 | 45 | 90.8 | 45 |
| Murang'a | 96.2 | 142 | 67.4 | 69 | 71.6 | 72 | 89.6 | 134 | 97.4 | 69 | 95.4 | 69 | 92.5 | 69 |
| Kiambu | 97.7 | 420 | 62.0 | 199 | 42.9 | 203 | 92.1 | 412 | 100.0 | 199 | 94.2 | 199 | 94.8 | 199 |
| Turkana | 49.5 | 130 | 34.4 | 64 | 59.2 | 66 | 62.1 | 126 | 46.6 | 64 | 34.3 | 64 | 36.4 | 64 |
| West Pokot | 59.8 | 177 | 77.1 | 87 | 66.1 | 89 | 77.2 | 174 | 56.6 | 87 | 55.2 | 87 | 55.7 | 87 |
| Samburu | 55.4 | 62 | 55.8 | 29 | 78.6 | 30 | 60.0 | 59 | 50.6 | 29 | 32.3 | 29 | 31.3 | 29 |
| Trans Nzoia | 92.9 | 137 | 58.0 | 77 | 58.4 | 78 | 89.4 | 135 | 95.0 | 77 | 77.9 | 77 | 81.7 | 77 |
| Uasin Gishu | 94.3 | 219 | 57.0 | 106 | 69.6 | 112 | 76.0 | 210 | 94.7 | 106 | 81.1 | 106 | 86.1 | 106 |
| Elgeyo-Marakwet | 95.5 | 69 | 56.9 | 33 | 82.6 | 35 | 90.7 | 67 | 92.7 | 33 | 73.3 | 33 | 71.7 | 33 |
| Nandi | 85.6 | 118 | 82.6 | 58 | 46.0 | 60 | 80.5 | 116 | 85.7 | 58 | 76.7 | 58 | 77.3 | 58 |
| Baringo | 79.3 | 103 | 52.2 | 52 | 81.2 | 53 | 84.7 | 99 | 75.8 | 52 | 75.7 | 52 | 77.7 | 52 |
| Laikipia | 90.0 | 65 | 87.1 | 32 | 77.3 | 33 | 82.2 | 64 | 89.0 | 32 | 78.4 | 32 | 84.4 | 32 |
| Nakuru | 94.0 | 342 | 63.9 | 166 | 57.0 | 170 | 88.9 | 334 | 94.5 | 166 | 78.7 | 166 | 78.3 | 166 |
| Narok | 69.3 | 242 | 56.8 | 119 | 74.2 | 122 | 72.5 | 235 | 67.2 | 119 | 62.8 | 119 | 53.7 | 119 |
| Kajiado | 84.6 | 205 | 43.3 | 102 | 42.1 | 103 | 85.8 | 204 | 88.8 | 102 | 72.2 | 102 | 69.8 | 102 |
| Kericho | 90.9 | 147 | 76.6 | 79 | 54.2 | 84 | 68.1 | 141 | 91.9 | 79 | 89.1 | 79 | 90.4 | 79 |
| Bomet | 86.9 | 137 | 77.3 | 63 | 55.1 | 68 | 83.9 | 128 | 92.6 | 63 | 69.6 | 63 | 58.0 | 63 |
| Kakamega | 96.3 | 298 | 73.2 | 152 | 56.1 | 158 | 76.2 | 287 | 100.0 | 152 | 78.9 | 152 | 76.4 | 152 |
| Vihiga | 96.2 | 67 | 62.0 | 36 | 87.2 | 39 | 96.7 | 63 | 96.9 | 36 | 79.0 | 36 | 75.6 | 36 |
| Bungoma | 87.6 | 243 | 67.3 | 106 | 68.1 | 112 | 74.9 | 228 | 82.2 | 106 | 79.0 | 106 | 75.8 | 106 |
| Busia | 84.7 | 145 | 72.3 | 74 | 82.1 | 77 | 91.9 | 139 | 83.7 | 74 | 83.7 | 74 | 81.9 | 74 |
| Siaya | 90.3 | 125 | 63.4 | 53 | 55.1 | 55 | 85.9 | 119 | 90.1 | 53 | 65.1 | 53 | 62.4 | 53 |
| Kisumu | 96.1 | 177 | 72.7 | 79 | * | 80 | 90.1 | 172 | 95.9 | 79 | 91.2 | 79 | 87.7 | 79 |
| Homa Bay | 91.5 | 156 | 61.8 | 72 | 53.5 | 73 | 78.4 | 152 | 88.8 | 72 | 74.3 | 72 | 71.9 | 72 |
| Migori | 92.1 | 190 | 80.9 | 97 | 78.6 | 99 | 84.9 | 182 | 94.6 | 97 | 57.9 | 97 | 64.0 | 97 |
| Kisii | 93.5 | 168 | 68.5 | 94 | 82.2 | 96 | 91.0 | 164 | 89.5 | 94 | 81.7 | 94 | 78.6 | 94 |
| Nyamira | 93.7 | 57 | 70.3 | 32 | 63.3 | 35 | 87.7 | 55 | 95.1 | 32 | 74.6 | 32 | 82.1 | 32 |
| Nairobi | 99.4 | 773 | 67.1 | 403 | 52.6 | 415 | 90.6 | 746 | 100.0 | 403 | 92.7 | 403 | 84.9 | 403 |
| An asterisk indicates that the figure is based on 25 or fewer unweighted cases and has been suppressed.  Light gray shading indicates p<0.05.  Dark gray shading indicates p<0.001. | | | | | | | | | | | | | | |

*Continued…*

Supplementary Table 13: Health Delivery Platform and Nutrition Intervention Coverage during Infant and Young Childhood by Background Characteristic - Kenya

|  | **Infancy and childhood** | | | | | | | | | | | | | | | |
| --- | --- | --- | --- | --- | --- | --- | --- | --- | --- | --- | --- | --- | --- | --- | --- | --- |
|  | **Health Delivery platform: All basic vaccinations^ according to either source^&^ (12–35 mos)** | | **Mothers of children age 6–23 mos who received IYCF counseling in last 6 mos** | | **Child under 5 with weight measured in the last 3 mos** | | **Child under 5 with height measured in the last 3 mos** | | **Child under 5 with MUAC measured in the last 3 mos** | | **Children age 6–59 mos given iron containing supplements** | | **Children age 6–59 mos given Vit. A supplements** | | **Children age 12–59 mos given deworming medication** | |
|  | **%** | **N** | **%** | **N** | **%** | **N** | **%** | **N** | **%** | **N** | **%** | **N** | **%** | **N** | **%** | **N** |
| **Total** | 65.1 | 6,554 | 24.9 | 2,501 | 45.0 | 16,883 | 37.5 | 16,883 | 16.3 | 16,883 | 23.0 | 15,148 | 63.6 | 15,148 | 65.5 | 13,310 |
| **Background characteristic** |  |  |  |  |  |  |  |  |  |  |  |  |  |  |  |  |
| **Sex of child** | p=0.876 | | p=0.318 | | p=0.358 | | p=0.728 | | p=0.772 | | p=0.796 | | p=0.476 | | p=0.936 | |
| Male | 65.2 | 3,349 | 26.1 | 1,236 | 45.5 | 8,589 | 37.6 | 8,589 | 16.4 | 8,589 | 22.9 | 7,719 | 63.9 | 7,719 | 65.4 | 6,776 |
| Female | 64.9 | 3,205 | 23.8 | 1,265 | 44.6 | 8,294 | 37.3 | 8,294 | 16.2 | 8,294 | 23.1 | 7,429 | 63.2 | 7,429 | 65.5 | 6,534 |
| **Child's age in months** |  |  |  |  | p=0.000 | | p=0.000 | | p=0.000 | |  |  |  |  |  |  |
| 0–23 |  |  |  |  | 70.8 | 6,897 | 59.4 | 6,897 | 22.0 | 6,897 |  |  |  |  |  |  |
| 24–59 |  |  |  |  | 27.2 | 9,986 | 22.3 | 9,986 | 12.4 | 9,986 |  |  |  |  |  |  |
| **Maternal age** | p=0.113 | | p=0.812 | | p=0.000 | | p=0.000 | | p=0.431 | | p=0.198 | | p=0.000 | | p=0.000 | |
| <20 | 59.5 | 313 | 23.9 | 190 | 60.7 | 784 | 47.0 | 784 | 14.8 | 784 | 21.4 | 604 | 68.9 | 604 | 51.3 | 392 |
| 20–34 | 64.9 | 5,001 | 25.3 | 1,886 | 45.9 | 12,506 | 38.4 | 12,506 | 16.6 | 12,506 | 23.5 | 11,202 | 64.5 | 11,202 | 66.3 | 9,842 |
| 35–49 | 67.3 | 1,240 | 23.8 | 424 | 38.6 | 3,593 | 32.2 | 3,593 | 15.6 | 3,593 | 21.6 | 3,342 | 59.6 | 3,342 | 64.5 | 3,076 |
| **Mother's education** | p=0.000 | | p=0.000 | | p=0.000 | | p=0.000 | | p=0.000 | | p=0.457 | | p=0.000 | | p=0.000 | |
| None | 43.0 | 670 | 14.2 | 248 | 33.7 | 1,738 | 30.1 | 1,738 | 21.6 | 1,738 | 22.4 | 1,575 | 45.3 | 1,575 | 35.8 | 1,415 |
| Primary | 65.7 | 2,423 | 23.5 | 914 | 38.8 | 6,374 | 32.2 | 6,374 | 14.3 | 6,374 | 22.3 | 5,796 | 61.9 | 5,796 | 60.8 | 5,179 |
| Secondary+ | 68.9 | 3,461 | 27.9 | 1,340 | 51.8 | 8,772 | 42.7 | 8,772 | 16.7 | 8,772 | 23.6 | 7,777 | 68.5 | 7,777 | 75.3 | 6,716 |
| **Place of residence** | p=0.000 | | p=0.328 | | p=0.000 | | p=0.000 | | p=0.000 | | p=0.044 | | p=0.000 | | p=0.000 | |
| Urban | 59.6 | 2,478 | 26.7 | 892 | 50.0 | 6,316 | 44.1 | 6,316 | 19.7 | 6,316 | 24.7 | 5,699 | 67.9 | 5,699 | 72.4 | 5,051 |
| Rural | 68.4 | 4,076 | 24.0 | 1,609 | 42.0 | 10,567 | 33.5 | 10,567 | 14.3 | 10,567 | 21.9 | 9,449 | 61.0 | 9,449 | 61.2 | 8,259 |
| **Urban poverty household** | p=0.000 | | p=0.461 | | p=0.000 | | p=0.000 | | p=0.000 | | p=0.110 | | p=0.000 | | p=0.000 | |
| Urban non-poor | 60.7 | 1,896 | 27.8 | 658 | 50.8 | 4,760 | 44.6 | 4,760 | 20.2 | 4,760 | 24.8 | 4,323 | 68.4 | 4,323 | 74.2 | 3,841 |
| Urban poor | 56.1 | 581 | 23.6 | 235 | 47.7 | 1,557 | 42.6 | 1,557 | 18.2 | 1,557 | 24.7 | 1,376 | 66.5 | 1,376 | 66.5 | 1,211 |
| Rural | 68.4 | 4,076 | 24.0 | 1,609 | 42.0 | 10,567 | 33.5 | 10,567 | 14.3 | 10,567 | 21.9 | 9,449 | 61.0 | 9,449 | 61.2 | 8,259 |
| **Wealth quintile** | p=0.000 | | p=0.025 | | p=0.000 | | p=0.000 | | p=0.000 | | p=0.052 | | p=0.000 | | p=0.000 | |
| Lowest | 61.2 | 1,481 | 17.9 | 574 | 36 | 3,784 | 30.0 | 3,784 | 15.7 | 3,784 | 20.5 | 3,414 | 53.4 | 3,414 | 48.1 | 2,996 |
| Second | 71.2 | 1,176 | 26.2 | 457 | 40.8 | 3,038 | 32.6 | 3,038 | 12.2 | 3,038 | 21.9 | 2,697 | 62.3 | 2,697 | 62.2 | 2,399 |
| Middle | 68.9 | 1,118 | 25.7 | 434 | 45.2 | 2,955 | 36.4 | 2,955 | 14.0 | 2,955 | 22.7 | 2,630 | 66.8 | 2,630 | 67.1 | 2,290 |
| Fourth | 64.4 | 1,299 | 26.0 | 527 | 49.4 | 3,410 | 41.4 | 3,410 | 17.6 | 3,410 | 24.2 | 3,037 | 67.0 | 3,037 | 71.8 | 2,634 |
| Highest | 61.9 | 1,480 | 30.0 | 508 | 53.6 | 3,697 | 46.4 | 3,697 | 20.9 | 3,697 | 25.5 | 3,370 | 69.3 | 3,370 | 78.6 | 2,991 |
| **Region** | p=0.000 | | p=0.000 | | p=0.000 | | p=0.000 | | p=0.000 | | p=0.000 | | p=0.000 | | p=0.000 | |
| Mombasa | 60.9 | 164 | * | 58 | 54.0 | 429 | 47.3 | 429 | 33.0 | 429 | 44.7 | 379 | 74.5 | 379 | 77.4 | 329 |
| Kwale | 63.1 | 125 | * | 41 | 54.8 | 296 | 55.0 | 296 | 48.7 | 296 | 60.3 | 267 | 80.0 | 267 | 67.9 | 241 |
| Kilifi | 72.4 | 181 | * | 70 | 39.9 | 494 | 38.0 | 494 | 22.3 | 494 | * | 446 | 69.7 | 446 | 67.1 | 396 |
| Tana River | 58.2 | 51 | * | 21 | 39.8 | 137 | 39.0 | 137 | 26.8 | 137 | 14.9 | 124 | 38.3 | 124 | 42.0 | 108 |
| Lamu | 61.9 | 23 | * | 10 | 34.9 | 62 | 24.9 | 62 | 11.2 | 62 | * | 54 | 54.5 | 54 | 64.6 | 47 |
| Taita Taveta | 67.8 | 50 | * | 19 | 63.7 | 123 | 56.8 | 123 | 29.5 | 123 | 23.9 | 112 | 70.8 | 112 | 71.4 | 99 |
| Garissa | 20.3 | 82 | * | 29 | 58.1 | 233 | 58.8 | 233 | 47.6 | 233 | 52.0 | 204 | 33.9 | 204 | 26.7 | 181 |
| Wajir | 37.3 | 54 | * | 18 | 20.6 | 143 | 18.7 | 143 | 17.1 | 143 | 6.5 | 127 | 33.1 | 127 | 24.0 | 116 |
| Mandera | 27.2 | 95 | * | 34 | 6.8 | 246 | 5.8 | 246 | * | 246 | 10.5 | 221 | 16.4 | 221 | 15.2 | 199 |
| Marsabit | 37.3 | 51 | * | 19 | 45.0 | 130 | 40.9 | 130 | 40.4 | 130 | 17.1 | 114 | 59.5 | 114 | 35.1 | 100 |
| Isiolo | 49.6 | 38 | * | 17 | 33.8 | 94 | 26.5 | 94 | 22.1 | 94 | 33.7 | 86 | 52.7 | 86 | 41.9 | 76 |
| Meru | 70.4 | 172 | * | 81 | 27.5 | 461 | 14.3 | 461 | * | 461 | * | 410 | 49.3 | 410 | 57.8 | 351 |
| Tharaka-Nithi | 78.2 | 50 | * | 18 | 44.4 | 125 | 32.3 | 125 | * | 125 | 14.7 | 113 | 74.4 | 113 | 65.5 | 99 |
| Embu | 79.8 | 61 | * | 20 | 62.5 | 163 | 54.7 | 163 | 38.4 | 163 | 51.4 | 142 | 80.2 | 142 | 86.3 | 128 |
| Kitui | 66.2 | 137 | * | 58 | 43.2 | 334 | 40.7 | 334 | 24.1 | 334 | 22.1 | 310 | 53.9 | 310 | 45.7 | 270 |
| Machakos | 78.5 | 142 | * | 51 | 56.2 | 379 | 50.8 | 379 | 14.3 | 379 | 21.4 | 335 | 70.6 | 335 | 71.9 | 285 |
| Makueni | 70.5 | 122 | * | 45 | 43.6 | 291 | 41.7 | 291 | 17.7 | 291 | 14.0 | 261 | 50.8 | 261 | 48.7 | 230 |
| Nyandarua | 85.3 | 70 | * | 33 | 39.2 | 193 | 27.7 | 193 | * | 193 | 11.8 | 174 | 42.7 | 174 | 69.9 | 157 |
| Nyeri | 69.8 | 87 | * | 32 | 50.0 | 222 | 39.1 | 222 | * | 222 | 24.3 | 198 | 73.9 | 198 | 85.2 | 172 |
| Kirinyaga | 68.3 | 74 | * | 34 | 50.6 | 198 | 33.8 | 198 | 17.1 | 198 | 21.5 | 182 | 70.0 | 182 | 78.2 | 162 |
| Murang'a | 71.4 | 108 | * | 41 | 58.6 | 317 | 45.8 | 317 | 11.8 | 317 | 27.7 | 275 | 78.9 | 275 | 80.9 | 234 |
| Kiambu | 68.1 | 404 | * | 140 | 47.8 | 1,058 | 41.3 | 1,058 | * | 1,058 | 20.8 | 952 | 77.4 | 952 | 83.2 | 842 |
| Turkana | 58.9 | 116 | 44.4 | 44 | 63.9 | 299 | 56.2 | 299 | 41.3 | 299 | 30.0 | 269 | 68.1 | 269 | 63.6 | 240 |
| West Pokot | 33.4 | 164 |  | 61 | 20.2 | 403 | 18.5 | 403 | 5.9 | 403 | 43.0 | 363 | 48.3 | 363 | 29.0 | 314 |
| Samburu | 57.2 | 55 |  | 23 | 30.1 | 144 | 23.8 | 144 | 13.9 | 144 | * | 129 | 41.2 | 129 | 34.4 | 111 |
| Trans Nzoia | 82.5 | 136 | 46.8 | 60 | 38.4 | 348 | 23.4 | 348 | 7.8 | 348 | 34.2 | 321 | 80.1 | 321 | 80.2 | 275 |
| Uasin Gishu | 66.1 | 200 | * | 73 | 47.8 | 490 | 35.1 | 490 | * | 490 | 27.6 | 439 | 70.2 | 439 | 76.0 | 377 |
| Elgeyo-Marakwet | 58.4 | 64 | * | 24 | 34.4 | 160 | 27.8 | 160 | 8.9 | 160 | 38.4 | 144 | 56.4 | 144 | 48.0 | 129 |
| Nandi | 73.4 | 108 | * | 41 | 36.0 | 289 | 28.3 | 289 | 16.4 | 289 | 12.1 | 264 | 50.0 | 264 | 72.6 | 228 |
| Baringo | 67.1 | 89 | * | 39 | 56.8 | 243 | 49.1 | 243 | 12.4 | 243 | 19.5 | 219 | 64.8 | 219 | 61.6 | 192 |
| Laikipia | 58.8 | 66 | * | 24 | 34.3 | 155 | 25.6 | 155 | * | 155 | * | 140 | 70.6 | 140 | 74.7 | 128 |
| Nakuru | 81.6 | 344 | * | 132 | 38.5 | 853 | 23.7 | 853 | * | 853 | 15.0 | 781 | 58.3 | 781 | 73.0 | 688 |
| Narok | 62.5 | 211 | * | 72 | 32.0 | 554 | 20.7 | 554 | * | 554 | * | 479 | 46.2 | 479 | 59.7 | 415 |
| Kajiado | 50.9 | 189 | * | 71 | 45.8 | 511 | 40.7 | 511 | 15.4 | 511 | 21.9 | 463 | 60.6 | 463 | 58.9 | 403 |
| Kericho | 65.1 | 144 | * | 54 | 78.1 | 360 | 76.8 | 360 | 52.1 | 360 | 34.1 | 322 | 77.5 | 322 | 77.4 | 288 |
| Bomet | 75.7 | 126 | * | 44 | 6.3 | 325 | * | 325 | * | 325 | * | 283 | 69.9 | 283 | 83.2 | 259 |
| Kakamega | 80.2 | 266 | * | 102 | 55.2 | 609 | 45.5 | 609 | 7.1 | 609 | 33.8 | 536 | 77.8 | 536 | 74.0 | 464 |
| Vihiga | 85.1 | 60 | * | 26 | 48.7 | 159 | 36.7 | 159 | 9.3 | 159 | 20.4 | 144 | 59.4 | 144 | 77.4 | 125 |
| Bungoma | 78.3 | 224 | * | 74 | 37.1 | 561 | 17.8 | 561 | * | 561 | 28.6 | 501 | 72.9 | 501 | 81.5 | 447 |
| Busia | 73.8 | 108 | * | 45 | 56.0 | 317 | 44.7 | 317 | 7.8 | 317 | 13.0 | 274 | 54.9 | 274 | 48.1 | 228 |
| Siaya | 76.6 | 124 | * | 43 | 38.4 | 302 | 37.2 | 302 | 30.0 | 302 | 36.1 | 282 | 60.3 | 282 | 48.3 | 240 |
| Kisumu | 64.3 | 165 | * | 62 | 44.6 | 413 | 42.9 | 413 | 23.3 | 413 | 17.0 | 374 | 59.1 | 374 | 51.3 | 326 |
| Homa Bay | 64.1 | 129 | * | 50 | 54.2 | 360 | 34.3 | 360 | 8.7 | 360 | 18.1 | 318 | 67.0 | 318 | 58.2 | 274 |
| Migori | 72.6 | 168 | * | 63 | 47.2 | 422 | 40.1 | 422 | * | 422 | 13.2 | 369 | 54.8 | 369 | 51.0 | 329 |
| Kisii | 73.4 | 133 | * | 63 | 44.3 | 368 | 24.6 | 368 | * | 368 | 31.1 | 315 | 49.0 | 315 | 51.4 | 276 |
| Nyamira | 69.8 | 44 | * | 22 | 43.7 | 129 | 29.0 | 129 | * | 129 | 10.2 | 115 | 74.5 | 115 | 66.9 | 97 |
| Nairobi | 50.1 | 781 | * | 299 | 53.0 | 1,982 | 51.9 | 1,982 | 29.8 | 1,982 | 25.5 | 1,817 | 69.9 | 1,817 | 75.4 | 1,636 |
| An asterisk indicates that the figure is based on 25 or fewer unweighted cases and has been suppressed. | | | | | | | | | | | | | | | | |
| ^All basic antigen vaccines: 1 dose BCG, 3 doses Polio (OPV/IPV), 3 doses DPT, 1 dose measles (MR) | | | | | | | | | | | | | | | | |
| & either source - vaccination card or mother's report (crude coverage)  Light gray shading indicates p<0.05.  Dark gray shading indicates p<0.001. | | | | | | | | | | | | | | | | |

Supplementary Table 14: Health Delivery Platform and Nutrition Intervention Coverage during Antenatal Care by Background Characteristic - Mozambique

|  | **Pregnancy/antenatal care (ANC)** | | | | | | | | | | | | | | | | | | | |
| --- | --- | --- | --- | --- | --- | --- | --- | --- | --- | --- | --- | --- | --- | --- | --- | --- | --- | --- | --- | --- |
|  | **Health delivery platform: Attended 4+ ANC visits** | | **Health delivery platform: Attended 8+ ANC visits** | | **Took iron-containing supplements for any number of days during most recent pregnancy** | | **Took iron-containing supplements for 90+ days during most recent pregnancy** | | **Took iron-containing supplements for 180+ days during most recent pregnancy** | | **Took intestinal parasite drugs during most recent pregnancy** | | **Counseled about maternal diet during ANC visit** | | **Counseled about breastfeeding during ANC visit** | | **Source of iron-containing supplements: public sector** | | **Source of iron-containing supplements: private sector + other** | |
|  | **%** | **N** | **%** | **N** | **%** | **N** | **%** | **N** | **%** | **N** | **%** | **N** | **%** | **N** | **%** | **N** | **%** | **N** | **%** | **N** |
| **Total** | 48.6 | 3,822 | 1.9 | 3,822 | 62.2 | 3,822 | 27.0 | 3,822 | 4.8 | 3,822 | 40.8 | 3,822 | 50.8 | 3,822 | 48.2 | 3,822 | 98.9 | 3,007 | 1.2 | 3,007 |
| **Background characteristic** |  |  |  |  |  |  |  |  |  |  |  |  |  |  |  |  |  |  |  |  |
| **Sex of child** | p=0.901 | | p=0.203 | | p=0.678 | | p=0.047 | | p=0.736 | | p=0.724 | | p=0.785 | | p=0.303 | | p=0.605 | | p=0.761 | |
| Male | 48.5 | 1,883 | 2.2 | 1,883 | 62.6 | 1,883 | 28.7 | 1,883 | 4.9 | 1,883 | 40.4 | 1,883 | 51.1 | 1,883 | 47.1 | 1,883 | 98.8 | 1,481 | * | 1,481 |
| Female | 48.7 | 1,939 | 1.7 | 1,939 | 61.8 | 1,939 | 25.5 | 1,939 | 4.6 | 1,939 | 41.1 | 1,939 | 50.5 | 1,939 | 49.3 | 1,939 | 99.0 | 1,526 | * | 1,526 |
| **Maternal age** | p=0.038 | | p=0.928 | | p=0.936 | | p=0.071 | | p=0.406 | | p=0.000 | | p=0.065 | | p=0.075 | | p=0.074 | | p=0.058 | |
| <20 | 45.9 | 629 | * | 629 | 61.9 | 629 | 24.2 | 629 | 3.6 | 629 | 39.1 | 629 | 46.4 | 629 | 43.6 | 629 | 100.0 | 484 | * | 484 |
| 20–34 | 50.4 | 2,575 | 2.0 | 2,575 | 62.5 | 2,575 | 28.6 | 2,575 | 5.0 | 2,575 | 41.1 | 2,575 | 52.4 | 2,575 | 49.8 | 2,575 | 98.6 | 2,056 | 1.5 | 2,056 |
| 35–49 | 43.8 | 618 | * | 618 | 61.5 | 618 | 23.7 | 618 | 5.0 | 618 | 41.3 | 618 | 48.8 | 618 | 46.2 | 618 | 98.9 | 467 | * | 467 |
| **Mother's education** | p=0.000 | | p=0.000 | | p=0.003 | | p=0.000 | | p=0.000 | | p=0.000 | | p=0.000 | | p=0.000 | | p=0.000 | | p=0.000 | |
| None | 34.7 | 1,117 | * | 1,117 | 57.5 | 1,117 | 19.8 | 1,117 | 2.8 | 1,117 | 33.2 | 1,117 | 43.5 | 1,117 | 40.0 | 1,117 | 99.6 | 765 | * | 765 |
| Primary | 47.2 | 1,864 | 1.2 | 1,864 | 62.7 | 1,864 | 27.5 | 1,864 | 4.0 | 1,864 | 38.7 | 1,864 | 47.8 | 1,864 | 46.5 | 1,864 | 99.5 | 1,472 | * | 1,472 |
| Secondary+ | 70.2 | 841 | 4.7 | 841 | 67.5 | 841 | 35.8 | 841 | 9.2 | 841 | 55.4 | 841 | 67.1 | 841 | 63.0 | 841 | 97.1 | 770 | 3.1 | 770 |
| **Place of residence** | p=0.000 | | p=0.000 | | p=0.000 | | p=0.000 | | p=0.001 | | p=0.000 | | p=0.000 | | p=0.000 | | p=0.008 | | p=0.005 | |
| Urban | 68.0 | 1,065 | 4.0 | 1,065 | 69.4 | 1,065 | 33.5 | 1,065 | 7.0 | 1,065 | 54.0 | 1,065 | 70.4 | 1,065 | 65.5 | 1,065 | 98.0 | 952 | * | 952 |
| Rural | 41.1 | 2,757 | 1.2 | 2,757 | 59.4 | 2,757 | 24.6 | 2,757 | 3.9 | 2,757 | 35.7 | 2,757 | 43.2 | 2,757 | 41.5 | 2,757 | 99.3 | 2,055 | * | 2,055 |
| **Urban poverty household** | p=0.000 | | p=0.000 | | p=0.000 | | p=0.000 | | p=0.000 | | p=0.000 | | p=0.000 | | p=0.000 | | p=0.000 | | p=0.000 | |
| Urban non-poor | 75.1 | 604 | 5.0 | 604 | 68.5 | 604 | 36.4 | 604 | 9.0 | 604 | 58.2 | 604 | 73.4 | 604 | 68.7 | 604 | 96.8 | 562 | * | 562 |
| Urban poor | 58.8 | 461 | * | 461 | 70.7 | 461 | 29.8 | 461 | 4.5 | 461 | 48.5 | 461 | 66.3 | 461 | 61.3 | 461 | 99.6 | 389 | * | 389 |
| Rural | 41.1 | 2,757 | 1.2 | 2,757 | 59.4 | 2,757 | 24.6 | 2,757 | 3.9 | 2,757 | 35.7 | 2,757 | 43.2 | 2,757 | 41.5 | 2,757 | 99.3 | 2,055 | * | 2,055 |
| **Wealth Quintile** | p=0.000 | | p=0.000 | | p=0.000 | | p=0.000 | | p=0.000 | | p=0.000 | | p=0.000 | | p=0.000 | | p=0.000 | | p=0.000 | |
| Lowest | 30.8 | 993 | * | 993 | 54.2 | 993 | 22.3 | 993 | 2.6 | 993 | 32.1 | 993 | 36.3 | 993 | 30.8 | 993 | 99.6 | 652 | * | 652 |
| Second | 39.3 | 865 | * | 865 | 59.3 | 865 | 19.7 | 865 | * | 865 | 32.9 | 865 | 45.7 | 865 | 42.4 | 865 | 99.1 | 621 | * | 621 |
| Middle | 49.1 | 723 | * | 723 | 64.6 | 723 | 30.6 | 723 | 5.6 | 723 | 38.7 | 723 | 49.0 | 723 | 52.3 | 723 | 99.7 | 591 | * | 591 |
| Fourth | 63.6 | 757 | * | 757 | 69.5 | 757 | 31.0 | 757 | 5.2 | 757 | 49.3 | 757 | 63.3 | 757 | 61.3 | 757 | 99.3 | 679 | * | 679 |
| Highest | 77.7 | 485 | 7 | 485 | 69.0 | 485 | 38.5 | 485 | 10.9 | 485 | 62.3 | 485 | 72.9 | 485 | 67.4 | 485 | 96.0 | 464 | * | 464 |
| **Region** | p=0.000 | | p=0.000 | | p=0.000 | | p=0.000 | | p=0.000 | | p=0.000 | | p=0.000 | | p=0.000 | | p=0.001 | | p=0.000 | |
| Niassa | 49.2 | 331 | * | 331 | 72.7 | 331 | 35.8 | 331 | * | 331 | 46.5 | 331 | 37.4 | 331 | 44.0 | 331 | 99.5 | 264 | * | 264 |
| Cabo Delgado | 53.4 | 277 | * | 277 | 87.9 | 277 | 17.9 | 277 | * | 277 | 62.1 | 277 | 75.6 | 277 | 67.8 | 277 | 100.0 | 258 | * | 258 |
| Nampula | 37.7 | 1,023 | * | 1,023 | 69.8 | 1,023 | 26.0 | 1,023 | * | 1,023 | 30.5 | 1,023 | 48.8 | 1,023 | 47.3 | 1,023 | 99.4 | 728 | * | 728 |
| Zambézia | 25.8 | 692 | * | 692 | 36.6 | 692 | 13.2 | 692 | * | 692 | 31.3 | 692 | 37.5 | 692 | 25.1 | 692 | 98.3 | 420 | * | 420 |
| Tete | 46.6 | 391 | * | 391 | 71.3 | 391 | 23.7 | 391 | 5.6 | 391 | 45.5 | 391 | 58.7 | 391 | 58.6 | 391 | 98.8 | 302 | * | 302 |
| Manica | 65.5 | 294 | * | 294 | 20.0 | 294 | 10.7 | 294 | * | 294 | 45.5 | 294 | 57.3 | 294 | 59.7 | 294 | 100.0 | 262 | * | 262 |
| Sofala | 68.7 | 270 | * | 270 | 70.0 | 270 | 45.5 | 270 | * | 270 | 32.2 | 270 | 60.7 | 270 | 51.1 | 270 | 98.9 | 249 | * | 249 |
| Inhambane | 68.1 | 124 | * | 124 | 91.7 | 124 | 55 | 124 | 11.5 | 124 | 55.0 | 124 | 44.4 | 124 | 45.8 | 124 | 99.7 | 118 | * | 118 |
| Gaza | 77.0 | 147 | * | 147 | 90.7 | 147 | 64.1 | 147 | 15.9 | 147 | 41.0 | 147 | 40.5 | 147 | 59.2 | 147 | 98.7 | 141 | * | 141 |
| Maputo | 82.7 | 190 | * | 190 | 48.3 | 190 | 29.1 | 190 | * | 190 | 59.6 | 190 | 56.9 | 190 | 48.5 | 190 | 95.5 | 182 | * | 182 |
| Cidade de Maputo | 81.2 | 84 | * | 84 | 74.1 | 84 | 52.8 | 84 | 20.7 | 84 | 76.6 | 84 | 77.8 | 84 | 86.2 | 84 | 94.7 | 83 | * | 83 |
| An asterisk indicates that the figure is based on 25 or fewer unweighted cases and has been suppressed.  Light gray shading indicates p<0.05.  Dark gray shading indicates p<0.001. | | | | | | | | | | | | | | | | | | | | |

*Continued…*

Supplementary Table 15: Health Delivery Platform and Nutrition Intervention Coverage during Birth and Postnatal Care by Background Characteristic - Mozambique

|  | **Birth** | | | | | | **Postnatal care (PNC)** | | | | | | | |
| --- | --- | --- | --- | --- | --- | --- | --- | --- | --- | --- | --- | --- | --- | --- |
|  | **Health delivery platform: Live births delivered in a health facility** | | **Skin-to-skin contact immediately after birth** | | **Started breastfeeding within one hour of birth** | | **Health delivery platform: PNC check within two days for newborn** | | **Weighed during newborn PNC check** | | **Counseled about breastfeeding during newborn PNC check** | | **Observed breastfeeding during newborn PNC check** | |
|  | **%** | **N** | **%** | **N** | **%** | **N** | **%** | **N** | **%** | **N** | **%** | **N** | **%** | **N** |
| **Total** | 64.6 | 3,926 | 45.6 | 3,822 | 77.1 | 3,926 | 40.9 | 3,822 | 60.7 | 3,822 | 38.7 | 3,822 | 34.5 | 3,822 |
| **Background characteristic** |  |  |  |  |  |  |  |  |  |  |  |  |  |  |
| **Sex of child** | p=0.112 | | p=0.551 | | p=0.558 | | p=0.639 | | p=0.792 | | p=0.477 | | p=0.705 | |
| Male | 66.1 | 1,940 | 46.2 | 1,883 | 77.6 | 1,940 | 41.4 | 1,883 | 60.9 | 1,883 | 39.4 | 1,883 | 34.2 | 1,883 |
| Female | 63.1 | 1,986 | 45.0 | 1,939 | 76.6 | 1,986 | 40.5 | 1,939 | 60.4 | 1,939 | 38.0 | 1,939 | 34.9 | 1,939 |
| **Maternal age** | p=0.031 | | p=0.590 | | p=0.056 | | p=0.743 | | p=0.001 | | p=0.330 | | p=0.009 | |
| <20 | 67.8 | 642 | 47.2 | 629 | 72.5 | 642 | 42.7 | 629 | 64.4 | 629 | 37.6 | 629 | 33.8 | 629 |
| 20–34 | 65.2 | 2,643 | 45.7 | 2,575 | 78.2 | 2,643 | 40.5 | 2,575 | 61.9 | 2,575 | 39.6 | 2,575 | 36.2 | 2,575 |
| 35–49 | 58.8 | 641 | 43.5 | 618 | 77.3 | 641 | 40.9 | 618 | 51.6 | 618 | 35.8 | 618 | 28.4 | 618 |
| **Mother's education** | p=0.000 | | p=0.548 | | p=0.000 | | p=0.000 | | p=0.000 | | p=0.000 | | p=0.000 | |
| None | 51.6 | 1,151 | 45.5 | 1,117 | 81.2 | 1,151 | 35.3 | 1,117 | 43.6 | 1,117 | 30.2 | 1,117 | 25.8 | 1,117 |
| Primary | 61.2 | 1,917 | 44.6 | 1,864 | 78.7 | 1,917 | 38.9 | 1,864 | 58.3 | 1,864 | 36.6 | 1,864 | 32.5 | 1,864 |
| Secondary+ | 89.7 | 858 | 47.8 | 841 | 68.0 | 858 | 53.0 | 841 | 88.5 | 841 | 54.4 | 841 | 50.7 | 841 |
| **Place of residence** | p=0.000 | | p=0.003 | | p=0.077 | | p=0.000 | | p=0.000 | | p=0.000 | | p=0.000 | |
| Urban | 90.3 | 1,098 | 52.4 | 1,065 | 74.3 | 1,098 | 50.0 | 1,065 | 87.4 | 1,065 | 51.5 | 1,065 | 48.4 | 1,065 |
| Rural | 54.6 | 2,828 | 42.9 | 2,757 | 78.2 | 2,828 | 37.4 | 2,757 | 50.3 | 2,757 | 33.7 | 2,757 | 29.2 | 2,757 |
| **Urban poverty household** | p=0.000 | | p=0.006 | | p=0.000 | | p=0.000 | | p=0.000 | | p=0.000 | | p=0.000 | |
| Urban non-poor | 95.4 | 623 | 51.2 | 604 | 67.7 | 623 | 55.3 | 604 | 91.9 | 604 | 57.0 | 604 | 53.6 | 604 |
| Urban poor | 83.6 | 475 | 53.9 | 461 | 82.8 | 475 | 43.1 | 461 | 81.5 | 461 | 44.3 | 461 | 41.6 | 461 |
| Rural | 54.6 | 2,828 | 42.9 | 2,757 | 78.2 | 2,828 | 37.4 | 2,757 | 50.3 | 2,757 | 33.7 | 2,757 | 29.2 | 2,757 |
| **Wealth Quintile** | p=0.000 | | p=0.017 | | p=0.000 | | p=0.000 | | p=0.000 | | p=0.000 | | p=0.000 | |
| Lowest | 39.1 | 1,021 | 39.0 | 993 | 84.7 | 1,021 | 25.1 | 993 | 35.5 | 993 | 23.2 | 993 | 19.0 | 993 |
| Second | 50.3 | 881 | 46.2 | 865 | 82.1 | 881 | 36.9 | 865 | 47.5 | 865 | 32.2 | 865 | 28.0 | 865 |
| Middle | 67.6 | 739 | 45.1 | 723 | 73.2 | 739 | 44.5 | 723 | 63.7 | 723 | 42.1 | 723 | 35.6 | 723 |
| Fourth | 90.4 | 783 | 51.7 | 757 | 75.0 | 783 | 50.6 | 757 | 84.9 | 757 | 49.1 | 757 | 48.3 | 757 |
| Highest | 96.9 | 502 | 48.9 | 485 | 61.9 | 502 | 60.1 | 485 | 93.1 | 485 | 60.4 | 485 | 55.0 | 485 |
| **Region** | p=0.000 | | p=0.000 | | p=0.000 | | p=0.000 | | p=0.000 | | p=0.000 | | p=0.000 | |
| Niassa | 77.2 | 348 | 25.6 | 331 | 75.2 | 348 | 56.9 | 331 | 65.9 | 331 | 34.6 | 331 | 26.9 | 331 |
| Cabo Delgado | 57.7 | 283 | 68.5 | 277 | 89.8 | 283 | 52.0 | 277 | 60.8 | 277 | 57.5 | 277 | 53.1 | 277 |
| Nampula | 52.4 | 1,043 | 66.3 | 1,023 | 92.9 | 1,043 | 18.9 | 1,023 | 52.3 | 1,023 | 21.4 | 1,023 | 21.8 | 1,023 |
| Zambézia | 48.1 | 708 | 25.4 | 692 | 87.0 | 708 | 21.2 | 692 | 39.7 | 692 | 25.8 | 692 | 19.9 | 692 |
| Tete | 65.3 | 403 | 52.6 | 391 | 62.1 | 403 | 69.0 | 391 | 53.9 | 391 | 56.0 | 391 | 52.1 | 391 |
| Manica | 75.2 | 305 | 31.3 | 294 | 74.9 | 305 | 28.9 | 294 | 73.4 | 294 | 52.7 | 294 | 48.4 | 294 |
| Sofala | 78.5 | 276 | 61.6 | 270 | 77.4 | 276 | 65.4 | 270 | 80.7 | 270 | 51.5 | 270 | 35.7 | 270 |
| Inhambane | 81.7 | 125 | 26.2 | 124 | 41.6 | 125 | 51.1 | 124 | 81.0 | 124 | 58.3 | 124 | 56.3 | 124 |
| Gaza | 87.2 | 149 | 11.5 | 147 | 30.0 | 149 | 72.3 | 147 | 87.4 | 147 | 47.5 | 147 | 39.8 | 147 |
| Maputo | 97.3 | 196 | 36.8 | 190 | 45.3 | 196 | 78.9 | 190 | 89.5 | 190 | 48.2 | 190 | 54.4 | 190 |
| Cidade de Maputo | 94.5 | 88 | 34.9 | 84 | 53 | 88 | 50.2 | 84 | 94.2 | 84 | 71.0 | 84 | 58.9 | 84 |

Light gray shading indicates p<0.05.

Dark gray shading indicates p<0.001.

*Continued…*

Supplementary Table 16: Health Delivery Platform and Nutrition Intervention Coverage during Infant and Young Childhood by Background Characteristic —Mozambique

|  | **Infancy and childhood** | | | | | | | | | | | | | | | |
| --- | --- | --- | --- | --- | --- | --- | --- | --- | --- | --- | --- | --- | --- | --- | --- | --- |
|  | **Health Delivery platform: All basic vaccinations^ according to either source^&^ (12–35 mos)** | | **Mothers of children age 6–23 mos who received IYCF counseling in last 6 mos** | | **Child under 5 with weight measured in the last 3 mos** | | **Child under 5 with height measured in the last 3 mos** | | **Child under 5 with MUAC measured in the last 3 mos** | | **Children age 6–59 mos given iron containing supplements** | | **Children age 6–59 mos given Vit. A supplements** | | **Children age 12–59 mos given deworming medication** | |
|  | **%** | **N** | **%** | **N** | **%** | **N** | **%** | **N** | **%** | **N** | **%** | **N** | **%** | **N** | **%** | **N** |
| **Total** | 37.7 | 3,758 | 11.5 | 2,677 | 38.8 | 9,396 | 37.3 | 9,396 | 35.1 | 9,396 | 31.4 | 8,382 | 50.1 | 8,382 | 35.6 | 7,448 |
| **Background characteristic** |  |  |  |  |  |  |  |  |  |  |  |  |  |  |  |  |
| **Sex of child** | p=0.635 | | p=0.149 | | p=0.600 | | p=0.648 | | p=0.471 | | p=0.406 | | p=0.603 | | p=0.945 | |
| Male | 38.2 | 1,801 | 10.4 | 1,295 | 38.4 | 4,543 | 37.0 | 4,543 | 34.7 | 4,543 | 32.0 | 4,025 | 49.7 | 4,025 | 35.7 | 3,599 |
| Female | 37.3 | 1,957 | 12.5 | 1,382 | 39.1 | 4,853 | 37.6 | 4,853 | 35.5 | 4,853 | 30.9 | 4,357 | 50.4 | 4,357 | 35.6 | 3,849 |
| **Child's age in months** |  |  |  |  | p=0.000 | | p=0.000 | | p=0.000 | |  |  |  |  |  |  |
| 0–23 |  |  |  |  | 56.5 | 3,755 | 55.0 | 3,755 | 51.4 | 3,755 |  |  |  |  |  |  |
| 24–59 |  |  |  |  | 27.0 | 5,640 | 25.5 | 5,640 | 24.3 | 5,640 |  |  |  |  |  |  |
| **Maternal age** | p=0.922 | | p=0.794 | | p=0.006 | | p=0.003 | | p=0.009 | | p=0.993 | | p=0.509 | | p=0.881 | |
| <20 | 36.8 | 453 | 10.3 | 386 | 44.5 | 968 | 43.0 | 968 | 40.5 | 968 | 31.5 | 764 | 52.6 | 764 | 34.8 | 621 |
| 20–34 | 38.0 | 2,598 | 11.7 | 1,833 | 38.8 | 6,569 | 37.4 | 6,569 | 35.1 | 6,569 | 31.5 | 5,907 | 50.1 | 5,907 | 35.6 | 5,274 |
| 35–49 | 37.4 | 707 | 11.4 | 458 | 35.8 | 1,859 | 34.0 | 1,859 | 32.3 | 1,859 | 31.3 | 1,711 | 48.8 | 1,711 | 36.2 | 1,552 |
| **Mother's education** | p=0.000 | | p=0.000 | | p=0.000 | | p=0.000 | | p=0.000 | | p=0.000 | | p=0.000 | | p=0.000 | |
| None | 24.2 | 1,134 | 6.7 | 819 | 29.9 | 2,839 | 29.2 | 2,839 | 27.9 | 2,839 | 28.6 | 2,574 | 39.4 | 2,574 | 26.2 | 2,271 |
| Primary | 36.2 | 1,828 | 9.7 | 1,267 | 36.3 | 4,574 | 34.9 | 4,574 | 32.7 | 4,574 | 29.4 | 4,048 | 48.5 | 4,048 | 32.9 | 3,626 |
| Secondary+ | 60.6 | 797 | 22.0 | 591 | 57.5 | 1,983 | 54.6 | 1,983 | 51.0 | 1,983 | 40.3 | 1,760 | 69.2 | 1,760 | 56.0 | 1,551 |
| **Place of residence** | p=0.000 | | p=0.000 | | p=0.000 | | p=0.000 | | p=0.000 | | p=0.000 | | p=0.000 | | p=0.000 | |
| Urban | 56.3 | 1,067 | 20.6 | 727 | 54.0 | 2,709 | 51.6 | 2,709 | 49.1 | 2,709 | 40.5 | 2,405 | 65.4 | 2,405 | 53.1 | 2,145 |
| Rural | 30.4 | 2,691 | 8.1 | 1,950 | 32.6 | 6,687 | 31.5 | 6,687 | 29.4 | 6,687 | 27.8 | 5,977 | 43.9 | 5,977 | 28.6 | 5,303 |
| **Urban poverty household** | p=0.000 | | p=0.000 | | p=0.000 | | p=0.000 | | p=0.000 | | p=0.000 | | p=0.000 | | p=0.000 | |
| Urban non-poor | 59.9 | 616 | 27.9 | 410 | 57.3 | 1,540 | 54.5 | 1,540 | 52.0 | 1,540 | 40.3 | 1,365 | 68.8 | 1,365 | 57.5 | 1,218 |
| Urban poor | 51.4 | 451 | 11.1 | 317 | 49.7 | 1,169 | 47.8 | 1,169 | 45.3 | 1,169 | 40.8 | 1,040 | 60.8 | 1,040 | 47.3 | 927 |
| Rural | 30.4 | 2,691 | 8.1 | 1,950 | 32.6 | 6,687 | 31.5 | 6,687 | 29.4 | 6,687 | 27.8 | 5,977 | 43.9 | 5,977 | 28.6 | 5,303 |
| **Wealth Quintile** | p=0.000 | | p=0.000 | | p=0.000 | | p=0.000 | | p=0.000 | | p=0.000 | | p=0.000 | | p=0.000 | |
| Lowest | 19.6 | 948 | 6.1 | 704 | 23.8 | 2,430 | 23.5 | 2,430 | 21.6 | 2,430 | 23.6 | 2,170 | 34.5 | 2,170 | 22.2 | 1,911 |
| Second | 24.5 | 877 | 7.2 | 605 | 29.1 | 2,073 | 27.9 | 2,073 | 27.1 | 2,073 | 26.1 | 1,854 | 38.9 | 1,854 | 24.3 | 1,654 |
| Middle | 42.5 | 716 | 6.7 | 488 | 39.5 | 1,854 | 37.4 | 1,854 | 34.8 | 1,854 | 32.9 | 1,648 | 50.6 | 1,648 | 34.1 | 1,476 |
| Fourth | 54.9 | 726 | 16.3 | 549 | 54.8 | 1,794 | 52.8 | 1,794 | 49.8 | 1,794 | 38.9 | 1,598 | 66.2 | 1,598 | 49.4 | 1,407 |
| Highest | 64.1 | 492 | 29.6 | 331 | 60.1 | 1,245 | 57.5 | 1,245 | 53.9 | 1,245 | 42.8 | 1,112 | 75.0 | 1,112 | 63.2 | 1,001 |
| **Region** | p=0.000 | | p=0.000 | | p=0.000 | | p=0.000 | | p=0.000 | | p=0.000 | | p=0.000 | | p=0.000 | |
| Niassa | 39.9 | 327 | * | 234 | 32.6 | 798 | 32.3 | 798 | 30.5 | 798 | 50.8 | 708 | 54.4 | 708 | 38.5 | 626 |
| Cabo Delgado | 40.5 | 237 | 10.7 | 186 | 54.9 | 614 | 54.3 | 614 | 54.6 | 614 | 52.9 | 533 | 62.9 | 533 | 50.4 | 466 |
| Nampula | 24.5 | 954 | * | 693 | 36.0 | 2,499 | 34.2 | 2,499 | 31.9 | 2,499 | 23.0 | 2,225 | 33.9 | 2,225 | 25.2 | 1,978 |
| Zambézia | 13.8 | 750 | 14.9 | 493 | 19.8 | 1,760 | 20.1 | 1,760 | 18.7 | 1,760 | 21.9 | 1,575 | 33.5 | 1,575 | 23.5 | 1,431 |
| Tete | 34.7 | 380 | * | 298 | 40.4 | 987 | 39.4 | 987 | 38.6 | 987 | 40.2 | 901 | 58.5 | 901 | 37.6 | 780 |
| Manica | 55.9 | 287 | 25.5 | 206 | 35.8 | 723 | 33.9 | 723 | 30.0 | 723 | 17.7 | 645 | 75.3 | 645 | 37.2 | 569 |
| Sofala | 65.7 | 260 | 16.8 | 192 | 45.7 | 641 | 43.0 | 641 | 41.1 | 641 | 29.0 | 570 | 46.1 | 570 | 36.6 | 496 |
| Inhambane | 70.5 | 121 | * | 94 | 61.0 | 293 | 57.1 | 293 | 53.3 | 293 | 41.1 | 268 | 67.6 | 268 | 49.7 | 232 |
| Gaza | 68.9 | 146 | 18.2 | 103 | 57.2 | 357 | 56.8 | 357 | 54.7 | 357 | 44.8 | 319 | 78.2 | 319 | 58.4 | 288 |
| Maputo | 69.4 | 209 | * | 121 | 62.1 | 510 | 57.2 | 510 | 49.7 | 510 | 33.7 | 447 | 74.5 | 447 | 59.3 | 405 |
| Cidade de Maputo | 69.7 | 88 | 47.7 | 57 | 69.4 | 214 | 63.8 | 214 | 59.8 | 214 | 47.4 | 193 | 79.5 | 193 | 76.5 | 176 |
| An asterisk indicates that the figure is based on 25 or fewer unweighted cases and has been suppressed. | | | | | | | | | | | | | | | | |
| ^All basic antigen vaccines: 1 dose BCG, 3 doses Polio (OPV/IPV), 3 doses DPT, 1 dose measles (MR) | | | | | | | | | | | | | | | | |
| & either source - vaccination card or mother's report (crude coverage)  Light gray shading indicates p<0.05.  Dark gray shading indicates p<0.001. | | | | | | | | | | | | | | | | |

Supplementary Table 17: Health Delivery Platform and Nutrition Intervention Coverage during Antenatal Care by Background Characteristic - Tanzania

|  | **Pregnancy/antenatal care (ANC)** | | | | | | | | | | | | | | | | | | | |
| --- | --- | --- | --- | --- | --- | --- | --- | --- | --- | --- | --- | --- | --- | --- | --- | --- | --- | --- | --- | --- |
|  | **Health delivery platform: Attended 4+ ANC visits** | | **Health delivery platform: Attended 8+ ANC visits** | | **Took iron-containing supplements for any number of days during most recent pregnancy** | | **Took iron-containing supplements for 90+ days during most recent pregnancy** | | **Took iron-containing supplements for 180+ days during most recent pregnancy** | | **Took intestinal parasite drugs during most recent pregnancy** | | **Counseled about maternal diet during ANC visit** | | **Counseled about breastfeeding during ANC visit** | | **Source of iron-containing supplements: public sector** | | **Source of iron-containing supplements: private sector + other** | |
|  | **%** | **N** | **%** | **N** | **%** | **N** | **%** | **N** | **%** | **N** | **%** | **N** | **%** | **N** | **%** | **N** | **%** | **N** | **%** | **N** |
| **Total** | 65.1 | 4,335 | 3.1 | 4,335 | 79.9 | 4,335 | 41.0 | 4,335 | 8.4 | 4,335 | 61.8 | 4,335 | 62.0 | 4,335 | 60.3 | 4,335 | 90.2 | 3,533 | 10.7 | 3,533 |
| **Background characteristic** |  |  |  |  |  |  |  |  |  |  |  |  |  |  |  |  |  |  |  |  |
| **Sex of child** | p=0.300 | | p=0.692 | | p=0.056 | | p=0.235 | | p=0.326 | | p=0.805 | | p=0.952 | | p=0.367 | | p=0.984 | | p=0.477 | |
| Male | 64.2 | 2,233 | 3.3 | 2,233 | 78.5 | 2,233 | 40.0 | 2,233 | 7.9 | 2,233 | 62.0 | 2,233 | 62.0 | 2,233 | 59.6 | 2,233 | 90.2 | 1,785 | 10.2 | 1,785 |
| Female | 66.0 | 2,102 | 2.9 | 2,102 | 81.4 | 2,102 | 42.1 | 2,102 | 9.0 | 2,102 | 61.6 | 2,102 | 62.1 | 2,102 | 61.1 | 2,102 | 90.3 | 1,748 | 11.1 | 1,748 |
| **Maternal age** | p=0.014 | | p=0.254 | | p=0.624 | | p=0.072 | | p=0.842 | | p=0.146 | | p=0.632 | | p=0.582 | | p=0.004 | | p=0.077 | |
| <20 | 65.6 | 428 | * | 428 | 77.9 | 428 | 35.7 | 428 | 7.5 | 428 | 56.1 | 428 | 59.3 | 428 | 59.8 | 428 | 94.9 | 337 | * | 337 |
| 20–34 | 66.4 | 3,035 | 3.4 | 3,035 | 79.9 | 3,035 | 42.3 | 3,035 | 8.5 | 3,035 | 62.7 | 3,035 | 62.4 | 3,035 | 60.9 | 3,035 | 90.6 | 2,477 | 10.4 | 2,477 |
| 35–49 | 60.2 | 871 | * | 871 | 80.8 | 871 | 39.1 | 871 | 8.8 | 871 | 61.3 | 871 | 62.0 | 871 | 58.6 | 871 | 86.8 | 719 | 13.4 | 719 |
| **Mother's education** | p=0.000 | | p=0.000 | | p=0.000 | | p=0.000 | | p=0.000 | | p=0.000 | | p=0.000 | | p=0.000 | | p=0.000 | | p=0.000 | |
| None | 53.5 | 894 | * | 894 | 72.6 | 894 | 32.9 | 894 | 4.4 | 894 | 53.0 | 894 | 49.3 | 894 | 50.2 | 894 | 94.5 | 671 | 5.9 | 671 |
| Primary | 63.0 | 2,397 | 2.3 | 2,397 | 80.3 | 2,397 | 38.9 | 2,397 | 6.9 | 2,397 | 63.8 | 2,397 | 60.7 | 2,397 | 58.5 | 2,397 | 91.4 | 1,959 | 9.4 | 1,959 |
| Secondary+ | 79.7 | 1,044 | 6.3 | 1,044 | 85.1 | 1,044 | 52.8 | 1,044 | 15.4 | 1,044 | 64.8 | 1,044 | 76.0 | 1,044 | 73.2 | 1,044 | 84.6 | 903 | 16.8 | 903 |
| **Place of residence** | p=0.000 | | p=0.000 | | p=0.204 | | p=0.000 | | p=0.000 | | p=0.000 | | p=0.000 | | p=0.000 | | p=0.000 | | p=0.000 | |
| Urban | 75.9 | 1,193 | 7.4 | 1,193 | 81.8 | 1,193 | 48.3 | 1,193 | 14.4 | 1,193 | 68.8 | 1,193 | 72.2 | 1,193 | 70.2 | 1,193 | 84.2 | 1,001 | 17.9 | 1,001 |
| Rural | 61.0 | 3,142 | 1.5 | 3,142 | 79.2 | 3,142 | 38.2 | 3,142 | 6.2 | 3,142 | 59.1 | 3,142 | 58.2 | 3,142 | 56.6 | 3,142 | 92.7 | 2,532 | 7.8 | 2,532 |
| **Urban poverty household** | p=0.000 | | p=0.000 | | p=0.222 | | p=0.000 | | p=0.000 | | p=0.000 | | p=0.000 | | p=0.000 | | p=0.000 | | p=0.000 | |
| Urban non-poor | 78.9 | 980 | 8.5 | 980 | 82.7 | 980 | 50.2 | 980 | 15.7 | 980 | 70.2 | 980 | 73.3 | 980 | 71.3 | 980 | 84.5 | 832 | 17.8 | 832 |
| Urban poor | 62.2 | 213 | * | 213 | 77.6 | 213 | 39.4 | 213 | * | 213 | 62.2 | 213 | 67.0 | 213 | 65.1 | 213 | 82.5 | 169 | * | 169 |
| Rural | 61.0 | 3,142 | 1.5 | 3,142 | 79.2 | 3,142 | 38.2 | 3,142 | 6.2 | 3,142 | 59.1 | 3,142 | 58.2 | 3,142 | 56.6 | 3,142 | 92.7 | 2,532 | 7.8 | 2,532 |
| **Wealth quintile** | p=0.000 | | p=0.000 | | p=0.002 | | p=0.000 | | p=0.000 | | p=0.000 | | p=0.000 | | p=0.000 | | p=0.000 | | p=0.000 | |
| Lowest | 51.4 | 980 | * | 980 | 76.4 | 980 | 32.2 | 980 | 4.1 | 980 | 52.7 | 980 | 50.3 | 980 | 49.9 | 980 | 95.8 | 767 | 4.4 | 767 |
| Second | 59.0 | 865 | * | 865 | 79.7 | 865 | 37.5 | 865 | 6.3 | 865 | 59.3 | 865 | 56.8 | 865 | 53.0 | 865 | 92.7 | 697 | 8.0 | 697 |
| Middle | 63.5 | 838 | * | 838 | 80.8 | 838 | 42.6 | 838 | 6.6 | 838 | 62.5 | 838 | 59.7 | 838 | 58.6 | 838 | 91.0 | 689 | 9.8 | 689 |
| Fourth | 71.8 | 850 | * | 850 | 77.7 | 850 | 40.4 | 850 | 8.0 | 850 | 62.6 | 850 | 67.7 | 850 | 64.9 | 850 | 89.1 | 681 | 12.1 | 681 |
| Highest | 83.0 | 801 | 9.7 | 801 | 85.6 | 801 | 54.5 | 801 | 18.4 | 801 | 74.2 | 801 | 78.4 | 801 | 78.1 | 801 | 82.1 | 699 | 19.6 | 699 |
| **Region** | p=0.000 | | p=0.000 | | p=0.000 | | p=0.000 | | p=0.000 | | p=0.000 | | p=0.000 | | p=0.000 | | p=0.000 | | p=0.000 | |
| Dodoma | 76.8 | 189 | * | 189 | 78.7 | 189 | 42.3 | 189 | * | 189 | 69.9 | 189 | 74.7 | 189 | 66.1 | 189 | 93.6 | 149 | * | 149 |
| Arusha | 66.3 | 141 | * | 141 | 83.0 | 141 | 61.1 | 141 | * | 141 | 42.7 | 141 | 60.2 | 141 | 58.3 | 141 | 80.7 | 117 | 20.2 | 117 |
| Kilimanjaro | 63.4 | 102 | * | 102 | 86.6 | 102 | 49.9 | 102 | * | 102 | 84.8 | 102 | 69.1 | 102 | 70.7 | 102 | 87.3 | 88 | * | 88 |
| Tanga | 58.2 | 220 | * | 220 | 88.1 | 220 | 65.0 | 220 | * | 220 | 70.9 | 220 | 42.4 | 220 | 43.2 | 220 | 98.0 | 202 | * | 202 |
| Morogoro | 82.3 | 209 | * | 209 | 89.2 | 209 | 43.3 | 209 | * | 209 | 60.1 | 209 | 66.8 | 209 | 57.9 | 209 | 90.7 | 187 | * | 187 |
| Pwani | 60.4 | 116 | * | 116 | 85.5 | 116 | 49.6 | 116 | * | 116 | 79.6 | 116 | 54.4 | 116 | 46.7 | 116 | 87.8 | 103 | * | 103 |
| Dar es Salaam | 90.2 | 250 | * | 250 | 83.1 | 250 | 70.1 | 250 | 32.6 | 250 | 75.8 | 250 | 87.5 | 250 | 83.9 | 250 | 87.8 | 210 | * | 210 |
| Lindi | 69.5 | 81 | * | 81 | 90.4 | 81 | 46.1 | 81 | * | 81 | 70.8 | 81 | 66.9 | 81 | 71.3 | 81 | 98.7 | 73 | * | 73 |
| Mtwara | 82.5 | 93 | * | 93 | 87.4 | 93 | 54.1 | 93 | * | 93 | 73.0 | 93 | 83.7 | 93 | 75.4 | 93 | 97.3 | 81 | * | 81 |
| Ruvuma | 77.9 | 108 | * | 108 | 90.5 | 108 | 55.6 | 108 | * | 108 | 75.1 | 108 | 79.6 | 108 | 80.4 | 108 | 86.1 | 101 | * | 101 |
| Iringa | 72.5 | 80 | * | 80 | 93.9 | 80 | 76.9 | 80 | * | 80 | 95.1 | 80 | 87.0 | 80 | 85.8 | 80 | 89.0 | 75 | * | 75 |
| Mbeya | 68.2 | 126 | * | 126 | 89.7 | 126 | 40.9 | 126 | * | 126 | 60.6 | 126 | 68.9 | 126 | 70.8 | 126 | 92.5 | 113 | * | 113 |
| Singida | 59.7 | 105 | * | 105 | 73.3 | 105 | 36.2 | 105 | * | 105 | 66.9 | 105 | 69.0 | 105 | 56.7 | 105 | 98.6 | 80 | * | 80 |
| Tabora | 50.3 | 270 | * | 270 | 81.4 | 270 | 26.4 | 270 | * | 270 | 53.9 | 270 | 41.3 | 270 | 35.4 | 270 | 98.7 | 222 | * | 222 |
| Rukwa | 51.4 | 114 | * | 114 | 73.5 | 114 | 26.0 | 114 | * | 114 | 60.8 | 114 | 27.3 | 114 | 28.1 | 114 | 97.0 | 86 | * | 86 |
| Kigoma | 60.8 | 175 | * | 175 | 87.0 | 175 | 23.3 | 175 | * | 175 | 56.2 | 175 | 55.5 | 175 | 52.8 | 175 | 93.5 | 156 | * | 156 |
| Shinyanga | 44.9 | 159 | * | 159 | 57.7 | 159 | 35.1 | 159 | * | 159 | 57.7 | 159 | 43.9 | 159 | 50.3 | 159 | 92.1 | 99 | * | 99 |
| Kagera | 71.5 | 264 | * | 264 | 67.8 | 264 | 37.2 | 264 | * | 264 | 57.2 | 264 | 69.1 | 264 | 69.7 | 264 | 69.6 | 181 | 30.4 | 181 |
| Mwanza | 66.2 | 353 | * | 353 | 80.1 | 353 | 31.7 | 353 | 14.2 | 353 | 50.7 | 353 | 71.7 | 353 | 72.4 | 353 | 90.0 | 294 | * | 294 |
| Mara | 75.1 | 255 | * | 255 | 77.9 | 255 | 33.4 | 255 | * | 255 | 63.6 | 255 | 49.1 | 255 | 43.3 | 255 | 98.3 | 199 | * | 199 |
| Manyara | 49.9 | 135 | * | 135 | 81.6 | 135 | 39.5 | 135 | * | 135 | 54.5 | 135 | 52.7 | 135 | 44.6 | 135 | 92.6 | 110 | * | 110 |
| Njombe | 49.9 | 45 | * | 45 | 93.0 | 45 | 58.6 | 45 | * | 45 | 86.3 | 45 | 63.1 | 45 | 60.7 | 45 | 94.0 | 42 | * | 42 |
| Katavi | 41.6 | 71 | * | 71 | 77.2 | 71 | 23.5 | 71 | * | 71 | 57.9 | 71 | 39.5 | 71 | 43.5 | 71 | 93.5 | 56 | * | 56 |
| Simiyu | 36.0 | 154 | * | 154 | 67.2 | 154 | 28.0 | 154 | * | 154 | 45.0 | 154 | 43.8 | 154 | 52.3 | 154 | 95.6 | 110 | * | 110 |
| Geita | 56.4 | 286 | * | 286 | 69.6 | 286 | 30.8 | 286 | * | 286 | 61.0 | 286 | 63.7 | 286 | 70.5 | 286 | 74.1 | 202 | 26.4 | 202 |
| Songwe | 70.6 | 108 | * | 108 | 80.5 | 108 | 34.7 | 108 | * | 108 | 60.6 | 108 | 67.2 | 108 | 70.1 | 108 | 93.1 | 88 | * | 88 |
| Kaskazini Unguja | 69.1 | 18 | * | 18 | 71.4 | 18 | 27.0 | 18 | * | 18 | 31.1 | 18 | 88.9 | 18 | 70.9 | 18 | 76.1 | 13 | * | 13 |
| Kusini Unguja | 84.1 | 11 | * | 11 | 91.8 | 11 | 55.4 | 11 | * | 11 | 35.1 | 11 | 86.1 | 11 | 76.2 | 11 | 86.3 | 10 | * | 10 |
| Mjini Magharibi | 86.5 | 59 | * | 59 | 79.3 | 59 | 30.0 | 59 | * | 59 | 42.0 | 59 | 90.9 | 59 | 79.4 | 59 | 81.0 | 54 | 20.0 | 54 |
| Kaskazini Pemba | 76.1 | 16 | * | 16 | 72.9 | 16 | 27.0 | 16 | * | 16 | 46.1 | 16 | 80.8 | 16 | 79.4 | 16 | 87.2 | 13 | * | 13 |
| Kusini Pemba | 69.1 | 23 | * | 23 | 82.5 | 23 | 23.1 | 23 | * | 23 | 28.3 | 23 | 82.6 | 23 | 81.2 | 23 | 90.2 | 20 | * | 20 |
| An asterisk indicates that the figure is based on 25 or fewer unweighted cases and has been suppressed.  Light gray shading indicates p<0.05.  Dark gray shading indicates p<0.001. | | | | | | | | | | | | | | | | | | | | |

*Continued…*

Supplementary Table 18: Health Delivery Platform and Nutrition Intervention Coverage during Birth and Postnatal Care by Background Characteristic - Tanzania

|  | **Birth** | | | | | | **Postnatal care (PNC)** | | | | | | | |
| --- | --- | --- | --- | --- | --- | --- | --- | --- | --- | --- | --- | --- | --- | --- |
|  | **Health delivery platform: Live births delivered in a health facility** | | **Skin-to-skin contact immediately after birth** | | **Started breastfeeding within one hour of birth** | | **Health delivery platform: PNC check within two days for newborn** | | **Weighed during newborn PNC check** | | **Counseled about breastfeeding during newborn PNC check** | | **Observed breastfeeding during newborn PNC check** | |
|  | **%** | **N** | **%** | **N** | **%** | **N** | **%** | **N** | **%** | **N** | **%** | **N** | **%** | **N** |
| **Total** | 81.2 | 4,506 | 55.3 | 4,335 | 71.1 | 4,506 | 53.9 | 4,335 | 79.3 | 4,335 | 49.3 | 4,335 | 50.8 | 4,335 |
| **Background characteristic** |  |  |  |  |  |  |  |  |  |  |  |  |  |  |
| **Sex of child** | p=0.527 | | p=0.747 | | p=0.389 | | p=0.519 | | p=0.726 | | p=0.738 | | p=0.706 | |
| Male | 81.6 | 2,325 | 55.0 | 2,233 | 70.4 | 2,325 | 53.3 | 2,233 | 79.5 | 2,233 | 49.0 | 2,233 | 51.1 | 2,233 |
| Female | 80.7 | 2,181 | 55.6 | 2,102 | 71.8 | 2,181 | 54.5 | 2,102 | 79.0 | 2,102 | 49.7 | 2,102 | 50.4 | 2,102 |
| **Maternal age** | p=0.178 | | p=0.117 | | p=0.089 | | p=0.104 | | p=0.205 | | p=0.707 | | p=0.400 | |
| <20 | 82.2 | 443 | 53.5 | 428 | 66.6 | 443 | 53.9 | 428 | 80.6 | 428 | 47.2 | 428 | 47.7 | 428 |
| 20–34 | 81.8 | 3,153 | 56.6 | 3,035 | 72.1 | 3,153 | 52.7 | 3,035 | 79.9 | 3,035 | 49.8 | 3,035 | 51.5 | 3,035 |
| 35–49 | 78.3 | 910 | 51.8 | 871 | 69.5 | 910 | 58.1 | 871 | 76.6 | 871 | 48.7 | 871 | 49.6 | 871 |
| **Mother's education** | p=0.000 | | p=0.000 | | p=0.011 | | p=0.000 | | p=0.000 | | p=0.000 | | p=0.000 | |
| None | 65.8 | 932 | 43.2 | 894 | 73.8 | 932 | 41.5 | 894 | 60.1 | 894 | 33.6 | 894 | 35.9 | 894 |
| Primary | 81.4 | 2,485 | 55.9 | 2,397 | 72.0 | 2,485 | 53.9 | 2,397 | 80.6 | 2,397 | 47.8 | 2,397 | 49.8 | 2,397 |
| Secondary+ | 93.8 | 1,089 | 64.5 | 1,044 | 66.6 | 1,089 | 64.7 | 1,044 | 92.6 | 1,044 | 66.2 | 1,044 | 65.7 | 1,044 |
| **Place of residence** | p=0.000 | | p=0.001 | | p=0.001 | | p=0.000 | | p=0.000 | | p=0.000 | | p=0.000 | |
| Urban | 94.3 | 1,251 | 62.1 | 1,193 | 66.1 | 1,251 | 62.8 | 1,193 | 93.2 | 1,193 | 65.6 | 1,193 | 66.0 | 1,193 |
| Rural | 76.1 | 3,255 | 52.8 | 3,142 | 73.0 | 3,255 | 50.6 | 3,142 | 74.0 | 3,142 | 43.1 | 3,142 | 45.0 | 3,142 |
| **Urban poverty household** | p=0.000 | | p=0.000 | | p=0.001 | | p=0.000 | | p=0.000 | | p=0.000 | | p=0.000 | |
| Urban non-poor | 95.4 | 1,019 | 64.6 | 980 | 67.5 | 1,019 | 63.4 | 980 | 95.3 | 980 | 66.5 | 980 | 67.4 | 980 |
| Urban poor | 89.4 | 232 | 50.6 | 213 | 59.6 | 232 | 59.7 | 213 | 83.9 | 213 | 61.5 | 213 | 59.3 | 213 |
| Rural | 76.1 | 3,255 | 52.8 | 3,142 | 73.0 | 3,255 | 50.6 | 3,142 | 74.0 | 3,142 | 43.1 | 3,142 | 45.0 | 3,142 |
| **Wealth quintile** | p=0.000 | | p=0.000 | | p=0.003 | | p=0.000 | | p=0.000 | | p=0.000 | | p=0.000 | |
| Lowest | 63.6 | 1,017 | 41.6 | 980 | 71.8 | 1,017 | 40.2 | 980 | 57.4 | 980 | 30.5 | 980 | 34.4 | 980 |
| Second | 76.3 | 898 | 52.0 | 865 | 74.1 | 898 | 51.9 | 865 | 75.6 | 865 | 43.6 | 865 | 45.7 | 865 |
| Middle | 82.1 | 873 | 58.1 | 838 | 73.8 | 873 | 56.3 | 838 | 82.8 | 838 | 48.5 | 838 | 48.5 | 838 |
| Fourth | 90.2 | 885 | 60.2 | 850 | 71.2 | 885 | 57.5 | 850 | 88.7 | 850 | 57.7 | 850 | 57.8 | 850 |
| Highest | 97.2 | 833 | 67.5 | 801 | 64.0 | 833 | 66.7 | 801 | 96.4 | 801 | 70.3 | 801 | 71.2 | 801 |
| **Region** | p=0.000 | | p=0.000 | | p=0.000 | | p=0.000 | | p=0.000 | | p=0.000 | | p=0.000 | |
| Dodoma | 90.9 | 195 | 60.0 | 189 | 71.3 | 195 | 61.0 | 189 | 92.5 | 189 | 48.7 | 189 | 54.0 | 189 |
| Arusha | 65.6 | 144 | 43.4 | 141 | 65.6 | 144 | 44.9 | 141 | 61.4 | 141 | 52.0 | 141 | 52.4 | 141 |
| Kilimanjaro | 95.2 | 107 | 57.6 | 102 | 62.9 | 107 | 75.4 | 102 | 95.7 | 102 | 66.8 | 102 | 67.1 | 102 |
| Tanga | 65.6 | 221 | 55.8 | 220 | 67.2 | 221 | 37.6 | 220 | 62.2 | 220 | 21.6 | 220 | 23.4 | 220 |
| Morogoro | 82.2 | 213 | 47.4 | 209 | 64.6 | 213 | 53.1 | 209 | 77.6 | 209 | 55.5 | 209 | 60.1 | 209 |
| Pwani | 86.6 | 119 | 59.3 | 116 | 76.2 | 119 | 43.6 | 116 | 91.4 | 116 | 45.8 | 116 | 48.7 | 116 |
| Dar es Salaam | 100.0 | 265 | 64.0 | 250 | 71.0 | 265 | 71.6 | 250 | 97.9 | 250 | 87.4 | 250 | 86.1 | 250 |
| Lindi | 96.2 | 85 | 29.3 | 81 | 85.9 | 85 | 84.8 | 81 | 86.0 | 81 | 44.5 | 81 | 43.8 | 81 |
| Mtwara | 97.4 | 95 | 41.6 | 93 | 81.6 | 95 | 69.8 | 93 | 97.7 | 93 | 48.5 | 93 | 49.8 | 93 |
| Ruvuma | 95.8 | 111 | 43.9 | 108 | 62.4 | 111 | 84.3 | 108 | 97.4 | 108 | 60.6 | 108 | 62.6 | 108 |
| Iringa | 100 | 84 | 69.4 | 80 | 83.8 | 84 | 84.9 | 80 | 96.0 | 80 | 90.8 | 80 | 87.0 | 80 |
| Mbeya | 80.9 | 130 | 33.9 | 126 | 52.2 | 130 | 47.6 | 126 | 84.9 | 126 | 64.1 | 126 | 56.9 | 126 |
| Singida | 78.0 | 107 | 50.7 | 105 | 76.0 | 107 | 37.6 | 105 | 74.0 | 105 | 27.9 | 105 | 32.8 | 105 |
| Tabora | 72.7 | 286 | 50.9 | 270 | 74.5 | 286 | 43.6 | 270 | 69.4 | 270 | 19.3 | 270 | 25.6 | 270 |
| Rukwa | 94.9 | 115 | 62.1 | 114 | 89.9 | 115 | 23.5 | 114 | 67.1 | 114 | * | 114 | 18.1 | 114 |
| Kigoma | 93.5 | 180 | 75.0 | 175 | 77.8 | 180 | 58.7 | 175 | 87.3 | 175 | 38.2 | 175 | 44.1 | 175 |
| Shinyanga | 81.1 | 167 | 50.8 | 159 | 67.3 | 167 | 55.2 | 159 | 75.1 | 159 | 44.1 | 159 | 44.5 | 159 |
| Kagera | 77.2 | 272 | 63.1 | 264 | 80.9 | 272 | 66.2 | 264 | 80.2 | 264 | 68.5 | 264 | 64.5 | 264 |
| Mwanza | 80.1 | 380 | 61.2 | 353 | 53.9 | 380 | 54.8 | 353 | 82.6 | 353 | 57.3 | 353 | 61.0 | 353 |
| Mara | 71.8 | 274 | 64.9 | 255 | 78.5 | 274 | 41.4 | 255 | 74.8 | 255 | 34.9 | 255 | 36.5 | 255 |
| Manyara | 56.2 | 140 | 30.3 | 135 | 79.3 | 140 | 41.8 | 135 | 48.0 | 135 | 33.0 | 135 | 36.9 | 135 |
| Njombe | 98.7 | 47 | 64.8 | 45 | 60.9 | 47 | 77.7 | 45 | 98.8 | 45 | 95.8 | 45 | 92.8 | 45 |
| Katavi | 66.5 | 72 | 35.2 | 71 | 69.5 | 72 | 19.7 | 71 | 61.0 | 71 | 24.4 | 71 | 28.0 | 71 |
| Simiyu | 72.5 | 160 | 38.2 | 154 | 77.2 | 160 | 46.6 | 154 | 62.4 | 154 | 54.8 | 154 | 66.5 | 154 |
| Geita | 72.1 | 294 | 63.7 | 286 | 73.3 | 294 | 50.0 | 286 | 75.8 | 286 | 50.7 | 286 | 49.5 | 286 |
| Songwe | 86.0 | 112 | 43.2 | 108 | 70.8 | 112 | 59.8 | 108 | 91.0 | 108 | 57.6 | 108 | 45.9 | 108 |
| Kaskazini Unguja | 81.7 | 19 | 81.8 | 18 | 62.6 | 19 | 70.8 | 18 | 78.9 | 18 | 46.1 | 18 | 36.0 | 18 |
| Kusini Unguja | 90.8 | 11 | 82.6 | 11 | 57.5 | 11 | 46.0 | 11 | 87.2 | 11 | 61.8 | 11 | 60.9 | 11 |
| Mjini Magharibi | 95.9 | 62 | 84.6 | 59 | 61.2 | 62 | 69.3 | 59 | 95.2 | 59 | 61.9 | 59 | 57.5 | 59 |
| Kaskazini Pemba | 76.0 | 18 | 77.6 | 16 | 57.8 | 18 | 32.2 | 16 | 72.7 | 16 | 44.1 | 16 | 43.3 | 16 |
| Kusini Pemba | 72.3 | 24 | 74.1 | 23 | 65.7 | 24 | 42.0 | 23 | 67.6 | 23 | 32.4 | 23 | 31.4 | 23 |
| An asterisk indicates that the figure is based on 25 or fewer unweighted cases and has been suppressed.  Light gray shading indicates p<0.05.  Dark gray shading indicates p<0.001. | | | | | | | | | | | | | | |

*Continued…*

Supplementary Table 19: Health Delivery Platform and Nutrition Intervention Coverage during Infant and Young Childhood by Background Characteristic - Tanzania

|  | **Infancy and childhood** | | | | | | | | | | | | | |
| --- | --- | --- | --- | --- | --- | --- | --- | --- | --- | --- | --- | --- | --- | --- |
|  | **Health Delivery platform: All basic vaccinations^ according to either source^&^ (12–35 mos)** | | **Mothers of children age 6–23 mos who received IYCF counseling in last 6 mos** | | **Child under 5 with weight measured in the last 3 mos** | | **Child under 5 with height measured in the last 3 mos** | | **Children age 6–59 mos given iron containing supplements** | | **Children age 6–59 mos given Vit. A supplements** | | **Children age 12–59 mos given deworming medication** | |
|  | **%** | **N** | **%** | **N** | **%** | **N** | **%** | **N** | **%** | **N** | **%** | **N** | **%** | **N** |
| **Total** | 55.4 | 4,190 | 17.9 | 3,090 | 67.8 | 10,497 | 8.1 | 10,497 | 10.8 | 9,382 | 53.3 | 9,382 | 49.7 | 8,309 |
| **Background characteristic** |  |  |  |  |  |  |  |  |  |  |  |  |  |  |
| **Sex of child** | p=0.302 | | p=0.335 | | p=0.566 | | p=0.955 | | p=0.775 | | p=0.540 | | p=0.337 | |
| Male | 56.3 | 2,177 | 18.6 | 1,580 | 67.5 | 5,349 | 8.1 | 5,349 | 10.7 | 4,773 | 53.7 | 4,773 | 49.0 | 4,251 |
| Female | 54.4 | 2,013 | 17.0 | 1,510 | 68.2 | 5,147 | 8.1 | 5,147 | 11.0 | 4,608 | 52.9 | 4,608 | 50.3 | 4,058 |
| **Child's age in months** |  |  |  |  | p=0.000 | | p=0.578 | |  |  |  |  |  |  |
| 0–23 |  |  |  |  | 83.5 | 4,368 | 7.8 | 4,368 |  |  |  |  |  |  |
| 24–59 |  |  |  |  | 56.6 | 6,128 | 8.2 | 6,128 |  |  |  |  |  |  |
| **Maternal age** | p=0.160 | | p=0.434 | | p=0.000 | | p=0.005 | | p=0.004 | | p=0.304 | | p=0.169 | |
| <20 | 47.1 | 208 | 14.9 | 266 | 80.7 | 548 | 5.1 | 548 | 12.2 | 395 | 55.8 | 395 | 42.3 | 271 |
| 20–34 | 55.8 | 3,003 | 17.9 | 2,172 | 68.1 | 7,345 | 8.7 | 7,345 | 11.6 | 6,579 | 53.7 | 6,579 | 50.4 | 5,828 |
| 35–49 | 55.9 | 979 | 18.9 | 652 | 64.3 | 2,603 | 7.0 | 2,603 | 8.4 | 2,407 | 51.8 | 2,407 | 48.7 | 2,210 |
| **Mother's education** | p=0.000 | | p=0.000 | | p=0.000 | | p=0.000 | | p=0.000 | | p=0.000 | | p=0.000 | |
| None | 44.5 | 887 | 11.7 | 644 | 58.5 | 2,249 | 4.7 | 2,249 | 5.9 | 2,016 | 45.0 | 2,016 | 34.6 | 1,827 |
| Primary | 56.6 | 2,368 | 17.6 | 1,720 | 69.1 | 5,950 | 8.6 | 5,950 | 9.8 | 5,349 | 54.4 | 5,349 | 50.8 | 4,734 |
| Secondary+ | 62.7 | 935 | 24.0 | 726 | 73.5 | 2,297 | 10.0 | 2,297 | 18.5 | 2,017 | 58.8 | 2,017 | 62.3 | 1,748 |
| **Place of residence** | p=0.334 | | p=0.000 | | p=0.374 | | p=0.336 | | p=0.000 | | p=0.073 | | p=0.000 | |
| Urban | 57.3 | 1,144 | 25.9 | 856 | 69.2 | 2,853 | 9.1 | 2,853 | 18.0 | 2,568 | 56.4 | 2,568 | 61.2 | 2,251 |
| Rural | 54.7 | 3,045 | 14.8 | 2,234 | 67.3 | 7,643 | 7.7 | 7,643 | 8.1 | 6,814 | 52.2 | 6,814 | 45.4 | 6,058 |
| **Urban poverty household** | p=0.347 | | p=0.000 | | p=0.489 | | p=0.422 | | p=0.000 | | p=0.101 | | p=0.000 | |
| Urban non-poor | 56.4 | 966 | 27.6 | 828 | 69.4 | 2,370 | 9.4 | 2,370 | 20.3 | 2,134 | 56.3 | 2,134 | 62.2 | 1,897 |
| Urban poor | 61.8 | 178 | 18.2 | 28 | 68.1 | 483 | 7.5 | 483 | 6.8 | 433 | 56.8 | 433 | 55.9 | 355 |
| Rural | 54.7 | 3,045 | 14.8 | 2,234 | 67.3 | 7,643 | 7.7 | 7,643 | 8.1 | 6,814 | 52.2 | 6,814 | 45.4 | 6,058 |
| **Wealth quintile** | p=0.000 | | p=0.000 | | p=0.000 | | p=0.000 | | p=0.000 | | p=0.000 | | p=0.000 | |
| Lowest | 44.6 | 955 | 12.0 | 703 | 59.3 | 2,409 | 3.8 | 2,409 | 5.3 | 2,144 | 43.7 | 2,144 | 34.3 | 1,925 |
| Second | 55.5 | 845 | 11.7 | 603 | 68.8 | 2,088 | 9.1 | 2,088 | 6.7 | 1,856 | 52.9 | 1,856 | 45.7 | 1,660 |
| Middle | 59.2 | 789 | 14.9 | 591 | 71.3 | 2,001 | 9.9 | 2,001 | 8.8 | 1,779 | 55.4 | 1,779 | 49.9 | 1,548 |
| Fourth | 62.6 | 848 | 24.3 | 619 | 69.7 | 2,110 | 8.0 | 2,110 | 11.2 | 1,901 | 57.7 | 1,901 | 54.1 | 1,678 |
| Highest | 57.0 | 753 | 27.5 | 574 | 71.7 | 1,889 | 10.6 | 1,889 | 24.2 | 1,702 | 58.7 | 1,702 | 68.6 | 1,499 |
| **Region** | p=0.000 | | p=0.000 | | p=0.000 | | p=0.000 | | p=0.000 | | p=0.000 | | p=0.000 | |
| Dodoma | 69.1 | 166 | * | 137 | 75.9 | 436 | * | 436 | 24.1 | 387 | 58.4 | 387 | 61.7 | 344 |
| Arusha | 54.3 | 149 | * | 103 | 35.6 | 355 | * | 355 | 15.4 | 325 | 32.8 | 325 | 28.3 | 290 |
| Kilimanjaro | 70.3 | 90 | * | 70 | 76.2 | 243 | 12.9 | 243 | 16.9 | 215 | 60.6 | 215 | 68.8 | 182 |
| Tanga | 48.2 | 222 | * | 161 | 76.2 | 537 | * | 537 | 9.0 | 485 | 61.2 | 485 | 53.0 | 430 |
| Morogoro | 60.0 | 182 | 19.7 | 155 | 85.6 | 455 | * | 455 | * | 408 | 52.5 | 408 | 65.2 | 357 |
| Pwani | 51.1 | 120 | * | 84 | 79.8 | 320 | * | 320 | * | 290 | 63.9 | 290 | 60.6 | 263 |
| Dar es Salaam | 52.0 | 239 | 46.3 | 192 | 77.4 | 588 | * | 588 | 35.7 | 544 | 59.9 | 544 | 74.9 | 468 |
| Lindi | 53.9 | 69 | * | 58 | 89.1 | 171 | * | 171 | * | 149 | 68.2 | 149 | 67.7 | 120 |
| Mtwara | 38.2 | 87 | * | 71 | 86.3 | 215 | * | 215 | * | 195 | 74.2 | 195 | 56.1 | 172 |
| Ruvuma | 60.1 | 84 | * | 70 | 90.8 | 237 | * | 237 | * | 203 | 73.1 | 203 | 56.5 | 181 |
| Iringa | 75.4 | 82 | 42.5 | 62 | 93.6 | 181 | 76.7 | 181 | * | 164 | 90.2 | 164 | 90.5 | 150 |
| Mbeya | 57.3 | 117 | * | 85 | 72.1 | 287 | 14.0 | 287 | * | 251 | 42.8 | 251 | 49.4 | 224 |
| Singida | 66.2 | 107 | * | 77 | 60.0 | 282 | * | 282 | * | 253 | 41.8 | 253 | 32.3 | 226 |
| Tabora | 34.5 | 266 | * | 188 | 50.2 | 652 | * | 652 | * | 574 | 49.3 | 574 | 35.4 | 508 |
| Rukwa | 34.6 | 104 | 37.6 | 79 | 58.6 | 277 | 20.2 | 277 | 10.3 | 246 | 34.7 | 246 | 19.9 | 220 |
| Kigoma | 70.7 | 186 | * | 128 | 83.4 | 434 | 26.9 | 434 | 15.4 | 388 | 67.0 | 388 | 61.1 | 351 |
| Shinyanga | 36.5 | 181 | 26.2 | 115 | 41.4 | 415 | * | 415 | 22.8 | 375 | 33.6 | 375 | 31.7 | 341 |
| Kagera | 49.7 | 244 | * | 181 | 83.1 | 623 | * | 623 | 8.2 | 553 | 51.9 | 553 | 55.6 | 490 |
| Mwanza | 60.5 | 318 | * | 240 | 64.1 | 867 | 12.1 | 867 | 8.1 | 765 | 58.3 | 765 | 54.0 | 671 |
| Mara | 72.5 | 286 | * | 176 | 67.4 | 621 | * | 621 | 11.6 | 549 | 53.6 | 549 | 39.5 | 504 |
| Manyara | 48.8 | 149 | * | 107 | 50.1 | 350 | * | 350 | * | 323 | 40.6 | 323 | 24.5 | 288 |
| Njombe | 74.7 | 49 | * | 29 | 98.5 | 118 | 81.5 | 118 | * | 104 | 91 | 104 | 90.0 | 97 |
| Katavi | 41.1 | 59 | 26.4 | 49 | 61.8 | 162 | 25.6 | 162 | 21.3 | 144 | 39.1 | 144 | 33.9 | 122 |
| Simiyu | 39.1 | 132 | 30.6 | 108 | 40.2 | 373 | * | 373 | * | 334 | 28.5 | 334 | 19.0 | 287 |
| Geita | 58.0 | 271 | * | 191 | 66.3 | 718 | * | 718 | * | 635 | 49.0 | 635 | 43.9 | 568 |
| Songwe | 59.2 | 102 | 28.6 | 84 | 77.2 | 264 | * | 264 | * | 241 | 55.1 | 241 | 40.4 | 211 |
| Kaskazini Unguja | 84.0 | 15 | * | 14 | 40.9 | 44 | * | 44 | * | 41 | 48.9 | 41 | 60.4 | 33 |
| Kusini Unguja | 67.4 | 11 | * | 7 | 48.2 | 25 | * | 25 | * | 22 | 56.0 | 22 | 61.1 | 19 |
| Mjini Magharibi | 69.6 | 58 | 32.1 | 42 | 45.5 | 147 | 8.6 | 147 | 10.4 | 132 | 49.0 | 132 | 72.0 | 113 |
| Kaskazini Pemba | 73.3 | 20 | * | 12 | 30.4 | 46 | * | 46 | * | 42 | 66.4 | 42 | 71.5 | 37 |
| Kusini Pemba | 65.8 | 23 | * | 16 | 36.4 | 54 | 7.5 | 54 | * | 48 | 72.7 | 48 | 70.3 | 43 |
| An asterisk indicates that the figure is based on 25 or fewer unweighted cases and has been suppressed. | | | | | | | | | | | | | | |
| ^All basic antigen vaccines: 1 dose BCG, 3 doses Polio (OPV/IPV), 3 doses DPT, 1 dose measles (MR) | | | | | | | | | | | | | | |
| & either source - vaccination card or mother's report (crude coverage)  Light gray shading indicates p<0.05.  Dark gray shading indicates p<0.001. | | | | | | | | | | | | | | |
